# The Relative Accuracy of Different Methods for Measuring Mind Wandering Subtypes: A Systematic Review

**DOI:** 10.1002/brb3.70764

**Published:** 2025-08-22

**Authors:** Sholeh Nazari, Paul Fitzgerald, Reza Kazemi

**Affiliations:** ^1^ Faculty of Psychology and Educational Sciences University of Tehran Tehran Iran; ^2^ College of Health and Medicine The Australian National University Canberra Australia; ^3^ Faculty of Entrepreneurship University of Tehran Tehran Iran

**Keywords:** attention shift, measurement methods, mind wandering

## Abstract

**Purpose:**

Mind wandering, the shift of attention from a primary task to unrelated thoughts, is a pervasive cognitive phenomenon with significant implications for cognition, emotion, and behavior. Despite its prevalence, accurately measuring mind wandering and its subtypes remains a challenge due to its subjective and dynamic nature.

**Method:**

This systematic review evaluated the strengths and limitations of methods for measuring mind wandering subtypes, synthesizing findings from 555 studies. Questionnaires and experience sampling methods (ESMs) were most frequently used, offering high ecological validity but limited by self‐report biases. Neuroimaging techniques, such as fMRI and EEG, provided objective insights into neural correlates, particularly the default mode network (DMN), but were constrained by high costs and low ecological validity. Behavioral tasks revealed task‐related attentional lapses but lacked sensitivity to spontaneous mind wandering, while physiological measures and eye‐tracking offered unique perspectives but required complex data analysis.

**Finding:**

To address the challenges associated with the limitations of existing methodologies for assessing mind wandering, we propose the Multidimensional Assessment of Mind Wandering (MAMW) framework, which integrates diverse methodologies to provide a comprehensive understanding of mind wandering.

**Conclusion:**

The findings highlight the need for standardized measurement tools, greater ecological validity, and longitudinal research to explore the developmental and clinical trajectories of mind wandering. Future studies should prioritize integrating multiple methods to advance both theoretical knowledge and practical applications in clinical, educational, and workplace settings.

## Graphical abstract

How do we accurately measure mind wandering? This review compares five methods: from self‐reports (prone to bias) to brain scans (precise but expensive). No single method captures all aspects, so we propose the MAMW framework—a unified approach combining strengths of each technique. Findings guide future research toward more valid, practical tools for psychology, education, and mental health.

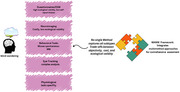

## 1 Introduction

### 1.1 Background and Context

Mind wandering, defined as the shift of attention away from a primary task to unrelated thoughts or images (Wong et al. 2023; Bozhilova 2018), is a ubiquitous cognitive phenomenon with significant implications for cognition, emotion, and behavior. It occurs during approximately 50% of waking hours (Killingsworth and Gilbert 2010), making it a prevalent and inherent aspect of human cognition. However, its subjective and dynamic nature poses methodological, conceptual, and theoretical challenges for accurate measurement and assessment (Chu et al. 2023; Weinstein 2018; Christoff et al. 2016 Smallwood and Schooler 2015). To address these challenges, it is essential to clearly define mind wandering and its subtypes, as well as to develop robust frameworks for its measurement. Supporting Information.

Mind wandering is best conceptualized as a *dimensional feature* that exists on a continuum, ranging from normal, everyday experiences to maladaptive patterns associated with *psychiatric symptoms or executive dysfunction* (Christoff et al. 2016; Smallwood and Schooler 2015). This perspective highlights the variability and fluidity of mind wandering, emphasizing that it is not a binary phenomenon but rather a spectrum that can manifest differently across individuals and contexts.

Mind wandering can be classified into subtypes based on content, function, or intentionality (Bozhilova 2018). For example: *Temporal orientation*: Future‐oriented versus past‐oriented thoughts (Smallwood and Schooler 2015). *Outcomes*: Adaptive (e.g., problem solving, creativity) versus maladaptive (e.g., boredom, distraction) (Mooneyham and Schooler 2013; Seli et al. 2016). *Control and awareness*: Spontaneous (internally driven) versus deliberate (externally triggered; Maillet et al. 2017; Seli et al. 2016).

These subtypes reflect the heterogeneity of mind wandering and underscore the need for tailored measurement approaches. For example, spontaneous mind wandering, which stems from internally driven cognitive processes, is often associated with the default mode network (DMN), while deliberate mind wandering, which arises in response to external stimuli, may involve the executive network (EN; Pavlova 2024; Menon 2023; Seli et al. 2016).

Mind wandering has significant implications for psychology and psychiatry. For instance, excessive mind wandering is considered a unique symptom of Adult ADHD according to the European Network Adult ADHD (ENAA) consensus report (Kooij et al. 2019). Recent studies have also explored its role in mediating anxiety and depression in adults with ADHD (Kandeğer et al. 2023). This highlights the clinical relevance of understanding mind wandering and its measurement, particularly in populations where it may contribute to functional impairments.

### 1.2 Rationale for the Review

Understanding mind wandering subtypes has important implications for a wide range of applications. In clinical settings, excessive mind wandering is associated with conditions such as ADHD and depression, making it a potential target for diagnostic and therapeutic interventions (Kooij et al. 2019; Kandeğer et al. 2023). In educational contexts, mind wandering can impact learning and academic performance, highlighting the need for strategies to manage attentional lapses (Mooneyham and Schooler 2013). In the workplace, mind wandering may influence productivity and decision making, underscoring the importance of understanding its mechanisms and consequences (Smallwood and Schooler 2015). By addressing these gaps, this review aims to provide a comprehensive understanding of mind wandering and its measurement, guided by the Multidimensional Assessment of Mind Wandering (MAMW) framework.

Despite the growing body of research on mind wandering, significant gaps remain in the literature (Pachai et al. 2016). Existing studies often focus on isolated aspects of mind wandering, leading to fragmented insights and conflicting findings (Yamaoka and Yukawa 2020). For example, while self‐report measures offer high ecological validity, they are prone to biases such as recall inaccuracies and social desirability effects (Koller et al. 2023). Conversely, neuroimaging techniques provide objective neural data but are often limited by cost, accessibility, and lack of ecological validity (Shareef‐Trudeau et al. 2025; Halkiopoulos et al. 2025; Loosen et al. 2024; Smallwood and Schooler 2015). Furthermore, there is a lack of integrated models that can account for the multidimensional nature of mind wandering, including its cognitive, affective, and neural correlates (Shareef‐Trudeau et al. 2025; Kleselet al. 2021).

This systematic review addresses these gaps by evaluating the accuracy, strengths, and limitations of different methods for measuring mind wandering subtypes. By synthesizing findings from diverse methodologies—including self‐reports, neuroimaging, behavioral tasks, experience sampling, eye‐tracking, and physiological measures—this review aims to provide a comprehensive understanding of mind wandering and its measurement. The review is guided by the *MAMW framework*, a novel approach that integrates subjective and objective measures to capture the full spectrum of mind wandering.

### 1.3 Theoretical Foundations

The MAMW framework is grounded in key theoretical frameworks that have shaped the study of mind wandering. Smallwood and Schooler (2015) emphasize the role of mind wandering in self‐generated thought and its neural underpinnings, particularly the involvement of the DMN. Their work highlights how mind wandering reflects a shift from externally directed attention to internally focused cognition, driven by the DMN. Christoff et al. (2016) propose a dynamic framework that views mind wandering as spontaneous thought, emphasizing the interplay between the DMN and other brain networks, such as the dorsal attention network (DAN) and salience network (SN). This framework provides a neural basis for understanding the fluid and context‐dependent nature of mind wandering.

Building on these foundations, Seli et al. (2018) introduce a “family‐resemblances” model that categorizes mind wandering into subtypes based on features such as intentionality, content, and temporal orientation. For instance, mind wandering can be classified as spontaneous (internally driven) or deliberate (externally triggered), future‐oriented or past‐oriented, and adaptive (e.g., problem solving, creativity) or maladaptive (e.g., boredom, distraction). This model underscores the heterogeneity of mind wandering and provides a structured approach for studying its diverse manifestations. However, while this model offers a foundational categorization, it does not fully address the integration of diverse measurement methods, the dynamic nature of mind wandering, or its clinical applicability. To address these limitations and build upon existing frameworks, this review proposes a new integrative perspective—one that aligns with emerging multidimensional approaches to understanding mind wandering, the MAMW framework. This approach integrates diverse methodologies to provide a holistic understanding of mind wandering, balancing theoretical insights with practical measurement challenges (A comparison of frameworks is provided in Table 1).

**TABLE 1:** Comparative frameworks for understanding mind wandering.

Aspect	Smallwood and Schooler (2015)	Dynamic framework of thought (Christoff et al. 2016)	Seli et al. (2018) “Family‐resemblances” model	Multidimensional assessment of mind wandering (MAMW) framework
Primary focus	Highlights the role of the *default mode network (DMN)* in mind wandering as a shift from externally directed attention to internally focused cognition.	Explores the dynamic nature of spontaneous thought, including mind wandering, and its neural and cognitive underpinnings.	Categorizes mind wandering into *subtypes* based on features like intentionality, content, and temporal orientation.	Focuses on the *comprehensive assessment* of mind wandering by integrating *multiple measurement methods* for research and clinical applications.
Key components	Focuses on *neural underpinnings* of mind wandering, particularly the DMN.	1. Self‐report measures: Subjective experiences of mind wandering. 2. Behavioral measures: Task performance and attentional lapses. 3. Neural measures: Brain activity	Emphasizes *heterogeneity* of mind wandering with categories like *spontaneous/deliberate, future/past‐oriented, and adaptive/maladaptive*.	1. Self‐report measures: Subjective experiences. 2. Behavioral measures: Task performance and attentional lapses. 3. Physiological measures: Neural and autonomic responses
Theoretical basis	Neural basis of self‐generated thought processes related to mind wandering.	Emphasizes the continuum of thought processes, from spontaneous to constrained, and their neural correlates.	Provides a *structured approach* to study diverse forms of mind wandering.	Emphasizes the *dimensional nature* of mind wandering, ranging from *normal to maladaptive* patterns, and integrates methods for clinical and research use.
Measurement integration	Emphasizes integration of neural and behavioral methods for studying mind wandering.	Integrates self‐report, behavioral, and neural measures to study the dynamics of thought.	Highlights *intentionality, content, and orientation* as key dimensions for categorization but does not fully integrate measurement methods.	Integrates *self‐report, behavioral, and physiological measures* to provide a *holistic assessment* of mind wandering.
Clinical applications	Provides insights into how shifts in attention can impact mental health and cognitive performance.	Primarily theoretical, with implications for understanding psychiatric conditions (e.g., ADHD, depression).	Limited focus on clinical applications; emphasizes theoretical categorization.	Explicitly designed for *clinical applications and theoretical categorization*
Contextual flexibility	Focused primarily on *neural studies* in controlled experimental settings.	Focuses on *laboratory‐based research* with controlled tasks and neural imaging.	Largely theoretical with limited focus on contextual adaptability.	Designed for both *laboratory and real‐world settings*, emphasizing *ecological validity* and contextual adaptability.
Strengths	Highlights the *neural basis of mind wandering* as linked to the DMN.	Provides a robust theoretical foundation for understanding the neural and cognitive mechanisms of mind wandering.	*Categorizes* mind wandering systematically, offering a structured model for diverse manifestations.	Offers a *practical, integrative framework* for assessing mind wandering in both research and clinical settings.
Limitations	Does not address the *heterogeneity* or diverse manifestations of mind wandering.	Limited focus on clinical applications and real‐world ecological validity.	Limited integration of measurement methods and lack of focus on clinical or real‐world applications.	May require further validation of its integrative approach and *scalability* across diverse populations and settings.

A concise comparison of four key frameworks—Smallwood and Schooler (2015), Dynamic Framework of Thought (Christoff et al. 2016), Seli et al. (2018), and the Multidimensional Assessment of Mind Wandering (MAMW) Framework—highlighting their focus, components, theoretical basis, and applications.

### 1.4 Toward a Multidimensional Approach: The MAMW Framework

The MAMW framework has been developed as a conceptual model informed by the findings of this review. It advocates a multimodal approach that integrates subjective self‐report measures with objective physiological and behavioral assessments. By synthesizing diverse methodologies—including experience sampling methods (ESMs), neuroimaging techniques, eye‐tracking, and physiological recordings—the MAMW framework aims to capture both the internal, subjective experiences of mind wandering and its external, measurable manifestations. This integrative approach enhances the accuracy, reliability, and ecological validity of findings, providing a structured model for exploring the mechanisms, correlates, and consequences of mind wandering across contexts and populations.

By adopting this framework, researchers can transcend fragmented insights and move toward a multidimensional understanding of mind wandering, bridging the gap between theoretical characterization and practical measurement. The MAMW framework not only advances theoretical understanding but also offers practical applications in areas such as clinical assessments, educational interventions, and real‐world contexts.

The primary objective of this systematic review is to evaluate the accuracy, strengths, and limitations of methods for measuring mind wandering subtypes. A secondary objective is to propose the MAMW framework as an integrative approach to address the limitations of current methods.

## 2 Methods

### 2.1 Search Strategy

This systematic review followed the *PRISMA* (Preferred Reporting Items for Systematic Reviews and Meta‐Analyses) guidelines and utilized *Covidence*, a web‐based tool for streamlining the review process. Four databases—PsycINFO, PubMed, Frontiers in Psychology, and Web of Science—were searched for studies published from inception to 2024. The search strategy included the following keywords and Boolean operators: (“mind wandering” OR “mind‐wandering”) AND (“measurement” OR “assessment”); (“spontaneous thought” OR “self‐generated thought”) AND (“neural correlates” OR “cognitive processes”); (“default mode network” OR “DMN”) AND (“mind wandering” OR “attention lapses”); (“experience sampling” OR “thought probes”) AND (“ecological validity” OR “real‐time assessment”).

### 2.2 Selection Criteria

Studies were included if they met the following criteria: they involved an adult sample (ages 18–65) with at least 20 participants; were original empirical studies, including randomized controlled trials (RCTs), observational studies, cross‐sectional studies, prospective studies, and retrospective studies; were published in English; provided full‐text sources; contained sufficient information to calculate an effect size or allowed for qualitative synthesis; were peer‐reviewed and published; and were conducted from the inception of the available time period in the selected databases up to the search date (2024).

Studies were excluded for *methodological flaws* (e.g., inadequate sample size, lack of control groups, or poor study design) or *irrelevance* (e.g., studies focusing on populations outside the specified age range or clinical conditions unrelated to mind wandering). This distinction ensures transparency and objectivity in the selection process.

### 2.3 Study Selection Process and Data Extraction

Data extraction and study selection were performed independently by two reviewers using a standardized tool (Covidence nonintervention reviews extraction tool). A total of 4076 studies were identified through database searches. After removing 2543 duplicates automatically and 31 manually, 1502 studies underwent title and abstract screening. Two independent reviewers applied the inclusion and exclusion criteria, excluding 661 irrelevant studies. The remaining 841 studies were assessed for eligibility through full‐text review, with 286 studies excluded based on predefined criteria (e.g., methodological flaws, insufficient data). This resulted in 555 studies included for data extraction and synthesis.

The following data were collected from each study: study details (author, year, country, study design), sample characteristics (sample size, age range, demographic information), measurement methods (specific tools and techniques used), outcomes (key findings related to mind wandering subtypes), and quality assessment data (scores from quality assessment tools). Data synthesis was conducted using a thematic analysis approach, categorizing studies based on measurement methods and synthesizing findings to evaluate the strengths and limitations of each method.

**FIGURE 1:**
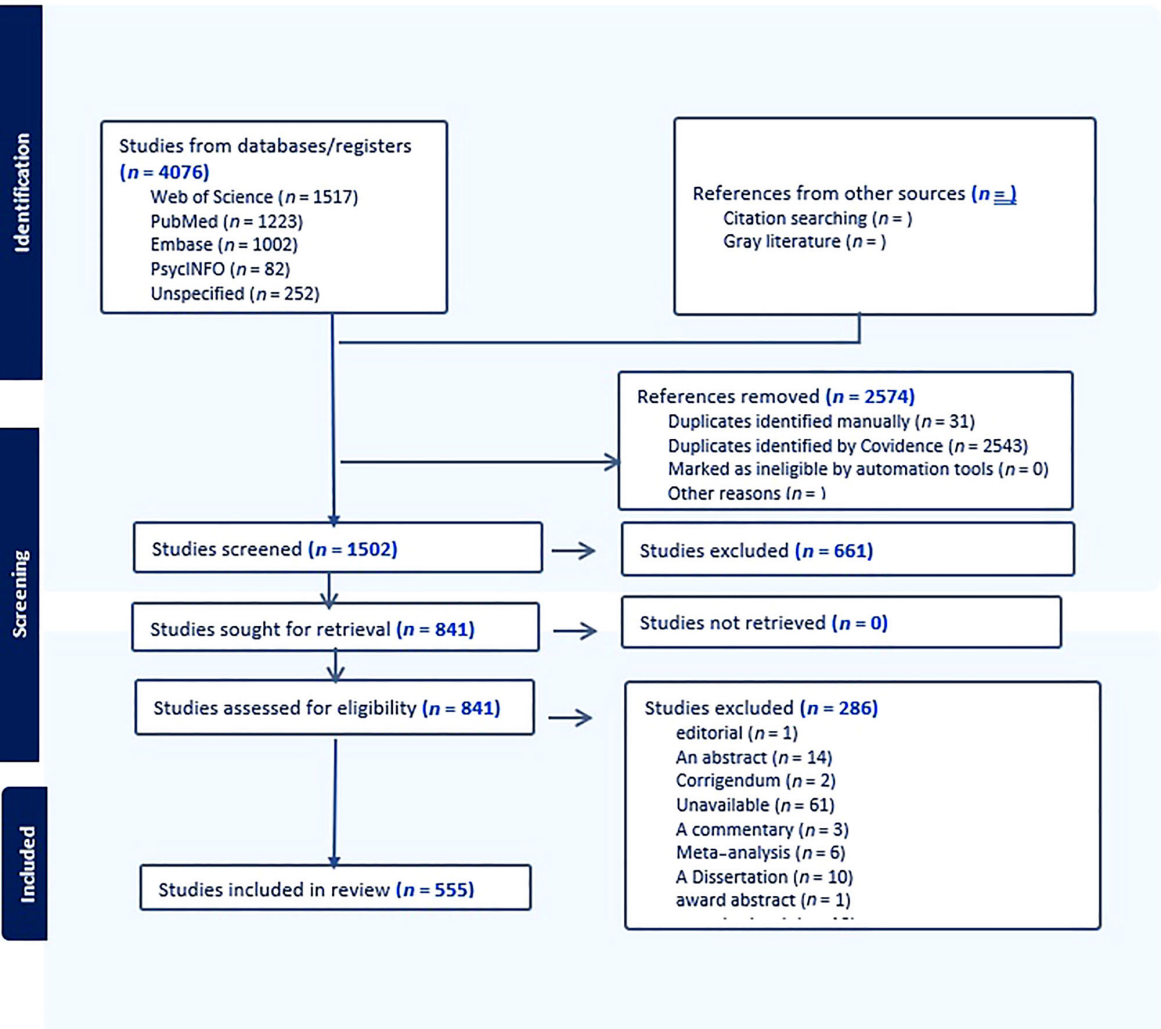
PRISMA flow diagram.

Inter‐rater reliability for study selection and data extraction was calculated using Cohen's kappa (*κ* = 0.85), indicating strong agreement. Disagreements were resolved through discussion and consensus, ensuring rigorous and unbiased study selection. The study selection process was documented using a PRISMA flow diagram, which enhances transparency and reproducibility (Figure 1).

### 2.4 Quality Assessment

The quality of included studies was assessed by defining specific criteria based on the focus of the review, including relevance, methodology, sample size, and bias. Relevance was determined by evaluating whether the study or data addressed the specific research question, while methodology was assessed to ensure that the methods used for data collection were reliable and appropriate. Sample size adequacy was also considered to determine the robustness of the findings, and potential conflicts of interest or biases in the sources were carefully identified. Two independent reviewers evaluated each study against these criteria, resolving disagreements through discussion and consensus. Based on their adherence to these criteria, studies were categorized as low, moderate, or high risk of bias. This thorough assessment ensured that the review's findings were grounded in high‐quality evidence and minimized the influence of biased or flawed studies.

## 3 Results

### 3.1 Study Characteristics

A total of *555 studies* were included in the final synthesis after the screening and eligibility process. These studies were conducted across diverse populations, with sample sizes ranging from 20 to over 1000 participants. The studies focused on adult populations aged *18–65*, with a mix of healthy individuals and clinical populations (e.g., ADHD, depression). The studies employed a variety of methodologies, including self‐reports, neuroimaging, behavioral tasks, ESMs, eye‐tracking, and physiological measures. A summary table of included studies is provided, detailing key characteristics such as sample size, measurement methods, and main findings. This table ensures transparency, allows for a comparative analysis of the studies, and provides a comprehensive overview that enables readers to compare and interpret the results effectively (Table 2).

**TABLE 2:** Included studies.

Authors, year, and title	Participants	Objective	Measure	Method
Wang et al. 2015; An investigation of the neural substraites of mind wandering induced by viewing traditional Chinese landscape painting.	Chinese college‐aged participants from Southwest University in Chongqing, China; 20 (11 female, nine male)	Investigate the effect of viewing traditional Chinese landscape paintings on mind wandering and cognitive control	Relaxation, Mind Wandering, Cognitive Control	Neuroimaging Techniques, Behavioral Performance Measures
Tusche et al. 2014; Classifying the wandering mind: revealing the affective content of thoughts during task‐free rest periods	Thirty healthy volunteers (15 female, aged between 21 and 33 years	Decode the valence of internally driven, self‐generated thoughts during task‐free rest based on neural similarities with task‐related affective mental states	Affective Content of Thoughts	Neuroimaging Techniques, Multivariate Pattern Analyses (MVPA)
Alfonso Morillas‐Romero et al. 2023; Spanish and cross‐cultural validation of the mind excessively wandering scale	391 Spanish and 713 British nonclinical individuals	Re‐examine the factor structure of MEWS, validate the Spanish version of MEWS, and conduct cross‐cultural validation of MEWS in Spanish and UK samples	Factor Structure of MEWS	Questionnaires
Diaz et al. 2014; The ARSQ 2.0 reveals age and personality effects on mind‐wandering experiences	Various participants from different demographics across multiple studies	Investigate the effects of age and personality on mind wandering experiences using ARSQ 2.0	Mind Wandering (ARSQ 2.0)	Questionnaires, Confirmatory Factor Analysis
Guesdon et al. 2020; Mind‐wandering changes in dysphoria	83 participants (57 women and 26 men), aged between 18 and 50 years	Evaluate MW and IAM in stable dysphoric participants compared to controls and compare their thought characteristics	Mind‐Wandering, Involuntary Autobiographical Memory (IAM)	Thought Probes, Questionnaires, Monotonous Vigilance Tasks
Chen et al. 2021; Social robots for evaluating attention state in older adults	50 participants (22 older adults, 28 younger adults)	Examine the effect of using a robotic experimenter on attention state in older adults	Attention State	Thought Probes, Questionnaires, Modified Sustained Attention to Response Task (SART)
Bortolla et al. 2021; Mindfulness effects on mind wandering and autonomic balance	28 female university students, aged around 24.44 years	Clarify the psychological, physiological, and affective impact of a mindfulness exercise on mind wandering	Mind Wandering, Heart Rate Variability (HRV)	Questionnaires, Cardiac Activity Assessment, Mindfulness Observing and Describing Questionnaire (MODQ), Amsterdam Resting State Questionnaire (ARSQ)
Hu et al. 2012; Different efficiencies of attentional orienting in different wandering minds	65 students (36 males, 29 females), aged between 20 and 29 years	Examine the relations between properties of attentional networks and Mind Wandering (MW)	Attention Networks, Mind Wandering (MW), Reaction Time (RT) Variability	Attention Network Test (ANT), Thought Probes, Sustained Attention to Response Task (SART)
Carriere et al. 2013; Wandering in both mind and body: individual differences in mind wandering and inattention predict fidgeting	304 participants (156 males, 148 females), aged between 18 and 72 years	Examine the relation between inattention, mind wandering, and fidgeting behavior	Inattention, Mind Wandering, Fidgeting	Questionnaires (MAAS‐LO, ARCES, MFS, SAQ), Online Study, Thought Probes
Bernstein et al. 2019; Utility of a novel simulator paradigm in the assessment of driving ability in individuals with and without attention‑deficit hyperactivity disorder	87 participants (16 with ADHD, 71 without)	Evaluate the utility of a cost‐effective driving simulator in detecting differences in driving performance between individuals with and without ADHD	Driving Performance, Attention, Executive Functioning	Driving Simulator (Assetto Corsa), Questionnaires, Computerized Tasks (n‐back, antisaccade, number‐letter switching, SART)
Cheyne et al. 2016; “You're on ten, where can you go from there?” Tufnel problems in repeated experiential judgments	219 undergraduate students from the University of Waterloo	Investigate the effects of using different point Likert scales to assess mind wandering during a sustained attention task	Depth of Mind Wandering	Likert Scales, Thought Probes, Metronome Response Task (MRT)
Vannucci et al. 2020; Distinguishing spontaneous from deliberate mind wandering in adolescents: the role of attentional control and depressive symptoms	439 adolescents (47% females; mean age: 14.65 years)	Investigate the associations of spontaneous and deliberate mind wandering with attentional control and depressive symptomatology in adolescents	Spontaneous and Deliberate Mind Wandering, Attentional Control, Depressive Symptoms	Questionnaires (MW‐S, MW‐D, AC: D, AC: S, YSR‐D)
Carciofo 2024; Circadian functioning and time perspectives: associations with eveningness, morning affect, and amplitude distinctness	299 Chinese university students (94 males, 205 females), aged between 18 and 25 years	Assess associations between time perspectives and components of circadian functioning, including eveningness, morning affect, and amplitude distinctness	Morning Affect, Eveningness, Distinctness, Time Perspective, Mind Wandering, Conscientiousness, Life Satisfaction, Positive and Negative Affect, Sleep Quality	Questionnaires (MESSi, ZTPI, BFI‐44, Mind Wandering Scales, PANAS, Students' Life Satisfaction Scale, Sleep Quality and Duration), Online Survey
Broadway et al. 2015; Early event‐related brain potentials and hemispheric asymmetries reveal mind‐wandering while reading and predict comprehension	47 undergraduate students (33 female), age M = 18.56 years, SE = 0.14	Examine the EEG of mind wandering through ERPs and prestimulus alpha, and how they predict reading comprehension	Mind Wandering, Event‐Related Potentials (ERPs), Pre‐Stimulus Alpha, Reading Comprehension	EEG/ERP Recording, Reading Passages, Thought Probes
Frank et al. 2015; Validating older adults’ reports of less mind‐wandering: an examination of eye movements and dispositional influences	36 young adults (ages 18–25) and 40 older adults (ages 60–85)	Test the validity of thought reports via eye‐tracking and examine whether mindfulness or positive mood protects older adults from task‐unrelated thoughts	Mind‐Wandering, Task‐Related Interference (TRI), Mood, Mindfulness	Eye‐Tracking, Reading Task, Thought Probes, Questionnaires (PANAS, FFMQ)
Maillet and Rajah 2013; Age‐related changes in frequency of mind‐wandering and task‐related interferences during memory encoding and their impact on retrieval	31 young adults (age range: 18–32, mean: 22.6, 22 women) and 26 older adults (age range: 60–76, mean: 64.30, 15 women)	Investigate the association between the frequency of mind wandering and task‐related interferences at encoding and retrieval accuracy in young and older adults	Mind‐Wandering, Task‐Related Interferences (TRI), Memory Retrieval	Encoding Tasks (Objective: Man‐made/Natural judgment, Subjective: Pleasant/Neutral judgment), Thought Probes, Memory Retrieval Tasks
Jin et al. 2019b; Predicting task‐general mind‐wandering with EEG	30 subjects (18 subjects included in analysis, 13 females, ages 18–30)	Develop a machine‐learning classifier to determine mind wandering state using EEG across different tasks	Mind‐Wandering, EEG Markers (P1, N1, P3, Theta and Alpha Power and Coherence)	EEG Recording, Machine‐Learning Classifier, Sustained Attention to Response Task (SART), Visual Search Task, Thought Probes
Kajimura et al. 2019; Challenge to unity: relationship between hemispheric asymmetry of the default mode network and mind wandering	27 healthy, right‐handed participants with no history of neurological or psychiatric disease	Investigate the hemispheric asymmetry of the Default Mode Network (DMN) and its relation to mind wandering using tDCS, effective connectivity estimation, and machine learning	Mind‐Wandering, Hemispheric Asymmetry, Effective Connectivity	Transcranial Direct Current Stimulation (tDCS), fMRI, Sustained Attention to Response Task (SART), Machine Learning
Zhou et al. 2020; Driver's distracted behavior: the contribution of compensatory beliefs increases with higher perceived risk	304 nonprofessional drivers (186 males, 118 females), aged between 19 and 66 years	Investigate the ability of drivers’ compensatory beliefs and risk perceptions to predict drivers’ distracting behaviors	Distracting Behaviors, Compensatory Beliefs, Perceived Risk	Questionnaires (Driver's Distractive Compensatory Beliefs (DDCB), Distracted Driving Behavior, Perceived Risk), Online Survey
Steindorf and Rummel 2019; Do your eyes give you away? A validation study of eye‐movement measures used as indicators for mindless reading	107 participants (Mage = 22.58, SDage = 4.01, 78.50% female)	Validate eye‐movement measures as indicators of mindless reading and examine the effect of metacognitive awareness on mind wandering behavior	Eye Movements, Reading Time, Fixation Count, First‐Fixation Duration, Word‐Frequency Effect	Eye‐Tracking, Reading Task, Thought Probes, Multilevel Modeling
Yang et al. 2021; The effects of posture on mind wandering	36 native Chinese‐speaking healthy college students, aged between 18 and 26 years	Explore whether body postures influence mind wandering using a reading comprehension task	Mind Wandering Frequency, Meta‐Awareness, Reading Performance	Reading Comprehension Task, Probe‐Caught Technique, Self‐Caught Method, Psychophysics Toolbox, MATLAB, E‐prime
Lux et al. 2023; When self comes to a wandering mind: brain representations and dynamics of self‐generated concepts in spontaneous thought	63 healthy, right‐handed participants (final analysis: 62 participants), age = 23.0 ± 2.5 years	Examine brain representations and dynamics of self‐generated concepts in spontaneous thought using fMRI	Self‐Generated Concepts, Conceptual Associations, Emotional Valence, Self‐Relevance	fMRI, Free Association‐Based Thought Sampling Task (FAST), Self‐Report Questionnaires, Multivariate Pattern‐Based Predictive Modeling
Unsworth and McMillan 2012; Mind wandering and reading comprehension: examining the roles of working memory capacity, interest, motivation, and topic experience	150 participants (63% female), ages 18–35 years	Examine the roles of working memory capacity, interest, motivation, and topic experience in mind wandering and reading comprehension	Mind Wandering, Reading Comprehension, Working Memory Capacity, Interest, Motivation, Topic Experience	Confirmatory Factor Analysis, Structural Equation Modeling, Working Memory Tasks (Ospan, Symspan, Rspan), Academic Text Reading, Thought Probes, Reading Comprehension Test, Questionnaire
Gilead et al. 2015; Neural activity associated with subsequent memory for stimulus‐evoked internal mentations	21 right‐handed participants (12 women), average age 24.7 years	Investigate the neurobiological basis of the ability to remember one's internal mentations	Internal Mentations, Subsequent Memory, Neural Activity	fMRI, Sentence Presentation, Reading Comprehension Questions, Button Press Responses
Turnbull et al. 2019; The ebb and flow of attention: between‐subject variation in intrinsic connectivity and cognition associated with the dynamics of ongoing experience	157 participants (95 females; mean age = 20.43 years)	Explore neurocognitive processes underpinning the dynamics of ongoing cognition and its variations across individuals	Attention, Intrinsic Connectivity, Cognition, Mind‐Wandering	fMRI, Experience Sampling, 0‐Back and 1‐Back Tasks, Multidimensional Experience Sampling (MDES), Principal Component Analysis (PCA)
Kawagoe et al. 2020; The association of motivation with mind wandering in trait and state levels	176 undergraduate students (18–24 years old; 68 male) for trait level investigation; 107 participants for state level investigation	Examine the association between trait‐state motivation and trait‐state mind wandering	Trait and State Motivation, Trait and State Mind Wandering	Questionnaires (Apathy Scale, Mind Wandering Questionnaire, Daydreaming Frequency Scale, Intrinsic Motivation Inventory), Sustained Attention to Response Task (SART), Experience Sampling
Moran et al. 2020; Young and restless, old and focused: age‐differences in mind‐wandering frequency and phenomenology	34 younger and 34 healthy older adults	Examine the impact of aging on the frequency and phenomenology of mind wandering and investigate variables mediating age‐related differences in unintentional and intentional mind wandering	Unintentional and Intentional Mind‐Wandering, Cognitive Function, Negative Affect, Task Engagement, Reaction Time Variability	Neuropsychological Test Battery, Contrast Change Detection Task with Experience Sampling Probes, Self‐Report Questionnaires (HADS, CAARS), Stanford Sleepiness Scale (SSS), Dundee Stress State Questionnaire (DSSQ)
Stawarczyk and D'Argembeau 2016; Conjoint influence of mind‐wandering and sleepiness on task performance	95 French‐speaking participants (66 women) from the Belgian general population	Investigate whether mind wandering and sleepiness have distinguishable effects on task performance	Mind‐Wandering Frequency, Sleep‐Related Disturbances, Task Performance	Sustained Attention to Response Task (SART) with Thought Probes, Karolinska Sleepiness Scale (KSS), Thought Characteristics Questionnaire (TCQ), Insomnia Severity Index (ISI), Daydreaming Frequency Scale (DDFS)
Vess et al. 2016; True self‐alienation positively predicts reports of mind wandering	93 introductory psychology students (51 females, 39 males), ages 18–38 years	Assess the relationship between feelings of true self‐alienation and self‐reported mind wandering during performance tasks	True Self‐Alienation, Mind wandering, Authenticity, Mindfulness, Self‐Concept Clarity, Affect, Personality Dimensions, Self‐Esteem, Meaning in Life	Questionnaires (Authentic Personality Scale, Cognitive and Affective Mindfulness Scale—Revised, Self‐Concept Clarity Scale, Positive and Negative Affect Schedule, Ten Item Personality Inventory, Single Item Self‐Esteem Scale, Meaning in Life Questionnaire), Sustained Attention to Response Task (SART)
Denkova et al. 2018; Attenuated face processing during mind wandering	36 undergraduate students (18 women), age range 18–25 years	Investigate the impact of mind wandering on behavioral and neural responses to faces	Mind Wandering, Face Processing, N170 Component, P1 Component, P3 Component	Sustained Attention to Response Task with Faces (F‐SART), EEG Data Acquisition, Continuous EEG Recording
Geffen et al. 2017; Reduced mind wandering in patients with Parkinson's disease	38 PD patients and 30 age‐matched controls with a MoCA score of 26 or above	Explore differential mind wandering capabilities in Parkinson's disease patients compared to healthy controls	Mind Wandering Frequency, Default Mode Network (DMN) Connectivity	Shape Expectations Task, Montreal Cognitive Assessment (MoCA), Unified Parkinson's Disease Rating Scale (MDS‐UPDRS), Levodopa Equivalent Daily Dose (LEDD), Beck Depression Inventory (BDI‐II)
Deng et al. 2014; The relationship between wandering mind, depression and mindfulness	23 healthy students (12 males, mean age 21.9) from Dalian University of Technology	Examine the relationship between wandering mind, depression, and mindfulness	Mind‐Wandering Frequency, Depression, Dispositional Mindfulness	Sustained Attention to Response Task (SART), Online Thought Probes, Beck Depression Inventory (BDI), Mindfulness Attention and Awareness Scale (MAAS)
Wilson et al. 2018; Prolonging the response movement inhibits the feed‐forward motor program in the sustained attention to response task	84 participants, ages 19–30 years (M = 21.4, SD = 1.8)	Investigate whether performance in the Sustained Attention to Response Task (SART) measures perceptual decoupling or response strategies and motor inhibition	Commission Errors, Omission Errors, Response Time (RT), Self‐Reported Mental Demand, Fatigue, Task‐Unrelated Thoughts	Sustained Attention to Response Task (SART), Self‐Report Questionnaires (Stress Scale, NASA–Task Load Index), Manual Movement vs. Automatic Conditions, Cursor Movement Tracking
Sikka et al. 2020; COVID‐19 on mind: daily worry about the coronavirus is linked to negative affect experienced during mind‐wandering and dreaming	172 participants for dream logs (25 men, 139 women, eight “other”; mean age 46.99) and 152 participants for mind wandering logs (23 men, 123 women, six “other”; mean age 48.42)	Investigate the association between COVID‐19‐related concern, anxiety, daily worry, and the affective quality of mind wandering and dreaming	Negative and Positive Affect, COVID‐19 Worry, Sleep Quality, Mind‐Wandering, Dreaming	Ecological Momentary Assessment, Daily Logs, Self‐Report Questionnaires
Lopez et al. 2023; The four factors of Mind Wandering Questionnaire: content, construct, and clinical validity	Content Validity: 32 psychologists and psychotherapists (Panel 1) and 60 master students (Panel 2); Construct Validity: 530 participants (265 females, ages 19–35)	Develop and validate the Four Factors of Mind Wandering (4FMW) Questionnaire	Failure in Social Interaction, Failure in Interaction with Objects, Unawareness, Inattention	Content Validity Assessment by Expert Panels, Exploratory Factor Analysis (EFA), Confirmatory Factor Analysis (CFA), Convergent Validity Test with MW Questionnaire of Borella
Kam et al. 2012; The four factors of Mind Wandering Questionnaire: content, construct, and clinical validity	22 participants (First Experiment), 15 participants (Second Experiment); all right handed with no history of neurological problems	Determine whether mind wandering episodes can be considered as periods of “response‐independent” thought using behavioral and ERP measures	Mind Wandering, Behavioral Performance, Feedback Error‐Related Negativity (fERN), Attention State	Visuomotor Tracking Task, Time‐Estimation Task, Event‐Related Potentials (ERP), Feedback Signals
Maillet et al. 2020; Differential effects of mind‐wandering and visual distraction on age‐related changes in neuro‐electric brain activity and variability	36 young adults (mean age 23) and 31 older adults (mean age 71.7)	Assess age‐related differences in event‐related potentials and neural variability associated with internal distraction and visual distractors	Mind‐Wandering, Visual Distraction, Event‐Related Potentials (ERP), Neural Variability, P3 Amplitude, Reaction Time (RT)	Go/No‐Go Task, EEG Recording, Face‐Scene Stimuli, Montreal Cognitive Assessment (MoCA)
Bocharov et al. 2018; EEG dynamics of spontaneous stimulus‐independent thoughts	30 healthy, right‐handed participants (14 men), aged 18–25 years (mean age 20.8)	Compare oscillatory dynamics accompanying self‐referential and non‐self‐referential stimulus‐independent thoughts	Self‐Referential and Non‐Self‐Referential Thoughts, Theta, Alpha, Beta Spectral Power	EEG Recording with 128 Electrodes, Independent Component Analysis, Dipole Localization, Quik‐Cap 128 NSL, Neuroscan (USA) Amplifier
Walker and Trick 2018; Mind‐wandering while driving: the impact of fatigue, task length, and sustained attention abilities	40 undergraduate students (31 female), mean age 18.7 years	Investigate the impact of task length and fatigue on mind wandering while driving and whether individual differences in sustained attention predict mind wandering	Mind‐Wandering Frequency, Driving Performance (Speed, Steering Variability, Hazard Response Time), Self‐Rated Difficulty in Focusing Attention	| Fixed Base Oktal Driving Simulator, Mind‐Wandering Probes, Sustained Attention to Response Task (SART), Post‐Drive Self‐Report, Dundee Stress State Questionnaire (DSSQ)
Gaynor and Fitzgerald 2023; Mind‐wandering and its relationship with psychological wellbeing and obsessive‐compulsive symptomatology in the context of Covid‐19	177 participants fully completed all measures	Explore mind wandering as a potential contributing factor to poor mental health in the context of the Covid‐19 pandemic	Mind‐Wandering Frequency, Type, and Content, Psychological Wellbeing, Obsessive‐Compulsive Symptomatology, Covid‐Related Stress	Online Questionnaire, Daydreaming Frequency Scale, Mind‐Wandering: Deliberate and Spontaneous Scales, Social Daydreaming Content Subscales, International Support Evaluation List, Covid Stress Scale
Li et al. 2023; Neural representations of self‐generated thought during think‐aloud fMRI	86 participants (45 females; mean age = 22.1)	Investigate the role of self‐generated thoughts in resting‐state fMRI using the think‐aloud method	Self‐Generated Thoughts, Brain Activation Patterns, Thought Content Divergence, Representational Similarity Analysis	Think‐Aloud fMRI Method, GE MR750 3.0T Scanner, T1‐Weighted Images, Echo‐Planar Imaging (EPI), Self‐Report Ratings, Fiber Optic Microphone
Biederman et al. 2017; Towards operationalising internal distractibility (mind wandering) in adults with ADHD	41 unmedicated adults ages 18–55 of both sexes	Investigate whether specific symptoms of ADHD can help identify ADHD patients with mind wandering	Mind Wandering, ADHD Symptoms (Inattention, Hyperactivity/Impulsivity)	Mind‐Wandering Questionnaire (MWQ), ADHD Module of the Schedule for Affective Disorders and Schizophrenia for School‐Age Children Epidemiologic Version (K‐SADS‐E), Spearman's rank correlation, Pearson's *χ* ^2^ analyses, receiver operating characteristic (ROC) analysis
Karapanagiotidis et al. 2017; Tracking thoughts: exploring the neural architecture of mental time travel during mind‐wandering	86 healthy participants (51 women, age range 18–31)	Identify the structural and functional brain organization that underlies individual differences in the capacity to engage in mental time travel (MTT)	Mental Time Travel (MTT), Functional and Structural Brain Organization	Functional MRI (fMRI), Diffusion Tractography Analysis, Probabilistic Connectivity Mapping, Choice Reaction Time Task, Multidimensional Experience Sampling (MDES)
Niedźwieńska and Kvavilashvili 2018; Reduced mind‐wandering in mild cognitive impairment: testing the spontaneous retrieval deficit hypothesis	23 participants with aMCI and 25 healthy controls	Test the Spontaneous Retrieval Deficit Hypothesis, which predicts that people with aMCI are particularly impaired on tasks that rely on spontaneous retrieval processes	Spontaneous Thoughts (Involuntary Memories, Current Thoughts, Future Thoughts), Mind‐Wandering Frequency	Vigilance Task, Thought Probes, Hopkins Verbal Learning Test—Revised (HVLT‐R), Wechsler Memory Scale–third Edition, Verbal Fluency Test, Trail Making Test (TMT), Geriatric Depression Scale 30 (GDS30)
Gross et al. 2021; Comparing the phenomenological qualities of stimulus‐independent thought, stimulus‐dependent thought and dreams using experience sampling	131 undergraduate students (81 females, mean age 19.3)	Compare the content of two types of stimulus‐independent thought (dreaming and waking SITs) with stimulus‐dependent thoughts	Thought Appraisals (Novelty, Fluidity, Meaningfulness, Continuity, Goal‐Directedness, Bizarreness, Spontaneity, Emotionality, Emotional Valence, Temporal Orientation)	Smartphone‐Based Experience‐Sampling Method (ESM), MetricWire App, Pseudo‐Random Probe Delivery, Day and Night Triggers
Kruger et al. 2020; Using deliberate mind‐wandering to escape negative mood states: implications for gambling to escape	111 participants (56 female and 55 male), ages 23–92 (mean age 59.25)	Investigate whether gamblers use deliberate mind wandering as a maladaptive means to regulate affect during repetitive tasks	Mind‐Wandering Frequency (On‐Task, Spontaneous, Deliberate), Negative and Positive Affect, Gambling Severity, Mindfulness	Slot Machine Simulator, Auditory Vigilance Task, Thought Probes, Depression, Anxiety, and Stress Scale (DASS‐21), Boredom Proneness Scale (SBPS), Canadian Problem Gambling Index (CPGI), Problem Gambling Severity Index (PGSI), Mindful Attention Awareness Scale (MAAS), Game Experience Questionnaire (GEQ)
Martinon et al. 2022; Catching thoughts: self‐caught experience sampling preferentially captures characteristic features of off‐task experiences across the life span	36 young adults (M = 21.94, SD = 4.49; women = 29) and 38 older adults (M = 69.42, SD = 7.42; women = 24)	Compare mind wandering content when noticed by the participant (self‐caught) against those reported after externally initiated probes (probe‐caught)	Mind‐Wandering Content (Task‐Relevance, Temporal Focus, Self‐Referential), Off‐Task Characteristics, Self‐Caught and Probe‐Caught Reports	Vigilance Task (0‐Back Procedure), Multi‐Dimensional Experience Sampling (MDES), PsychoPy, On‐Screen Prompts, Likert Scale Ratings, Thought Probes, Self‐Caught Reports
Kane et al. 2017; For whom the mind wanders, and when, varies across laboratory and daily‐life settings	274 undergraduates (188 female, 81 male, five unreported gender), ages 18–35 years	Investigate the differences in mind wandering across laboratory and daily‐life settings	Mind‐Wandering Frequency, Executive‐Control Abilities (WMC, Attention Restraint, Attention Constraint), Personality Traits	Laboratory Cognitive Measures (Complex Span Tasks, Running Span, Updating Counters), Thought Probes during Laboratory Tasks, Daily‐Life Experience‐Sampling Study, Electronic Device Prompts, Personality Questionnaires
Philippi et al. 2021; Lesion network mapping demonstrates that mind‐wandering is associated with the default mode network	29 participants with brain injury and 19 healthy comparison participants	Test the hypothesis that lesions affecting the DMN and FPN would be associated with diminished mind wandering	Mind‐Wandering Frequency, Lesion Network Mapping, DMN and FPN Connectivity	Structural MRI Scanning, Computerized Axial Tomography (CT), Lesion Mapping, Lesion Network Mapping Analysis, Imaginal Processes Inventory (IPI), Neuropsychological Variables (Processing Speed, Working Memory, Verbal Memory, Language, Mood)
Rodriguez‐Larios et al. 2021; The EEG spectral properties of meditation and mind wandering differ between experienced meditators and novices	58 participants (29 with meditation experience and 29 without)	Assess whether differences in the subjective experience of meditation between meditators and nonmeditators are reflected in EEG spectral modulations	Focus Level, Mind‐Wandering Frequency, EEG Spectral Modulations	EEG Recordings with Nexus‐32 System, BioTrace Software, 19‐Electrode Cap, Vertical (VEOG) and Horizontal (HEOG) Eye Movements, Rest Condition, Meditation Condition, Meditation with Probe‐Caught Experience Sampling, Self‐Report Questions (Breath Focus, Distractions, Confidence, Drowsiness, Engagement)
Szpunar et al. 2013; Interpolated memory tests reduce mind wandering and improve learning of online lectures	Experiment 1: 32 students; Experiment 2: 48 students	Investigate whether interpolating online lectures with memory tests helps sustain attention, reduce mind wandering, and improve learning	Mind‐Wandering Frequency, Note Taking, Learning Improvement, Test Anxiety, Cognitive Demand	Lecture Videos, Interpolated Memory Tests, Arithmetic Problems, Thought‐Sampling Probes, Self‐Report Ratings, Final Cumulative Test
Kucyi et al. 2013; Mind wandering away from pain dynamically engages antinociceptive and default mode brain networks	51 healthy right‐handed adults (26 female, 25 male; mean age 25.02)	Assess behavioral and neural aspects of spontaneous disengagement of attention from pain	Mind‐Wandering Frequency, Pain Rating, Cognitive Interference, Functional Connectivity, Structural Connectivity	Transcutaneous Electrical Nerve Stimulation (TENS), Experience Sampling Task, Cognitive Interference Task, Functional Magnetic Resonance Imaging (fMRI), Dynamic Resting State Activity, Diffusion MRI, Thought Probes, Self‐Report Questions
Thiemann et al. 2022; Differential relationships between thought dimensions and momentary affect in daily life	113 participants (91 female, 17 male, five others), mean age 19.93	Examine the relationship between different thought dimensions (task‐relatedness, intentionality, freedom of movement) and momentary affect in daily life	Task‐Relatedness, Intentionality, Freedom of Movement, Momentary Affect, Thought Dimensions	Ecological Momentary Assessments (EMA), Qualtrics Platform, Randomized Email Notifications, Thought Dimensions Ratings, Affective Valence Ratings, Salient Event Reports
Seli et al.; On the relation of mind wandering and ADHD symptomatology	Two nonclinical samples: 1354 participants each; Clinical ADHD sample: 69 participants	Test the hypothesis that symptoms of ADHD are associated with spontaneous but not deliberate mind wandering	Deliberate and Spontaneous Mind Wandering, ADHD Symptoms	Mind Wandering: Deliberate (MW‐D) Scale, Mind Wandering: Spontaneous (MW‐S) Scale, Adult ADHD Self‐Report Scale v1.1 (ASRS), Likert Scales, Self‐Report Questionnaires, Comparison between Clinical ADHD Sample and Control Group
Jha et al. 2015; Minds “at attention”: mindfulness training curbs attentional lapses in military cohorts	M8D: 40 soldiers (age M = 25.8, SD = 4.5), M8T: 40 soldiers (age M = 26.7, SD = 6.2), NTC: 24 soldiers (age M = 27, SD = 6.1), CIV: 45 civilians (age M = 20.44, SD = 3.77)	Investigate if mindfulness training (MT) mitigates attentional performance lapses associated with task‐unrelated thought during high‐demand intervals	Attentional Performance (A’ Sensitivity Index), Mind‐Wandering Frequency, Errors of Commission, Response Time (RT), Intraindividual Coefficient of Variation (ICV), Subjective Ratings of Mind‐Wandering	Sustained Attention to Response Task (SART), Mindfulness‐based Mind Fitness Training (MMFT), Training‐Focused MT, Didactic‐Focused MT, No‐Training Control, Self‐Report Questions, Group‐Randomized Assignment
Lux et al. 2023; When self comes to a wandering mind: brain representations and dynamics of self‐generated concepts in spontaneous thought	| 63 healthy, right‐handed participants [age = 23.0 ± 2.5 years (means ± SD), 30 females]	Examine brain representations and dynamics of self‐generated concepts in spontaneous thought using free association–based thought sampling task	Self‐Generated Concepts, Concept Associations, Brain Representations, Affective Traits, Mental Health States	Functional Magnetic Resonance Imaging (fMRI), Free Association‐Based Thought Sampling Task (FAST), Concept Generation, Concept Reflection, Postscan Survey, Self‐Report Questionnaires (PANAS, CES‐D, RRS, STAI‐T, MASQ‐D30)
Kawagoe 2022; Executive failure hypothesis explains the trait‐level association between motivation and mind wandering	Study 1: 587 participants (mean age = 47.3 ± 13.5 years; 301 women); Study 2: 562 participants (mean age = 45.4 ± 13.7 years; 263 women)	Investigate the association between motivation and mind wandering at the trait level and determine its possible mechanisms	Motivation, Apathy, Mind Wandering, Executive Function	Online Survey, Apathy Scale (AS), Dimensional Apathy Scale (DAS), Mind Wandering Questionnaire (MWQ), Mind Wandering: Deliberate (MW‐D) and Spontaneous (MW‐S) Scales, Effortful Control Scale (ECS)
Irrmischer et al. 2018; Negative mood and mind wandering increase long‐range temporal correlations in attention fluctuations	Experiment 1: 62 participants (mean age = 25, 32 Female); Experiment 2: 89 participants (mean age = 22.4, 64 Female); Experiment 3: 35 participants (mean age = 21.3, 25 Female)	Investigate the impact of mood and mind wandering on trial‐to‐trial variability in performance during sustained attention tasks	Reaction Times, Attention Fluctuations, Mind Wandering, Mood Induction	Continuous Temporal Expectancy Task (CTET), Mood Manipulation, Externally Cued Mind Wandering Task, Probe‐Caught Reports, Reaction Time Measurements, Naturalistic Pictures, Thought Probes, Likert Scale Ratings
Pelagatti et al. 2018; Tracking the dynamics of mind wandering: insights from pupillometry	50 undergraduate students from the University of Florence (41 females, age range 18–27, M = 20.84 years)	Examine the dynamics of mind wandering using pupillometry during a vigilance task	Pupil Dilation, Mind‐Wandering Triggers, Vigilance Task Performance, Pupil‐Constriction Response	Pupillometry, Vigilance Task with Task‐Irrelevant Verbal Cues, Thought Probes, PsychoPhysics Toolbox for MATLAB, Eye Tracking with CRS LiveTrack System, White Horizontal and Vertical Line Patterns, Verbal Cues with Different Emotional Content, Self‐Report Questions
Wilson et al. 2016; Go‑stimuli proportion influences response strategy in a sustained attention to response task	30 undergraduate students from the University of Canterbury (12 males and 18 females), aged 20–54 years (M = 26.5, SD = 7.8)	Investigate how varying the proportion of Go stimuli affects response strategy in a sustained attention to response task (SART)	Reaction Times, Commission Errors, Task‐Related Thoughts, Task‐Unrelated Thoughts	Modified Go/No‐Go Tasks (with Go‐Stimuli Proportions of 0.50, 0.65, 0.80, and 0.95), Dundee Stress State Questionnaire (DSSQ), E‐Prime 2.0 Software, Images of Robots as Go and No‐Go Stimuli, Pre‐Task and Post‐Task Thought Measures, Practice Task, Verbal Accuracy Feedback
Feng et al. 2024; Mind wandering while reading easy and difficult texts	80 undergraduate students from a large US university	Examine the relation between mind wandering and task difficulty in reading comprehension of standardized texts	Mind‐Wandering Frequency, Reading Comprehension, Task Difficulty	Nelson–Denny Reading Comprehension Test, Modified Easy and Difficult Texts, Probe‐Caught Method for Mind Wandering, Multiple‐Choice Comprehension Questions, Flesch–Kincaid Grade‐Level Scores, Random Assignment of Passage Difficulties, Thought Probes at Key Text Positions
Axelrod et al. 2015; Increasing propensity to mind‐wander with transcranial direct current stimulation	Experiment 1: 14 participants (average age = 24.4 years, 8 male); Experiment 2: 31 participants (10 prefrontal cortex stimulation, 10 sham stimulation, 11 occipital cortex stimulation)	Investigate whether mind wandering can be modulated externally using brain stimulation (tDCS)	Mind‐Wandering Propensity, Task‐Unrelated Thoughts (TUTs), External Task Performance, Response Time	Within‐Subjects Design (Experiment 1), Between‐Subjects Design (Experiment 2), Transcranial Direct Current Stimulation (tDCS), Sustained Attention to Response Task (SART), Prefrontal Cortex Stimulation, Sham Stimulation, Occipital Cortex Stimulation, Thought Probes, MATLAB with Psychtoolbox, Written Informed Consent
Zedelius et al. 2015; Motivating meta‐awareness of mind wandering: a way to catch the mind in flight?	136 university students (76% women; average age 20.64 years, SD = 4.56)	Investigate whether motivating people to catch task‐unrelated thoughts (TUTs) increases meta‐awareness	Self‐Caught and Probe‐Caught Mind Wandering, Reading Comprehension, Failure to Detect Gibberish	One‐Factorial Between‐Subjects Design, Bogus Pipeline Procedure, Incentives for Self‐Catching, Gibberish Detection Task, Eye‐Tracking Simulation, Self‐Paced Reading, Semantic Violation Detection, Funneled Debriefing, Likert Scale Ratings
Lee et al. 2021; When eyes wander around: mind‐wandering as revealed by eye movement analysis with hidden Markov models	31 healthy adults (mean age = 22.77 years, SD = 2.87 years, 18 females)	Examine if eye movement patterns can differentiate between states of focused attention and mind wandering	Eye Movement Patterns (Centralized vs. Distributed), Target Detection Performance, Self‐Reported Attention States	Wearable Eye‐Tracker (Tobii Pro Glasses 2), Eye Movement Analysis with Hidden Markov Models (EMHMM), Sustained Attention to Response Task (SART), Go/No‐Go Task, Thought Probes, Self‐Report Ratings, Program E‐prime, Probe‐Caught Reports, Reward System for Participation
Krakau et al. 2022; Reduced past‐oriented mind wandering in left compared to right medial temporal lobe epilepsy	89 inpatients, grouped into six diagnostic subgroups: left MTL epilepsy (*n* = 20), right MTL epilepsy (*n* = 11), extratemporal epilepsy (*n* = 20), idiopathic epilepsies (*n* = 11), dissociative nonepileptic seizures (*n* = 12), and	Investigate the role of the left medial temporal lobe (MTL) in mind wandering, particularly toward the past, in patients with epilepsy	Mind‐Wandering Propensity, Temporal Orientation (Past/Present/Future), Meta‐Awareness, Self‐Rating Scales, Neuropsychological Domains	Sustained Attention to Response Task (SART) with Embedded Experience Sampling Probes, Self‐Rating Questionnaires (MWQ, ASRSv1.1, Beck Depression Inventory II, STAI‐Trait), Pathology and Neuropsychological Data, Clinical Parameters, Randomized Probe Intervals, Written Informed Consent,
	absences caused by cardiac syncopes (*n* = 15)			Compliance with the Declaration of Helsinki
Anderson and Farb 2020; The metronome counting task for measuring meta‐awareness	74 undergraduate students from the University of Toronto (mean age: 19, SD: 0.72, 23 male)	Develop a tool to dynamically measure meta‐awareness and track the loss of meta‐awareness	Self‐Caught Meta‐Awareness, Mind‐Wandering Depth, Task Engagement, Response Variability	Metronome Counting Task (MCT), Continuous Performance Task, Arrow‐Key Tapping, Beat Counting, Thought Probes, Self‐Rated Performance, Disengagement and Exclusion Criteria, Control Questions, Motivation Rating, Additional Questions for Exploratory Purposes, Written Informed Consent, Compliance with the Declaration of Helsinki
Meier 2022; Can research participants comment authoritatively on the validity of their self‐reports of mind wandering and task engagement? A replication and extension of Seli et al. (2015)	304 undergraduates from Western Carolina University (63% female, mean age 19)	Replicate the findings of Seli et al. and investigate potential individual differences in thought monitoring	Confidence Judgments, Thought‐Report Validity, Response Time Variability, Working Memory Capacity, Conscientiousness, Neuroticism, Dispositional Mindfulness	Metronome Response Task (MRT), Thought Probes, 5‐Point Likert Scale, Complex Span Tasks (Operation Span, Symmetry Span), NEO‐FFI‐3, Five Facet Mindfulness Questionnaire—Short Form (FFMQ‐SH), Course Credit as Compensation, Stopping Rule for Data Collection, Demographic Data, Informed Consent
Ju and Lien 2018; Who is prone to wander and when? Examining an integrative effect of working memory capacity and mindfulness trait on mind wandering under different task loads	160 participants (mean age = 20.3 years, SD = 2 years)	Investigate the relationship between working memory capacity (WMC), mindfulness, and mind wandering under different task loads	Mind‐Wandering Proportion, Mind‐Wandering Types (Aware vs. Unaware; Intentional vs. Unintentional), Task Load, Working Memory Capacity, Mindfulness Tendency	Within‐Participant Design, Modified 0‐Back and 2‐Back Tasks (Low and High Task Load), Self‐Caught Mind Wanderings, Thought Probes, Chinese Version of Mindfulness Awareness and Attention Scale (C‐MAAS), Chinese Version of Operation Span Task (OSPAN), Counterbalanced Task Order, Informed Consent, Compliance with Research Ethics Office of National Taiwan University
Spronken et al. 2016; Temporal focus, temporal distance, and mind‐wandering valence: results from an experience sampling and an experimental study	Study 1: 207 participants (100 women, 61 men, average age 39.6 years); Study 2: 79 participants (55 females, average age 21.97 years)	Investigate the relationship between temporal focus, temporal distance, and thought valence during mind wandering	Thought Valence, Temporal Focus (Past vs. Future), Temporal Distance (Near vs. Distant)	Experience Sampling Method, Smartphone Application, Six Signals per Day, Short Questionnaire (Happiness, Thoughts, Activities), Mind‐Wandering Questions, Temporal Focus and Distance Questions, Thought Valence Rating, Lab Experiment with Four Blocks (Past Week, Future Week, Past Year, Future Year), Counterbalanced Design, Recruitment via Various Channels, Compensation for Participation
Torres‐Irribarra et al. 2019; Positive and negative mind wandering: an assessment of their relationship with mindfulness and metacognition in university students	Study 1: 305 students (average age 20.5; 208 female); Study 2: 200 students (average age 21.9; 135 female)	Create a mind wandering scale in Spanish and investigate the relationship between positive and negative mind wandering, mindfulness, metacognition, and daydreaming frequency	Positive and Negative Mind Wandering, Mindfulness Facets, Metacognition Scales, Daydreaming Frequency, Academic Self‐Efficacy	Mind Wandering Scale (EDMVE), Metacognitive Strategies Scale, Five Facet Mindfulness Questionnaire (FFMQ), Goal Orientation and Learning Strategies Survey, Daydreaming Frequency Scale, Motivated Strategies for Learning Questionnaire (MSLQ), Correlation and Regression Analysis, Snack and Cinema Ticket Compensation
Zanesco et al. 2020; Self‐reported mind wandering and response time variability differentiate prestimulus electroencephalogram microstate dynamics during a sustained attention task	36 undergraduate students (18 women, Mage = 18.83 years, SDage = 1.28, age range = 18–25 years)	Investigate the association between electroencephalogram (EEG) microstate temporal dynamics and self‐reported mind wandering	Mind‐Wandering Reports, EEG Microstate Dynamics, Response Time Variability	Sustained Attention to Response Task (SART) with Faces, Experience Sampling Probes, Broadband EEG Recording, Topographic Segmentation, Microstate Clustering, Prestimulus Epoch Analysis, Data‐Driven Clustering of Topographic Voltage Patterns, Cartool Software, BioSemi ActiveTwo System, Written Informed Consent, Compliance with Institutional Review Board of the University of Miami
Gibb et al. 2022; Does mindfulness reduce negative interpretation bias?	Study 1: 135 undergraduate psychology students (75.6% female, Mage = 19.79 years, SD = 2.57); Study 2: 173 undergraduate psychology students (79.8% female, Mage = 19.6 years, SD = 2.71)	Investigate the association between mindfulness and negative interpretation bias, and test whether a mindfulness induction reduces negative interpretation bias	Negative Interpretation Bias, Trait Mindfulness, Psychological Distress, Negative Mood	Study 1: Cross‐Sectional Design, Interpretation Questionnaire (IQ), Mindful Attention Awareness Scale (MAAS), Cognitive and Affective Mindfulness Scale (CAMS‐R), Five‐Facet Mindfulness Questionnaire (FFMQ), Beck Depression Inventory (BDI‐II), Beck Anxiety Inventory (BAI), Perceived Stress Scale (PSS), Ruminative Response Scale (RRS); Study 2: Experimental Design, Mindfulness Induction (Audio Recording), Mind‐Wandering Task, Positive and Negative Affect Schedule (PANAS), Adolescents’ Interpretation and Belief Questionnaire (AIBQ), Post‐Induction Mood Assessment, Informed Consent, Course Credit as Compensation
Bedi et al. 2022; Go‐stimuli probability influences response bias in the sustained attention to response task: a signal detection theory perspective	406 introductory psychology students (289 female, ages 17–49, M = 19.8 years)	Explore the cause of commission errors in the sustained attention to response task (SART) based on Signal Detection Theory	Reaction Times, Omission Errors, Commission Errors, Response Bias	Random Assignment to Ten SART Conditions, E‐Prime 2.0 Software, Practice Trials with Visual Feedback, Data Analysis for Response Bias and Errors
Yan et al. 2009; Spontaneous brain activity in the default mode network is sensitive to different resting‐state conditions with limited cognitive load	20 participants (10 males, age range 18–24, mean age 21.06 ± 1.82 years)	Investigate the effect of different resting‐state conditions on the default mode network (DMN) using fMRI	Functional Connectivity, Regional Amplitude of Low Frequency Fluctuation (ALFF), Resting‐State Conditions (Eyes‐Closed, Eyes‐Open, Eyes‐Open with Fixation), Order Effect	Four Resting‐State fMRI Sessions (EC, EC, EO, EO‐F), Counterbalanced Sessions, SIEMENS TRIO 3‐Tesla Scanner, Echo‐Planar Imaging Sequence, Functional Images, T1‐Weighted Sagittal 3D Magnetization‐Prepared Rapid Gradient Echo (MPRAGE) Sequence, Midline Regions of Interest (ROIs), Data Analysis for Functional Connectivity and ALFF, Institutional Review Board Approval, Written Informed Consent
He et al. 2021; Pleasantness of mind wandering is positively associated with focus back effort in daily life: evidence from resting state fMRI	69 right‐handed, healthy undergraduates (12 males, age range 18–20, mean age 19.3 years); 58 participants included in final analysis	Investigate the relationship between the pleasantness of mind wandering, focus back effort, and functional connectivity in the brain	Mind Wandering Frequency, Focus Back Effort, Pleasantness of Mind Wandering Content, Functional Connectivity (rsfMRI)	Experience‐Sampling Methodology, Daily Questionnaires via WeChat, Seven‐Point Likert Scales, Resting‐State fMRI (3 T Siemens Trio MRI Scanner), Gradient Echo Planar Imaging (EPI) Sequences, High‐Resolution T1‐Weighted Anatomical Images (MPRAGE), Data Processing using DPARSF Toolbox in MATLAB, Correlation and Regression Analysis, Informed Consent, Compliance with
				Declaration of Helsinki, Institutional Approval
Nayda and Takarangi 2021; The cost of being absent: is meta‐awareness of mind‐wandering related to depression symptom severity, rumination tendencies and trauma intrusions?	200 participants (72.5% female, mean age 24.81 years, SD = 9.00)	Investigate the relationship between meta‐awareness of mind wandering, depression symptom severity, rumination tendencies, and trauma intrusions	Depression Symptom Severity, Dispositional Mindfulness, Mind‐Wandering with/without Meta‐Awareness, SART Target‐Error Rates, Trait Rumination Subtypes, Trauma Intrusions	Mindfulness Attention and Awareness Scale (MAAS), Beck Depression Inventory II (BDI II), Ruminative Responses Scale (RRS), Post‐traumatic Checklist 5 (PCL‐5), Sustained Attention to Response Task (SART) with Thought Probes, Correlation and Mediation Analysis, Counterbalanced Measures, Recruitment from University Student Pool and Community, Credit or Payment for Participation, Informed Consent, Preregistration and Data Amendment on Open Science Framework
Hao et al. 2015; More mind wandering, fewer original ideas: be not distracted during creative idea generation	87 healthy college students (12 males, 75 females, age range 18–25 years, M = 21.16, SD = 2.13)	Examine the effects of mind wandering during creative idea generation on fluency and originality scores	Mind Wandering Frequency, Creative Idea Generation (Fluency, Originality), Daydreaming Frequency, Thinking States, Task‐Related Interferences (TRIs)	Alternative Uses Task (AUT), Between‐Subject Design, Thought Probes (12 Probes in 20 min), Daydreaming Frequency Subscale (IPI), Dundee Stress State Questionnaire (DSSQ), Control and Experimental Conditions, Computer‐Based Recording of Ideas, ANOVA, Informed Consent, Compensation (5 USD), Institutional Ethics Committee Approval
Whitmoyer et al. 2019; Mindfulness training and attentional control in older adults: a randomized controlled trial	74 community‐dwelling older adults (randomly assigned to MBAT or active control group)	Investigate the effects of mindfulness‐based attention training (MBAT) on attentional control in older adults relative to an active control group	Attentional Control, Mind‐Wandering (Task‐Unrelated Thoughts, Task‐Related Interference), Trait and State Mindfulness, Positive and Negative Affect	Randomized Controlled Trial, Mindfulness‐Based Attention Training (MBAT), Active Control Group (Lifestyle Education), Pre‐ and Post‐Intervention Assessments, Continuous Performance Task (CPT), Modified Go/No‐Go Task, Mind‐Wandering Thought Probes, Mindful Attention Awareness Scale (MAAS), Homework Logs, Working Memory Index (WAIS‐IV), Data Analysis for Attentional Performance and Mind‐Wandering, CONSORT Diagram, Informed Consent, Random Assignment
Helfer et al. 2021; Emotion recognition and mind wandering in adults with attention deficit hyperactivity disorder or autism spectrum disorder	103 adults (43 with ADHD and 14 with ASD) diagnosed according to DSM‐5 criteria	Investigate the transdiagnostic traits of emotion recognition and mind wandering in adults with ADHD or ASD	Emotion Recognition (Facial Expression Identification), Mind Wandering (MEWS), Processing Speed, Emotional Dysregulation, Functional Impairment	Diagnostic Interview for Adult ADHD (DIVA), Autism Diagnostic Observation Schedule 2 (ADOS‐2), Autism Diagnostic Interview‐Revised (ADI‐R), Clinical Symptom Severity Questionnaires, Informant Data, Consultant Psychiatrist Evaluation, Barkley Adult ADHD Rating Scale (BAARS), Barkley Functional Impairment Scale, Social Responsiveness Scale—Adult Version (SRS‐2), Wender‐Reimherr Adult ADHD Scale (WRAAS), Wechsler Abbreviated Scale of Intelligence (WASI‐II), Informed Consent, Ethical Approval
Smith et al. 2023; Examining the relation between mind wandering and unhealthy eating behaviours	2328 participants recruited through the University Waterloo's participant pool (REG)	Explore how individual differences in mind wandering are related to unhealthy eating behaviors	Spontaneous Mind‐Wandering (MW‐S), Deliberate Mind‐Wandering (MW‐D), Self‐Control, Unhealthy Eating Behaviors,	Spontaneous (MW‐S) and Deliberate (MW‐D) Mind‐Wandering Scales, Brief Self‐Control Scale, Binge Eating Scale, “Starting the Conversation” (STC) Dietary Assessment, Eating‐Disorders Diagnostic Scale
			Eating‐Disorder Symptoms	(EDDS), Regression Analyses, Large Nonclinical Sample, Data Collection through Online Psychological Questionnaires, Partial Course Credit Compensation
Mills et al. 2018; Is an off‐task mind a freely‐moving mind? Examining the relationship between different dimensions of thought	228 participants enrolled at a large public Canadian university (*N* = 165 for analysis)	Test the relationship between freedom of movement in thought, task‐relatedness, and perceptual decoupling	Freedom of Movement in Thought, Task‐Relatedness, Perceptual Decoupling	Everyday Life Experience Sampling, Thought Probes Delivered via Mobile Phones, Ten Probes Per Day for 10 Days, Three Key Questions Rated on a Scale (1–7), Data Analysis for Intra‐Individual Correlations, Class Credit Compensation, Power Analysis for Sample Size Determination
Girardeau et al. 2022; Where is my mind …? The link between mind wandering and prospective memory	226 participants (mean age 37.85 ± 12.88; 180 female, 45 male, one other)	Test the link between mind wandering (MW) and the ability to perform prospective memory (PM) intentions	Mind Wandering (MW), Prospective Memory (PM) Abilities, Strategy Use, Depressive and Anxious Traits	Experience‐Sampling Probes via Mobile Phone, Mind Wandering Assessment (Phases I and III), Prospective Memory Assessment (Phase II), Short Version of the Metacognitive Prospective Memory Inventory (MPMI‐s), Mind Wandering Questionnaire (MWQ), Patient Health Questionnaire‐4 (PHQ‐4), Recruitment via Social Networks, LimeSurvey and Sendinblue for Survey Administration, Informed Consent, Ethical Approval by Université Paris Cité Research Ethics Committee
Lian et al. 2022; How and for whom is mobile phone addiction associated with mind wandering: the mediating role of fatigue and moderating role of rumination	1811 college students (63.34% female, average age 19.74, SD = 1.295) recruited from three universities in China	Investigate the relationship between mobile phone addiction and mind wandering, and examine the mediating role of fatigue and moderating role of rumination	Mobile Phone Addiction, Mind Wandering, Fatigue, Rumination	Mobile Phone Addiction Scale, Mind Wandering Scale (Chinese Version), Fatigue Assessment Scale (FAS), Ruminative Response Scale, Self‐Report Questionnaires, Confirmatory Factor Analysis, Data Collection via Convenient Sampling and Survey Posters, Ethical Approval, Informed Consent
Vogelgesang et al. 2022; Early shift of attention is not regulated by mind wandering in visual search	22 participants (mean age 26; 16 female) with normal vision and no neurological or psychiatric disorders	To investigate whether early attentional mechanisms are downregulated during mind wandering or if only motor responses are slowed	Reaction times, eye movements	Visual search task with target detection, recording eye movements and manual responses, unpredictable thought probes
Marchetti et al. 2015; Theory of mind and the whole brain functional connectivity: behavioral and neural evidences with the Amsterdam Resting State Questionnaire	670 Italian subjects for psychometric properties; 28 participants for neural correlates	To test psychometric properties of ARSQ and explore neural correlates related to Theory of Mind (ToM) factor	Functional connectivity (fMRI), reliability of ARSQ	Psychometric testing of ARSQ, functional MRI for neural correlates, analysis of within‐lobe FC and between‐group comparisons
Yeh et al. 2017; Switching to the rubber hand	36 adults (students and general public) with normal or corrected vision, recruited from National Taiwan University and surrounding neighborhoods	To investigate the relationship between the Rubber Hand Illusion (RHI) and higher cognitive functions	Switch cost, SART score, RHI onset time, attention control questionnaire	RHI induction with synchronous and asynchronous stroking, task switching, Sustained Attention to Response Task (SART), attentional control scale
El Haj and Nandrino; Intentional and unintentional mind‐wandering in Korsakoff syndrome	31 patients with KS (mean age 57.81) and 33 control participants (mean age 55.91)	To investigate intentional and unintentional mind wandering in patients with Korsakoff syndrome (KS)	Mind wandering questionnaire, cognitive assessment, depression scale	Questionnaire on mind wandering, cognitive assessment (MoCA, digit span, Stroop test), Hospital Anxiety and Depression Scale (HADS)
Maillet et al. 2018; Age‐related differences in mind‐wandering in daily life	31 young adults (mean age 21.53) and 20 older adults (mean age 70.70)	To assess age‐related differences in mind wandering frequency, relationship between affect and mind wandering, and content of mind wandering	Mind wandering frequency, thought content, affect	Experience sampling using MetricWire app, surveys on mind wandering, thought content, and affect, administered over 1 week
Jackson and Balota 2012; Mind‐wandering in younger and older adults: converging evidence from the sustained attention to response task and reading for comprehension	54 younger adults (29 female) and 62 older adults (40 female)	To investigate age‐related changes in mind wandering using the Sustained Attention to Response Task (SART) and reading for comprehension task	Reaction times, self‐reported mind wandering	SART with thought probes, reading for comprehension task, self‐reported mind wandering, cognitive and motivational measures
Bertossi and Ciaramelli 2016; Ventromedial prefrontal damage reduces mind‐wandering and biases its temporal focus	Seven patients with vmPFC lesions, 11 control patients with lesions not involving vmPFC, 20 healthy individuals	To investigate the role of the ventromedial prefrontal cortex (vmPFC) in mind wandering	Mind wandering rates, daydreaming frequency, thought content	Cognitive tasks (WM task, CRT task, Passive task), thought probes, self‐report daydreaming scale (Imaginal Processes Inventory)
Besten et al. 2023; The impact of mood‐induction on maladaptive thinking in the vulnerability for depression	82 participants (42 high in susceptibility to negative affect, 40 low in susceptibility to negative affect)	To investigate the adjustability of mind wandering content and characteristics in individuals with varying susceptibility to negative affect	SART performance, Positive and Negative Affect Scale (PANAS)	SART with positive fantasizing and stress induction sessions, PANAS, self‐related concerns incorporated into SART
Deng et al. 2022; The impacts of mind‐wandering on flow: examining the critical role of physical activity and mindfulness	429 Chinese college students (103 females; mean age 19.62)	To investigate the relationship between mind wandering and flow, and the potential mediation effects of physical activity and mindfulness	Mind wandering levels, physical activity, mindfulness, flow	Cross‐sectional study with questionnaires (MWQ, IPAQ, MAAS, S‐DFS), descriptive statistics, bivariate correlation, multiple mediation model
Belo et al. 2023; The effect of familiarity on neural tracking of music stimuli is modulated by mind wandering	41 participants (22 females; mean age 28.93)	To investigate the impact of familiarity and mind wandering on cortical tracking of continuous music stimuli	Neural activity, mind wandering levels, familiarity	Listening to music stimuli, EEG recording, stimulus reconstruction approach, linear mixed models, behavioral questions
Silva et al. 2018; Ruminative minds, wandering minds: effects of rumination and mind wandering on lexical associations, pitch imitation and eye behaviour	62 English‐speaking students (31 males, 31 females; mean age 23.82)	To investigate the effects of rumination and mind wandering on lexical associations, pitch imitation, and eye behavior	Rumination Inventory (RI), Dundee Stress Questionnaire (DSQ), word associations, pitch imitation, eye movements	Rumination induction/control, word association task with an Embodied Conversational Agent, questionnaires, eye tracking
Diaz et al. 2013; The Amsterdam Resting‐State Questionnaire reveals multiple phenotypes of resting‐state cognition	813 participants (online sample), 68 participants (fMRI sample), 89 participants (EEG sample)	To identify phenotypes of resting‐state cognition using the Amsterdam Resting‐State Questionnaire (ARSQ)	Resting‐state cognition dimensions, psychometric scales	Self‐report ARSQ, factor analysis, resting‐state fMRI, resting‐state EEG, correlation with psychometric scales
Koelsch et al. 2019; Heroic music stimulates empowering thoughts during mind‐wandering	62 participants (37 females; mean age 24.9)	To investigate the influence of heroic and sad music on the valence and nature of thoughts during mind wandering	Thought probes, heart rate (ECG	Listening to musical excerpts (heroic and sad), thought probes, questionnaires, electrocardiogram (ECG)
Wang et al. 2021; Mediating role of rumination and negative affect in the effect of mind‐wandering on symptoms in patients with obsessive‐compulsive disorder	100 patients with OCD (55 females, 45 males), 100 healthy controls (54 females, 46 males)	To explore the relationship between negative affect, mind wandering, rumination, and obsessive‐compulsive symptoms	Obsessive‐Compulsive Inventory, Beck Anxiety Inventory, Beck Depression Inventory, Mind Wandering Scale, Ruminative Response Scale	Questionnaires on obsessive‐compulsive symptoms, anxiety, depression, mind wandering, and rumination, statistical analysis
Gable et al. 2019; When the muses strike: creative ideas of physicists and writers routinely occur during mind wandering	Study 1: 45 physicists, 53 professional writers; Study 2: 27 physicists, 60 professional writers	To investigate how often creative ideas are generated during mind wandering and their characteristics compared to on‐task ideas	Diary reports, idea ratings, “aha” moments	Two‐week diary study, daily reporting of most creative idea, assessment of idea context, creativity, and importance, follow‐up survey
Zhao et al. 2023 The relationship between schizotypal personality features and mind wandering among college students during COVID‐19 pandemic: a moderator of depression	Study 1: 153 Chinese college students; Study 2: 557 Chinese college students	To explore the relationship between schizotypal personality features, mind wandering, and depression among college students during the COVID‐19 pandemic	Schizotypal Personality Questionnaire (SPQ), Mind‐Wandering Questionnaire (MWQ), Beck Depression Inventory (BDI)	Longitudinal study (Study 1), cross‐sectional study (Study 2), online self‐reported surveys using SPQ, MWQ, and BDI
Maillet et al. 2019; Aging and the wandering brain: age‐related differences in the neural correlates of stimulus‐independent thoughts	29 young adults (mean age 22), 22 older adults (mean age 70)	To investigate age‐related differences in the neural correlates of stimulus‐independent thoughts	fMRI data, thought probes	fMRI scans during rest with thought probes, assessment of thought content and focus, neural activation analysis
Coulborn et al. 2020; Effect of tDCS over the right inferior parietal lobule on mind‐wandering propensity	33 participants (six males, aged 18–23)	To investigate whether transcranial direct current stimulation (tDCS) targeting the right inferior parietal lobule (IPL) could modulate mind wandering propensity	SART performance, thought‐probes, tDCS perception questionnaire	tDCS over right IPL, SART with thought‐probes, within‐subjects double‐blind, counterbalanced design, electric field estimation using SimNIBS
Voss et al. 2018; A new approach to differentiate states of mind wandering: effects of working memory capacity	Experiment 1a: 20 undergraduate students; Experiment 1b: 34 undergraduate students	To investigate the process of mind wandering, combining self‐caught and probe‐caught methods, and examining the relationship to working memory capacity	OSPAN task performance, self‐caught mind wandering, probe‐caught mind wandering	OSPAN task, Mindfulness Breath Meditation Task, self‐caught method, probe‐caught method, E‐prime Psychology Software
Smallwood et al. 2014; Going AWOL in the brain: mind wandering reduces cortical analysis of external events	22 participants (six men, 16 women)	To investigate whether mind wandering is associated with reduced cortical analysis of external events	P300 event‐related potential, thought probes	Sustained attention to response task (SART), thought probes, ERP recording, analysis of P300 amplitude, regression analysis
Bruno et al. 2023; Distinct electrophysiological signatures of intentional and unintentional mind‐wandering revealed by low‐frequency EEG markers	26 participants (12 females, mean age 25 years, standard deviation 4.3 years)	To explore the electrophysiological differences between intentional and unintentional mind wandering	EEG markers, self‐reports of mind wandering	Sustained attention to response task (SART), EEG recordings, univariate and multivariate pattern analyses, thought probes
Mrazek et al. 2013a; Mindfulness training improves working memory capacity and GRE performance while reducing mind wandering	48 undergraduate students (14 male, 34 female; mean age 20.83)	To examine whether mindfulness training decreases mind wandering and improves cognitive performance	GRE scores, working memory capacity (OSPAN), mind wandering reports	2‐week mindfulness‐training course vs. nutrition class, GRE reading‐comprehension test, OSPAN task, thought sampling, self‐reports
Matiz et al. 2019; Spontaneous eye movements during focused‐attention mindfulness meditation	32 participants (19 female, 13 male; mean age 43.66, SD 12.16)	To explore ocular activity during mindfulness meditation and its potential as a marker for mind wandering episodes	Eye movement measurements (EEG), power spectral density	Mindfulness‐Oriented Meditation (MOM) training, focused‐attention mindfulness meditation (FAM), instructed mind wandering (IMW), EEG recordings, ICA algorithm
Zhang 2020; Mind‐wandering: what can we learn from eye movements?	42 undergraduates (mean age 18.76; 62% female), final sample size 33 participants	To explore the link between eye movements and mind wandering in different contexts involving complex cognitive processing	Visual search performance, eye‐tracking data, response time, recognition, recall	Visual search task, scene encoding task, reading comprehension task, eye‐tracking, thought probes, questionnaires
Floridou and Müllensiefen 2015; Environmental and mental conditions predicting the experience of involuntary musical imagery: an experience sampling method study	38 participants (24 females; age range 18–72)	To explore the environmental factors and psychological conditions related to involuntary musical imagery (INMI) in everyday life	INMI occurrence, contextual information, environmental conditions, mood state	Experience sampling method (ESM), Experience Sampling Booklet (ESB), Experience Sampling Forms (ESF), Bayesian networks, self‐reports, rating scales
Schaefer et al. 2014; Dynamic network participation of functional connectivity hubs assessed by resting‐state fMRI	108 participants (47 males, 61 females; mean age 37.71)	To investigate the dynamic participation of functional connectivity hubs in large‐scale brain networks and their relationship to self‐generated thoughts and age	Resting‐state fMRI data, connectivity clustering, self‐reports of thoughts	Resting‐state functional magnetic resonance imaging (rs‐fMRI), T1 anatomical scan, connectivity clustering, Bayesian networks, statistical analysis
Smallwood and O'Connor 2011; Imprisoned by the past: unhappy moods lead to a retrospective bias to mind wandering	Experiment 1: 59 participants; Experiment 2: 82 participants (54 females; mean age 23.6)	To assess whether unhappy moods lead to a greater frequency of past‐related mind wandering	Post‐task questionnaire (Experiment 1), experience sampling probes (Experiment 2), Beck Depression Inventory (BDI)	Mood induction via video (Experiment 1) and Velten mood‐induction procedure (Experiment 2), choice reaction time task, self‐reports of thoughts
Wing 2017; Mind‐wandering and mood repair: the role of off‐task thought in the sustainment of negative mood	79 participants (University of Kansas undergraduates)	To investigate the relationship between mind wandering and mood repair following the induction of a negative mood state	Beck Depression Inventory‐2nd Edition (BDI‐II), working memory task (dual N‐back), thought probes, mood check	Negative mood induction, choice reaction time (CRT) task, thought probes during CRT task, multilevel growth modeling analyses
Mildner and Tamir 2021; The people around you are inside your head: social context shapes spontaneous thought	| Study 1: 87 participants (age M = 19.9, SD = 1.9); Study 2: target sample size 80 participants	To assess the impact of social context on the content of spontaneous thought during mind wandering	Behavioral measures, neural activity, self‐reports of thoughts	Study 1: Solitude condition, control condition, mind wandering task, fMRI scanning; Study 2: Social presence condition, experience sampling, mind wandering task
Polychroni et al. 2021; Introspection confidence predicts EEG decoding of self‐generated thoughts and meta‐awareness	46 participants (28 females; age range 18–43; MAge = 25.9)	To investigate the relationship between introspection confidence, mind wandering, and meta‐awareness	EEG recordings, self‐reports of experiential state judgments, confidence ratings, audiobook listening assessment	Audiobook listening task, thought probes, multivariate pattern classification analysis, MATLAB, Psychophysics Toolbox
Diaz et al. 2013; The Amsterdam Resting‐State Questionnaire reveals multiple phenotypes of resting‐state cognition	813 participants (online sample), 68 participants (fMRI sample), 89 participants (EEG sample)	To identify phenotypes of resting‐state cognition using the Amsterdam Resting‐State Questionnaire (ARSQ	Resting‐state cognition dimensions, psychometric scales	Self‐report ARSQ, factor analysis, resting‐state fMRI, resting‐state EEG, correlation with psychometric scales
Mowlem et al. 2016; Validation of the mind excessively wandering scale and the relationship of mind wandering to impairment in adult ADHD	Study 1: 41 adults with ADHD, 47 controls; Study 2: 81 adults with ADHD, 30 controls	To investigate excessive mind wandering (MW) in adult ADHD using the Mind Excessively Wandering Scale (MEWS)	Mind Excessively Wandering Scale (MEWS), Barkley Adult ADHD Rating Scale (BRS), Conners’ Adult ADHD Rating Scales (CAARS), Affective Lability Scale–Short Form (ALS‐SF), Weiss Functional Impairment Rating Scale–Self‐Report (WFIRS‐S)	Psychometric assessment, principal components analysis (PCA), independent *t*‐tests, Mann–Whitney *U*‐tests, receiver operating characteristic (ROC) analysis, hierarchical multiple regression
Kase and Kawagoe 2021; Life skills link to mind wandering among university students: an exploratory study	53 participants (47 women, six men; mean age 19.2)	To investigate the relationship between life skills and two types of mind wandering (state MW and trait MW) among university students	Mind Wandering Questionnaire (MWQ), Life Skills Scale for Adolescents and Adults (LSSAA), self‐reports of MW occurrence during experimental task	Sustained attention response task (SART), multiple regression analysis, co‐occurrence network analysis, descriptive statistics, reliability coefficients
Filmer et al. 2019; For a minute there, I lost myself … dosage dependent increases in mind wandering via prefrontal tDCS	150 participants (96 females)	To investigate the effect of stimulation polarity and intensity on mind wandering while subjects undertook a repetitive cognitive task	Task‐unrelated thoughts (TUT) probes, SART performance	Sustained attention to response task (SART), transcranial direct current stimulation (tDCS) with varying dosages, Matlab, psychtoolbox, realistic volumetric approach (ROAST)
Borella et al. 2022; Cognitive and non‐cognitive variables influencing age‐related effect of mind wandering across the adult life span	210 participants (103 females, 107 males; age range 20–89)	To assess the effects of aging on mind wandering (MW) using a sustained attention to response task (SART)	Task‐unrelated thoughts (TUTs), stimulus‐independent thoughts (SITUTs), cognitive and noncognitive variables	Sustained attention to response task (SART), Short Portable Mental Status Questionnaire (SPMSQ), E‐Prime software, Likert scale, debriefing questionnaire
O'Callaghan et al. 2015; Shaped by our thoughts—a new task to assess spontaneous cognition and its associated neural correlates in the default network	31 healthy older participants (age range 53–79; average age 66.9; average education 14.9 years)	To develop and validate a novel task to assess the frequency and qualitative content of mind wandering in the context of low cognitive demands, and explore the associated neural correlates	Frequency and content of mind wandering, resting‐state functional connectivity, self‐reports of thoughts	Shape Expectations task, resting‐state functional magnetic resonance imaging (fMRI), functional connectivity analyses, scoring system for mind wandering
Varao‐Sousa and Kingstone 2018; Are mind wandering rates an artifact of the probe‐caught method? Using self‐caught mind wandering in the classroom to test, and reject, this possibility	259 students (86 participants included in analyses; 63 female; age range 17–28)	To investigate whether mind wandering (MW) rates obtained via the probe‐caught method are an artifact of the methodology by comparing self‐caught and probe‐caught MW in a classroom setting	Self‐caught MW reports, probe‐caught MW reports, memory test scores, interest and motivation ratings	Real‐world lecture setting, paper‐and‐pencil method, visual probes, multiple choice memory test, Likert scale ratings, experience reports
Jin et al. 2023; Decoding study‐independent mind‐wandering from EEG using convolutional neural networks	30 participants (13 females; ages 18–30; M = 23.33, SD = 2.81)	To train EEG classifiers using convolutional neural networks (CNNs) to track mind wandering across studies	EEG band‐frequency information (power), single‐trial ERP (stERP) patterns, connectivity matrices, mind wandering probes	Visual search task, sustained‐attention to respond task (SART), CNN models, leave‐N‐participant‐out cross‐validations, Biosemi ActiveTwo recording system
Hutt et al. 2023; Webcam‐based eye tracking to detect mind wandering and comprehension errors	Study 1: 105 University of New Hampshire students (ages 18–25); Study 2: 173 Prolific participants (ages 18–52)	To detect covert cognitive states during an online reading‐comprehension task related to task‐unrelated thought and comprehension using a webcam‐based eye‐tracker	Gaze measurements, task‐unrelated thought, reading comprehension	Webgazer (webcam‐based eye‐tracker), narrative anticipation task, online reading‐comprehension task, slicing analyses, calibration, real‐time gaze tracking
Chou et al. 2017; Maintenance and representation of mind wandering during resting‐state fMRI	72 right‐handed healthy adults (mean age 55 ± 18 years, 37 males)	To characterize the regulation and cerebral representation of mind wandering during resting‐state fMRI	Functional connectivity, resting‐state questionnaire (ReSQ), mind wandering domains	Resting‐state functional magnetic resonance imaging (fMRI), 3 Tesla GE scanner, Duke Brain Imaging and Analysis Center preprocessing pipelines, AAL template, Pearson correlation, Fisher's *z* transform
Brown and Forster 2022; Lapses in the person radar: ADHD symptoms predict difficulty in interpersonal distancing	Study 1a: 487 undergraduate participants (average age 20.71); Study 1b: 347 participants (average age 21.77)	To examine the frequency of unintentional lapses in interpersonal distancing and their relationship with childhood ADHD symptoms	Difficulties with Interpersonal Distancing and Awareness Scale (DIDAS), Childhood ADHD Symptoms Scale, Spontaneous and deliberate Mind wandering scales, Dispositional Hyperfocusing Scale	Online survey, 5‐point Likert scale, analysis of variance, mediation analysis, reliability analysis
Marcusson‐Lavertz et al. 2020; Sad mood and poor sleep are related to task‐unrelated thoughts and experience of diminished cognitive control	504 participants (241 men, 330 women, three reported “other”; mean age 25.87)	To investigate the association between sad mood, poor sleep, and mind wandering, and whether these associations reflect reduced effort in concentrating or diminished cognitive control	Task‐unrelated thoughts, unguided thoughts, sleep disturbances, mood induction, cognitive control	Internet‐based experiment, Patient Health Questionnaire‐2 (PHQ‐2), Control items, PROMIS sleep disturbance short form, Social Desirability Scale (SDS), Operation Span Task (OSPAN), Visuospatial 2‐Back with Thought‐Sampling Probes
Marcusson‐Clavertz et al. 2022; The contribution of latent factors of executive functioning to mind wandering: an experience sampling study	202 participants (18–42 years old, M = 24.95, SD = 5.11, 75 males)	To clarify the associations between executive functions and mind wandering using experience sampling methodology and latent scores for distinct executive functions	Task‐unrelated thoughts (TUTs), stimulus‐independent and task‐unrelated thoughts (SITUTs), experience sampling reports	Experience sampling methodology, digital wristband device (Pro diary), thought questionnaires, Short Imaginal Processes Inventory (SIPI), nine cognitive tasks, confirmatory factor analysis, latent scores
Kaur and Shwetha 2021; Entering the mental backstage: mindfulness and mind‐wandering among performing artists	66 performing artists (males = 24, females = 42; mean age = 20.95 years, SD = 1.589)	To explore the role of mindfulness and mind wandering among performing artists, specifically musicians, theatre artists, and dancers	Mindfulness, mind wandering (deliberate and spontaneous), creativity, socio‐demographics	Inventory of Creative Activities and Achievements (ICAA), Mindful Attention Awareness Scale (MAAS), Mind Wandering: Deliberate and Mind Wandering: Spontaneous Scales, one‐way ANOVA, self‐report indices
Unsworth and Robison 2017; The importance of arousal for variation in working memory capacity and attention control: a latent variable pupillometry study	175 participants (63.4% women, ages 18–35)	To explore the role of arousal in individual differences in working memory capacity (WMC) and attention control	Baseline pupil diameter, task‐evoked pupillary responses, working memory capacity (WMC), attention control, off‐task thinking	Multiple WMC and attention control tasks, thought probes, eye‐tracking (Tobii T120), structural equation models, latent variable analyses
Wiemers and Redick 2018b; The influence of thought probes on performance: does the mind wander more if you ask it?	149 students (final sample size used in analyses: 137)	To determine whether the presence of thought probes alters task performance and mind wandering frequency	SART performance, thought probes, working memory capacity (operation span, symmetry span)	Sustained attention to response task (SART), operation span, symmetry span, thought probes, demographics questionnaire, self‐report
Marcusson‐Clavertz et al. 2019; A daily diary study on maladaptive daydreaming, mind wandering, and sleep disturbances: examining within‐person and between‐persons relations	126 participants (mean age 30.04 years, SD = 10.65; 118 females, 30 males, four other)	To examine the within‐person and between‐person associations between mind wandering, maladaptive daydreaming, and sleep disturbances using a daily diary design	Mind wandering, maladaptive daydreaming, sleep disturbances, control items	Daily diary design, 16‐item Maladaptive Daydreaming Scale (MDS‐16), Mind Wandering Questionnaire (MWQ), PROMIS Sleep disturbance scale short form, Likert scale, attention checks
Stawarczyk et al. 2012; Using the daydreaming frequency scale to investigate the relationships between mind‐wandering, psychological well‐being, and present‐moment awareness	100 native French‐speaking individuals (42 men)	To investigate the relationships between mind wandering, psychological well‐being, and present‐moment awareness using the Daydreaming Frequency Scale	Mind wandering, psychological well‐being, present‐moment awareness, affect, future self‐thoughts	Daydreaming Frequency Scale (DDFS), Future Self Thoughts Questionnaire (FST), Positive and Negative Affect Schedule (PANAS), Center for Epidemiological Studies‐Depression Scale (CES‐D), Beck Anxiety Inventory (BAI), Sustained Attention to Response Task (SART) with thought‐probes, questionnaire
Axelrod et al. 2017; The default network and the combination of cognitive processes that mediate self‐generated thought	32 participants	To investigate the cognitive processes underlying self‐generated cognition using functional MRI and a wide range of cognitive tasks	Self‐generated cognition, self‐referential processing, scene construction, language‐related processing	Functional MRI, cognitive tasks (imagine past/future, episodic memory, empathizing, self‐referential judgments), GLM analysis, scene‐selective activity, language processing
Mowlem et al. 2019; Evaluating a scale of excessive mind wandering among males and females with and without attention‐deficit/hyperactivity disorder from a population sample	1484 participants (425 males, 1059 females; age range 16–83, M = 34.80, SD = 13.55)	To assess the factor‐structure, reliability, validity and measurement invariance of the Mind Excessively Wandering Scale (MEWS) and investigate sex differences in mind wandering, ADHD symptoms, impairment, and well‐being	Mind Excessively Wandering Scale (MEWS), Barkley Adult ADHD Rating Scale, Mind Wandering Spontaneous (MW‐S) scale, Mind Wandering Deliberate (MW‐D) scale, Affective Reactivity Index, Mental Health Continuum‐Short Form	Online survey, self‐report, factor analysis, validation measures, Cronbach's alpha, statistical tests
Tarailis et al. 2022; Data‐driven EEG theta and alpha components are associated with subjective experience during resting state	226 participants (*F* = 131, M = 95; age 23.41 ± 3.87)	To estimate associations between subjective experiences measured with the Amsterdam Resting‐State Questionnaire and data‐driven components of an electroencephalogram extracted by frequency principal component analysis	EEG components, subjective experience, resting state, mind wandering	Resting‐state EEG, Amsterdam Resting‐State Questionnaire (ARSQ), EEG data processing (MATLAB, EEGLAB), frequency principal component analysis (f‐PCA), source localization (sLORETA), Bayesian Pearson's correlation
Robison et al. 2019; Examining the effects of probe frequency, response options, and framing within the thought‐probe method	73 participants from the human subject pool at the University of Oregon (Experiment 1); 95 participants from the same pool (Experiment 2)	To investigate how manipulating probe frequency, response options, and framing within the thought‐probe method affects behavioral performance and responses to thought probes	Probe frequency, response options, framing, thought probes, SART performance	Sustained attention to response task (SART), thought probes, semantic SART, response times, errors, different probe conditions, random assignment
Brandmeyer and Delorme 2016; Reduced mind wandering in experienced meditators and associated EEG correlates	24 participants (three expert meditators, 10 nonexpert practitioners)	To assess the relation between mind wandering and meditation by testing groups of meditators with different experience levels and examining associated EEG correlates	Mind wandering, meditation experience, EEG activity	Seated concentration meditation, experience‐sampling probes, self‐reports, EEG, MATLAB psychophysics toolbox, customized USB keypad, randomized intervals
van Son et al. 2019a; Frontal EEG theta/beta ratio during mind wandering episodes	26 participants (healthy females between 18 and 30 years old, recruited at Leiden University)	To replicate and extend previous findings on the theta and beta effects for frontal TBR recordings and test if MW‐related changes in frontal TBR are related to attentional control	Frontal theta/beta ratio (TBR), mind wandering (MW), attentional control	Breath‐counting task, EEG recording, State‐Trait Anxiety Inventory (STAI‐t), Attentional Control Scale (ACS), button presses, EEG (ActiveTwo BioSemi system), exclusion criteria
Knyazev et al. 2012; EEG correlates of spontaneous self‐referential thoughts: a cross‐cultural study	Sample 1: 60 participants (32 men, 28 women; mean age 20.4, SD = 2.5); Sample 2: 58 participants (37 men, 21 women; mean age 41.2, SD = 20.8); Sample 3: 42 participants (26 men, 16 women; mean age 27.1, SD = 3.3)	To investigate the EEG correlates of spontaneous self‐referential thoughts across different cultures	Self‐referential thoughts, EEG activity, DMN, alpha activity	Resting‐state EEG, spontaneous thoughts questionnaire (STQ), Likert scale, principal components factor analysis, sLORETA, ICA, EEG recording (Neuroscan amplifiers, Quik‐Cap128 NSL), cephalic index
Krukow and Jonak 2022; Relationships between resting‑state EEG functional networks organization and individual differences in mind wandering	100 students (21–24 years old, males and females from different universities)	To investigate the relationships between resting‐state EEG functional networks organization and individual differences in mind wandering	Mind wandering (MW), resting‐state EEG, cognitive individual differences	Mind Wandering Questionnaire (MWQ), Raven's Standard Progressive Matrices (SPM) test, 64‐channel HydroCel Geodesic Sensor Net, resting‐state EEG, multichannel signal source, functional networks reconstruction, cognitive processing, screening criteria
Gil‐Jardiné et al. 2017; The distracted mind on the wheel: overall propensity to mind wandering is associated with road crash responsibility	954 drivers injured in a road crash (2013–2015)	To assess the impact of mind wandering trait and mind wandering state on road crash risk using a comparison between responsible and nonresponsible drivers	Mind wandering trait, mind wandering state, road crash responsibility, external distraction, alcohol use, psychotropic drug use, sleep deprivation	Responsibility case‐control study, adult emergency department of Bordeaux university hospital, interviews, standardized responsibility tool, self‐reports, mitigating factors, scoring, analysis
Burdett et al. 2016; Not all minds wander equally: the influence of traits, states and road environment factors on self‐reported mind wandering during everyday driving	502 participants (113 males, average age 44.4 years, SD = 14.0)	To explore differences in self‐reported mind wandering during everyday driving based on driver demographics, cognitive traits, personal states, and road environment factors	Mind wandering (MW), cognitive traits, personal states, road environment factors	Mindful Attention and Awareness Scale (MAAS), Cognitive Failures Questionnaire (CFQ), Driver Behavior Questionnaire (DBQ), self‐reported mind wandering, demographic questions, driving situations questions, Likert scale
Martínez‐Pérez et al. 2021; Propensity to intentional and unintentional mind‐wandering differs in arousal and executive vigilance tasks	30 young adults (25 females, aged 18–26 years, M age = 20.77, SD = 1.8)	To examine participants' mind wandering (intentional and unintentional) while performing vigilance tasks that tap different components of vigilance	Mind wandering (intentional and unintentional), vigilance tasks (PVT, SART)	Psychomotor Vigilance Task (PVT), Sustained Attention to Response task (SART), thought probes, response options, within‐participants design, E‐Prime‐3, 5‐button Chronos device, verbal and written instructions
Seli et al. 2018; The awakening of the attention: evidence for a link between the monitoring of mind wandering and prospective goals	Two independent samples, each with 105 participants (Sample 1: 55 females, mean age 23.40; Sample 2: 61 females, mean age 21.79)	To examine the relation between individual differences in rates of self‐caught mind wandering and temporal monitoring of an unrelated response goal	Self‐caught mind wandering, temporal goal monitoring, prospective goals, sustained‐attention task, motivation	Time‐Based Prospective Memory Task, Sustained Attention to Response Task (SART), self‐caught mind wandering, temporal monitoring, intermittent thought probes, motivation assessment, response tasks, random assignment
Koelsch et al. 2021; Tormenting thoughts: the posterior cingulate sulcus of the default mode network regulates valence of thoughts and activity in the brain's pain network during music listening	33 participants (18 women, age‐range 20–31 years, mean = 23.1 years)	To investigate the neural correlates of positive and negative thoughts during mind wandering evoked by music	Mind wandering, valence of thoughts, default mode network (DMN), posterior cingulate sulcus, medial orbitofrontal cortex (mOFC)	Resting‐state fMRI, music stimuli (positive and negative valence), thought probes, data‐driven analysis, functional connectivity, pain network, participant ratings, MRI acquisition, pilot‐data, preparatory internet experiment
Kanske et al. 2016; Where the narcissistic mind wanders: increased self‐related thoughts are more positive and future oriented	135 participants (89 female, mean age 30.3 years, SD = 10.7 years)	To explore where the narcissistic mind wanders using an experience‐sampling approach and multilevel modeling	Mind wandering, narcissism, self‐related thoughts, thought content, valence, future orientation	Pathological Narcissism Inventory (PNI), Choice Reaction Time Task (CRT), Working Memory Task (WM), thought probes, Likert scales, multilevel modeling, participant recruitment, Ethics Commission approval
Finnigan et al. 2007; Alcohol and the wandering mind: a new direction in the study of alcohol on attentional lapses	24 participants (17 males, seven females)	To examine the influence of acute alcohol on attentional lapses while performing a sustained attention task (SART)	Mind wandering, acute alcohol ingestion, attentional lapses	Alcohol administration (vodka), Sustained Attention to Response Task (SART), Thinking Content component of the Dundee Stress State Questionnaire (DSSQ), health‐check questionnaire, Short Michigan Alcoholism Screening Questionnaire (SMAST), between‐group design, random allocation, control group, standard SART, demographic details, inclusion/exclusion criteria
Hawley et al. 2020; Technology supported mindfulness for obsessive compulsive disorder: self‐reported mindfulness and EEG correlates of mind wandering	71 treatment‐seeking individuals with a primary diagnosis of OCD	To examine the potential benefits of using a consumer grade EEG‐based biofeedback device (“Muse”) for mindfulness meditation practices in managing OCD symptoms	Mindfulness, OCD symptoms, EEG correlates of mind wandering	Randomized controlled study, Muse device, mindfulness meditation program, waitlist control, EEG recording, Five Factor Mindfulness Questionnaire (FFMQ), Yale‐Brown Obsessive Compulsive Scale (YBOCS), Latent Difference Score (LDS) models, recruitment, SCID‐5, exclusion criteria, informed consent
Kam et al. 2013; I do not feel your pain (as much): the desensitizing effect of mind wandering on the perception of others’ discomfort	Experiment 1: 19 individuals (12 females, seven males; mean age = 22.63 years, SD = 4.89); Experiment 2: 37 participants (25 females, 12 males; mean age = 22.3 years, SD = 3.31)	To examine whether mind wandering attenuates sensitivity to observing mild pain in others using ERPs and behavioral measures	Mind wandering, sensitivity to pain, ERP, behavioral measures, affective salience	Experiment 1: Event‐related potentials (ERPs), images of hands in painful/neutral situations, experience sampling, forced choice decisions, EEG recording, artifact rejection; Experiment 2: Pain ratings, photographs of hands, Likert scale, attention state reports, random intervals, control analyses
Miller 2021; Exploring the effects of interleaving on mind‐wandering	161 undergraduate students (final sample: 65 in blocked group, 60 in interleaved group)	To explore whether interleaving reduces mind wandering using a between‐subjects design	Interleaving, mind wandering, learning and memory, task performance	Bird images from 8 families (Orioles, Warblers, Finches, Swallows, Vireos, Grosbeaks, Thrushes, Chickadees), mind wandering probe, postassessment questionnaire, final classification test, random assignment, remote participation via Qualtrics, self‐report questionnaire, power analysis, exclusion criteria
Jubera‐Garcia et al. 2021; Local use‐dependent activity triggers mind wandering: resource depletion or executive dysfunction?	20 participants (Mage: 23.25 years, SDage: 2.40; 16 females)	To test the hypothesis that mind wandering could be related to the depletion of resources in primary task‐related networks, not necessarily changes in the executive system	Mind wandering (MW), resource depletion, executive functions, task‐related networks	Texture Discrimination Task (TDT), subjective (thought probes) and objective (phasic pupillary response) MW recording, four TDT sessions, stimulus presentation, darkened room, Mac OS X, Samsung SMB 1940 screen, EyeLink 1000 system, Matlab, Psychtoolbox, thought probes, pseudorandom points, informed consent, Ethics Committee approval
Cárdenas‐Egúsquiza and Berntsen 2022; Sleeping poorly is robustly associated with a tendency to engage in spontaneous waking thought	236 participants (121 men, 113 women, two reported “transgender”; mean age 41.32, SD = 11.73)	To examine the relationship between self‐reported sleep and spontaneous thought measures, independently of negative affect, age, and gender	Sleep quality, daytime sleepiness, insomnia symptoms, spontaneous waking thoughts	Comprehensive survey, self‐reported measures, Pittsburgh Sleep Quality Index (PSQI), Epworth Sleepiness Scale (ESS), Insomnia Severity Index (ISI), Morningness–Eveningness questionnaire (MEQ), Daydreaming Frequency Scale (DDFS),
				Spontaneous and Deliberate Mind Wandering Scales (MW‐S, MW‐D), Involuntary Autobiographical Memory Inventory (IAMI), Short Imaginal Process Inventory (SIPI), Positive and Negative Affect Schedule (PANAS), CloudResearch Prime Panels, pilot study, attention checks, preregistration, Ethics Committee approval
Arnicane et al. 2021; Validity of attention self‐reports in younger and older adults	40 younger adults (20–33 years, MAge = 24.6, SD = 3.6, 33 women) and 44 older adults (62–79 years, MAge = 69.8, SD = 3.9, 22 women)	To assess the correlation between self‐reported on‐task focus and task performance in younger and older adults	Mind wandering, attention self‐reports, task performance, current concerns, aging	Visual working memory task, reading task, Sustained Attention to Response Task (SART), thought probes, Concerns Questionnaire, self‐reports, attention ratings, task difficulty and interest ratings, MATLAB, Psychophysics Toolbox, Mini‐Mental State Examination (MMSE), informed consent, ethical guidelines
Cnudde et al. 2023; EEG complexity during mind wandering: a multiscale entropy investigation	24 individuals (17 females, seven males; M = 19.5 years, SD = 1.5 years, range = 18–22 years; final sample: 20 participants)	To investigate whether mind wandering impacts EEG signal complexity and explore if such effects differ across timescales and change in a context‐dependent manner	Mind wandering, EEG complexity, multiscale entropy, Navon's global and local processing task	EEG recording, multiscale entropy, experience sampling, thought probes, global and local processing task, behavioral performance, Navon's task, pseudorandom intervals, participant reports, 2 (mind wandering vs. on‐task) x 2 (global processing vs. local processing) repeated measures design, informed consent, Ethics Board approval
Godwin et al. 2023; Beyond mind wandering: performance variability and neural activity during off‐task thought and other attention lapses	31 participants (17 female, 17 male, one no response; age range 18–23, M = 20, SD = 1.6)	To study the characteristics of attention lapses using a metronome response task and experience sampling while recording fMRI data	Attention lapses, off‐task thought, mind wandering, task‐related interference, inattention	Metronome response task (MRT), thought probes, fMRI activation, default mode network (DMN), anterior cingulate cortex, inferior frontal gyrus, left hippocampus, functional connectivity, Siemens 3T Trio MRI scanner, T1‐weighted MPRAGE anatomical scan, functional T2*‐weighted echo‐planar scans, Georgia Institute of Technology, behavioral results, performance variability
Weber et al. 2024; Effects of task type on spontaneous alternations of attentional states	Experiment 1: 33 participants (18–22 years, University of Illinois); Experiment 2: 30 participants; Experiment 3: 29 participants; Experiment 4: 29 participants	To examine the effects of task type on spontaneous alternations between task focus and mind wandering, and to overcome prior methodological challenges	Spontaneous alternations, attentional states, task focus, mind wandering, task type	Mindfulness breath meditation task, self‐caught and probe‐caught experience sampling, PsychoPy, Pavlovia.org, Likert‐style rating scale; Experiment 2: Likert‐style rating scale for attentional focus; Experiment 3: Scene‐categorization continuous performance task (CPT), self‐caught and probe‐caught experience sampling; Experiment 4: Backward (metacontrast‐) masked visual detection task, externally oriented attention, self‐caught and probe‐caught experience sampling, Institutional Review Board approval
Dias da Silva et al. 2022; Revisiting consciousness: distinguishing between states of conscious focused attention and mind wandering with EEG	36 participants (23 female; mean age = 22.06, SD = 3.12)	To distinguish between states of conscious focused attention and mind wandering using EEG	Mind wandering, conscious focused attention, EEG, neural signatures	Visuomotor tracking task, event‐related potentials (ERP), spectral power, Mind Wandering Inventory, E‐prime 3.0, thought probes, EEG recording (g.Nautilus Research 32‐channel system), EEGLAB toolbox, Matlab, informed
				consent, Institutional Review Board approval
He et al. 2019; Thought control ability moderates the effect of mind wandering on positive affect via the frontoparietal control network	368 participants (103 men, mean age = 19.38 ± 1.35)	To explore the relationships among mind wandering, emotions, and thought control ability, and how thought control ability moderates the effect of mind wandering on positive affect via the frontoparietal control network	Mind wandering, thought control ability (TCA), positive affect (PA), negative emotion (NE), frontoparietal control network (FPCN)	Resting‐state functional magnetic resonance imaging (rsfMRI), Daydreaming Frequency Scale (DDFS), Mind Wandering Frequency Scale (MWFS), Positive Affect and Negative Affect Scale (PANAS), Beck Depression Inventory (BDI), Beck Anxiety Inventory (BAI), Urban Happiness Index Scale (UHIS), Oxford Happiness Inventory (OHI), Center for Epidemiological Studies‐Depression Scale (CES‐D), Thought Control Ability Questionnaire (TCAQ), Siemens 3T Trio scanner, 12‐channel head coil, Institutional Review Board approval, informed consent
Baird et al. 2011; Back to the future: autobiographical planning and the functionality of mind‐wandering	47 participants (age range 17–32 years)	To explore the hypothesis that one potential function of spontaneous thought is to plan and anticipate personally relevant future goals	Mind wandering, autobiographical planning, future‐focused thought, working memory capacity	Choice Reaction Time Task, Operation Span (OSPAN) task, thought probes, on‐task and off‐task mental states, temporal focus (past, present, future), cognitive dimension (self‐related, goal‐directed), experience sampling, automated OSPAN, serial letter recall, alternating processing task, math equations, response deadline, accuracy criterion, inter‐rater reliability
Song and Wang 2012; Mind wandering in Chinese daily lives—an experience sampling study	165 undergraduates (115 females, aged 18–29 years)	To examine mind wandering in a non‐Western population and its relationship to personal life and contextual factors	Mind wandering, daily life, experience sampling, prospective thinking	Experience sampling, mind wandering questionnaire, episodic and semantic components, internal and external cues, personal life relevance, current tasks, attention orientation, arousal states, mood, psychoactive substance use, meta‐awareness, informed consent, ethical principles, institute review board approval
Franklin et al. 2014; Tracking distraction: the relationship between mind‐wandering, meta‐awareness, and ADHD symptomatology	105 participants (71 females, M age = 23.1, SD = 7.4)	To comprehensively assess the relationship between mind wandering and ADHD symptomatology in an adult community sample using laboratory measures and experience sampling during daily life	Mind wandering, ADHD symptomatology, meta‐awareness, detrimental mind wandering, useful mind wandering	Sustained Attention to Response Task (SART), Reading and Mind‐Wandering Task, thought probes, Involuntary Personal Imagination Inventory (IPI), Attention‐Related Cognitive Errors Scale (ARCES), Mindful Attention Awareness Scale (MAAS‐LO), Memory Failures Scale (MFS), Self‐Consciousness Scale, Conners Adult ADHD Rating Scale (CAARS‐S:SV), ADHD Self‐Report Scale (ASRS‐V1.1), Reading Span Task (RSPAN), Stop‐Signal Task, executive functioning, creativity, University of British Columbia, recruitment flyers, informed consent, experience sampling
Liu et al. 2023; Examining the effects of a modified SART when measuring mind‐wandering	179 participants (M = 20.281 years, SD = 0.547; 123 females, 62 males)	To modify the sustained attention to response task (SART) to better capture the dynamics of mind wandering over time and quantify the degree of mind wandering	Mind wandering (MW), modified SART (mSART), error rate, Mean RTs, RT CV, d’	Modified SART (mSART) paradigm
Hawkins et al. 2022; Modeling distracted performance	Experiment 1: 20 undergraduate psychology students (eight female, 12 male, age range 20–32, M = 23.85, SD = 2.9); Experiment 2: 25 undergraduate psychology students (19 females, six males, M = 23.1, SD = 4.0)	To develop and experimentally test the first integrated cognitive process model that quantitatively explains all stationary features of behavioral performance in the SART	Mind wandering, SART, cognitive process model, rhythmic response process, self‐reported distraction	Experiment 1: SART, custom program (Psychopy), MacBook Pro, thought probes, 4‐point Likert scale, pseudo‐random distribution; Experiment 2: SART, fixed ISI condition, random ISI condition, separate blocks, order counterbalanced, preliminary analysis, strong order effects, fixed and random ISI conditions, self‐reported mind wandering, G*Power, manipulation checks, invalid data criteria
Tomescu et al. 2022; Spontaneous thought and microstate activity modulation by social imitation	43 participants (24 men, 19 women, mean age 25.7, age range 20–42, SD = 5.1)	To investigate the modulation of resting‐state spontaneous thoughts and microstate activity by social imitation, and how it affects behavioral states and personality traits	Mind wandering, social imitation, EEG microstate analysis, behavioral states, personality traits	Social imitation (SI) task, control (CTRL) task, imitation of arm movements, resting‐state EEG, oxytocin (OXT), EEG microstate analysis, Global Field Power (GFP), k‐means cluster analysis, Neo Personality Inventory–Revised (NEO PI‐R), Amsterdam Resting‐State Questionnaire 2.0 (ARSQ), Visual Analog Scales (VAS), Inclusion of Others in the Self (IOS), two‐tailed Wilcoxon sign‐ranked paired tests, false discovery rate (FDR) correction, Gaussian distribution testing, Shapiro–Wilk normality test, Cartool software, Ethics Committee approval, informed consent
Zedelius et al. 2020; Lay theories of the wandering mind: control‐related beliefs predict mind wandering rates in‐ and outside the lab	Study 1: 505 adults (MTurk), Study 2: 394 American adults (CriticalMix), Study 3: 58 psychology students, Study 4: 100 psychology students, Study 5: 196 students, Study 6: 316 university studentsTo examine how lay theories (beliefs about the controllability of mind wandering) affect thought control strategies and mind wandering ratesMind wandering, control‐related beliefs, thought control strategies, intrusive thoughts	Developed and administered TOMW scale, experimental manipulation of lay theories, reading tasks, multiple‐choice comprehension questions, and various psychological scales across studies
Michael et al. 2017; Thoughts and sensations, twin galaxies of the inner space: the propensity to mind‐wander relates to spontaneous sensations arising on the hands	29 participants (22 female, mean age 21.8 ± 2.6, age range: 20–33)	To investigate whether spontaneous sensations (SPS) vary as a function of the individual propensity to generate spontaneous thoughts (mind wandering)	Mind wandering, spontaneous sensations (SPS), self‐awareness, interception	Mind Wandering Questionnaire (MWQ), frequency and propensity to mind‐wander, SPS task, sociodemographic and health characteristics questionnaire, informed written consent, University of Franche‐Comté, power calculation, quiet and normally lit room, ambient temperature 20–23°C, Helsinki Declaration
Luelsberg et al. 2022; Neuropsychological features of mind wandering in left‐, right‐ and extra temporal lobe epilepsy	29 right‐handed patients with right‐, left‐, or extra‐temporal lobe epilepsies	To evaluate whether parameters of mind wandering are related to material specific memory in patients with a left‐, right‐, or extra‐temporal lobe epilepsy	Mind wandering, material specific memory, temporal lobe epilepsy, executive functions	Sustained Attention to Response Task (SART), experience sampling probes, meta‐awareness, temporal orientation, Verbal List Learning and Memory Test (VLMT), Design List Learning (DCS‐R), EpiTrack test
				battery, verbal memory, visual/figural memory, executive functions,
Seli et al. 2016; Assessing the associations among trait and state levels of deliberate and spontaneous mind wandering	102 undergraduate psychology students (mean age 19.61; 71 females)	To evaluate whether trait‐level deliberate and spontaneous mind wandering map onto state levels of these subtypes of mind wandering	Mind wandering, deliberate mind wandering, spontaneous mind wandering, trait‐level, state‐level	Metronome Response Task (MRT), sustained‐attention task, key‐press response, constant sequence of tones, University of Waterloo, partial course credit
Robison et al. 2017; The neurotic wandering mind: an individual differences investigation of neuroticism, mind‐wandering, and executive control	213 undergraduate students (128 females, average age 19.40 years, SD = 2.32)	To investigate the relationship between neuroticism, mind wandering, and executive control	Mind wandering, neuroticism, executive control, working memory capacity, attention control	Operation span, Symmetry span, Reading span, Psychomotor vigilance task (PVT), Stroop, Antisaccade, thought probes, Big Five Inventory questionnaire, University of Oregon, informed consent, course credit, confirmatory factor analysis, structural equation modeling, attention control tasks, personality questionnaire, reaction time, psychomotor vigilance, sustained attention
Kam et al. 2021; Distinct electrophysiological signatures of task‐unrelated and dynamic thoughts	39 participants (13 females, 32 males; M = 20.1 years, SD = 1.83)	To determine the electrophysiological signatures of task‐unrelated and dynamic thoughts	Mind wandering, task‐unrelated thoughts, dynamic thoughts, electrophysiological signatures, alpha power, alpha‐power variability, P3 event‐related potentials (ERPs)	Simple attention task, thought probes, 7‐point Likert scale, definitions and example scenarios, EEG recording (BioSemi ActiveTwo System), bandpass‐filtering, independent component analysis, fastICA toolbox, EEGLAB, spherical spline interpolation, artifact detection, common average reference, segmented epochs, Matlab, University of California, Berkeley, informed consent, Institutional Review Board approval
Albert et al. 2018; Linking mind wandering tendency to risky driving in young male drivers	30 young male drivers (aged 18–21)	To test whether mind wandering tendency predicts risky driving behavior in young male drivers and whether this relationship is mediated by driver vigilance and moderated by executive control capacity	Mind wandering tendency, risky driving, driver vigilance, executive control capacity	Sustained Attention to Response Task (SART), Daydreaming Frequency Scale (DDFS), driving simulator, mean speed, horizontal eye movements, eye‐tracking (FaceLab 5), Color‐Word Interference test (CWIT), selective attention, goal maintenance, working memory capacity, recruited from greater Montreal area, previous studies on alcohol and driving performance, exclusion criteria, $75.00 compensation, informed consent
Staub et al. 2014; Investigating sustained attention ability in the elderly by using two different approaches: inhibiting ongoing behavior vs. responding on rare occasions	30 younger adults (21 females, mean age 24.8 years, range 18–32) and 30 older adults (16 females, mean age 65.2 years, range 60–74)	To evaluate sustained attention performance in younger and older individuals using two versions of the sustained attention to response task (SART) with different response modes	Sustained attention, elderly, younger adults, Go/No‐Go task, traditionally formatted task (TFT), cognitive control mechanisms	Sustained Attention to Response Task (SART), Go/No‐Go task, response inhibition, digits 1–9, randomized order, varying interstimulus interval (ISI), five allocated digit sizes, motivation component of Dundee Stress State Questionnaire (DSSQ), thinking content component of DSSQ, task unrelated thoughts (TUT), NASA‐Task Load Index (NASA‐TLX), written informed consent, local Ethics Committee approval
Giannandrea et al. 2018; Effects of the mindfulness‐based stress reduction program on mind wandering and dispositional mindfulness facets	60 participants interested in MBSR training (mean age = 35.7, range 21–60, SD = 12.1)	To investigate the effects of mindfulness‐based stress reduction (MBSR) training on mind wandering and dispositional mindfulness facets	Mind wandering, dispositional mindfulness facets, mindfulness‐based stress reduction (MBSR), sustained attention, attentional lapses	Sustained Attention to Response Task (SART), Five Facets Mindfulness Questionnaire (FFMQ), SART thought probes, stratified random assignment based on meditation experience, mixed‐design repeated measure ANOVA, correlation analyses, regression analysis, random sequence, Go/No‐Go task, instructions for participants, short training session, CONSORT flowchart, written informed consent, G‐Power 3 software
Chen et al. 2019; Mind wandering in schizophrenia: a thought‐sampling study	58 chronic schizophrenia patients and 56 matched healthy controls	To examine mind wandering in schizophrenia patients with a thought‐sampling experiment embedded in a rapid go/no‐go task and its relationship to psychotic symptoms	Mind wandering, schizophrenia, thought‐sampling, psychotic symptoms, rapid go/no‐go task	Sustained Attention to Response Task (SART), thought probes, randomized order, virtual blocks, presentation of digits (1–9), nontargets (1–2, 4–9), target (3), 900‐ms mask, 250‐ms stimulus presentation, off‐task thoughts, task interest, working memory, Intrinsic Motivation Inventory for Schizophrenia Research (IMI‐SR), Chinese version of Letter–Number Span test, Chinese version of Wechsler Adult Intelligence Scale‐Revised (WAIS‐R), Annett Handedness Scale, written informed consent, Ethics Committee approval
Leszczynski et al. 2017; Mind wandering simultaneously prolongs reactions and promotes creative incubation	82 adults (50 female, mean age 23 years, SD = 3.59; *N* = 28, *N* = 26, *N* = 28 for experiments 1–3, respectively)	To show that mind wandering relates simultaneously to both behavioral costs (prolonged reaction times) and benefits (improved creative problem solving and daily routine planning)	Mind wandering, prolonged reaction times (RT), creative incubation, sustained attention, creative problem solving, daily routine planning	Sustained Attention to Response Task (SART), compound remote associates test (CRA), daily planning task (DPT), behavioral costs, creative insight index, stream of stimuli, target and nontarget items, experience sampling procedure, random intervals, questions on mind wandering/task‐unrelated thoughts (TUTs), Ethics Committee of the University of Bonn Medical Center, written informed consent, Kendall's tau, nonparametric rank‐correlation test
Vinski and Watter 2013; Being a grump only makes things worse: a transactional account of acute stress on mind wandering	124 undergraduate students (51 in experimental group, 26 females; 73 in control group, 43 females)	To investigate the influence of acute stress on mind wandering	Acute stress, mind wandering, negative mood, stress induction, cognitive resources	Positive and Negative Affect Schedule (PANAS‐X), Trier Social Stress Test (TSST), Sustained Attention to Response Task (SART), variable response time, errors, stressor‐related thoughts, pupil diameter, baseline state mood, high‐stress and low‐stress conditions, complex verbal arithmetic, impromptu speech, verbal arithmetic without panel, informal talk, informed consent, University's online experiment scheduling system, partial course credit
Albert et al. 2023; A randomized controlled pilot trial of brief online mindfulness training in young drivers	26 drivers, aged 21–25	To test whether brief online mindfulness training (MT) reduces unsafe driving by enhancing recognition (meta‐awareness) of mind wandering (MW) and reducing its occurrence	Mind wandering, mindfulness training, driver distraction, meta‐awareness, driving behavior	Pre–post (T1, T2) design, randomized, active placebo‐controlled, double‐blinded, brief online mindfulness training (MT), progressive muscle relaxation (PMR), custom website, driving simulator, thought probes,
				adherence, acceptability, exclusion criteria, screening, State Mindfulness Scale (SMS), postsession questionnaire, compensation, Ethics Board approval, Montreal, Canada, Douglas Hospital Research Centre, McGill University
Kane et al. 2021; Testing the construct validity of competing measurement approaches to probed mind‐wandering reports	1108 undergraduate students from two US institutions (UNCG and WCU)	To explore the construct validity of probed mind wandering reports with a combined experimental and individual‐differences approach	Mind wandering, task‐unrelated thought (TUT), probed reports, construct validity, consciousness‐related constructs	Four different thought‐probe types, two cognitive tasks, Antisaccade letters (ANTI‐LET), Semantic Sustained Attention to Response Task (SART), Dundee Stress State Questionnaire (DSSQ1 and DSSQ2), AD/HD Rating Scale IV–Self‐Report Version, Cognitive Failures Questionnaire–Memory and Attention Lapses (CFQ–MAL), Creative Achievement Scale (CAS), Imaginal Process Inventory (IPI), Schizotypy–Magical Ideation scale, Metacognitive Prospective Memory Battery, Mind Wandering–Deliberate scale, Mind Wandering–Spontaneous scale, Spontaneous Activity Questionnaire scale (SAQ), White Bear Suppression Inventory, Attentive Responding Scale (ARS), UNCG, WCU, E‐Prime software, forced‐choice response, content probes, intentionality probes, depth probes, demographics, data analysis, correlation effect‐size estimates, written informed consent
Zhou et al. 2024; Childhood adversity and mind wandering: the mediating role of cognitive flexibility and habitual tendencies	601 Chinese subjects (378 females, Mage = 19.37)	To assess the associations between childhood adversity, and mind wandering, and to evaluate the mediating roles of cognitive flexibility, and habit tendencies	Childhood adversity, mind wandering, cognitive flexibility, habitual tendencies, deliberate mind wandering, spontaneous mind wandering	Hierarchical regression analyses, serial mediation analyses, Childhood Trauma Questionnaire (CTQ), Cognitive Flexibility Inventory (CFI), Creature of Habit Scale (COHS), Mind Wandering: Deliberate (MW‐D) and Mind Wandering: Spontaneous (MW‐S) scales, online platform (Chinese online survey platform), recruitment through campus life and study related mailing lists and online discussion groups, sample size determination, Structural Equation Modeling (SEM), rule of thumb, pragmatic approach
Sheffield 2023; Investigating meditation's potential to enhance working and episodic memory performance using EEG	60 participants (aged 18–45)	To investigate the effects of three types of meditation on working memory (WM) and episodic memory (EM) using both behavioral and EEG measures	Meditation, working memory (WM), episodic memory (EM), EEG, attention, cognitive functions	Focused attention meditation (FAM), open monitoring meditation (OM), loving kindness meditation (LKM), control sham meditation (SM), baseline demographic questionnaires, self‐report measures, anxiety, positive and negative affect, state mindfulness, depth of meditation, resting state EEG, Episodic Memory Task, N‐back Task, mixed measures ANOVA, time‐frequency analysis, theta, beta, gamma, alpha, delta power, comparison analysis
Lu et al. 2024; Scale for time and space experience in anxiety (STEA): phenomenology and its clinical relevance	19 subjects for STEA validation (13 females, six males) and 48 subjects for cSTEA validation (29 females, 19 males)	To introduce and validate the Scale for Time and Space Experience of Anxiety (STEA) and its shorter clinical version (cSTEA)	Anxiety, time and space experience, STEA, cSTEA, phenomenology, clinical relevance	STEA, cSTEA, Beck Anxiety Inventory (BAI), Beck Depression Inventory (BDI), spontaneous mind wandering (MWS), deliberate mind wandering (MWD), breathing therapy, hierarchical regression analyses, statistical feature selection, pre‐ and post‐therapeutic analysis, visual analog scale format, random manner, expert opinion, exploratory investigation, ongoing study, pharmacological therapy, serotoninergic drugs, cognitive behavioral therapy, healthy control group, Student's *t*‐test, scales, self‐report instrument, quick and simple way, diagnosis, differential diagnosis, therapeutic monitoring
He et al. 2019; Thought control ability moderates the effect of mind wandering on positive affect via the frontoparietal control network	368 participants (103 men, mean age = 19.38 ± 1.35)	To explore the relationships among mind wandering, emotions, and thought control ability, and how thought control ability moderates the effect of mind wandering on positive affect via the frontoparietal control network	Mind wandering, thought control ability (TCA), positive affect (PA), negative emotion (NE), frontoparietal control network (FPCN)	Resting‐state functional magnetic resonance imaging (rsfMRI), Daydreaming Frequency Scale (DDFS), Mind Wandering Frequency Scale (MWFS), Positive Affect and Negative Affect Scale (PANAS), Beck Depression Inventory (BDI), Beck Anxiety Inventory (BAI), Urban Happiness Index Scale (UHIS), Oxford Happiness Inventory (OHI), Center for Epidemiological Studies‐Depression Scale (CES‐D), Thought Control Ability Questionnaire (TCAQ), Siemens 3T Trio scanner, 12‐channel head coil, Institutional Review Board approval, informed consent
Kornacka et al. 2023; Maladaptive task‐unrelated thoughts: self‐control failure or avoidant behavior? Preliminary evidence from an experience sampling study	49 participants (mean age = 30.73, SD = 5.82, 38.8% women)	To test how self‐reported control over task‐unrelated thoughts (TUT) and task valence moderate the link between task difficulty and TUT intensity	Task‐unrelated thoughts (TUT), self‐control, avoidant behavior, experience sampling, emotion regulation	Experience sampling study, MovisensXS application, compliance rates, Daydreaming Frequency Scale (DDFS), Perseverative Thinking Questionnaire (PTQ), Emotion Beliefs Questionnaire, TUT intensity, control over thoughts, task valence, visual analog scale (VAS), momentary assessments, community sample, online trait questionnaires, Polish version of DDFS, transdiagnostic RNT perspective, general beliefs on emotions, usefulness, controllability, context‐task characteristics, task negative valence, interaction, quantitative evidence, mental break, cognitive performance
Arabacı and Parris 2017; Probe‐caught spontaneous and deliberate mind wandering in relation to self‐reported inattentive, hyperactive and impulsive traits in adults	80 undiagnosed individuals (30 male, 50 female, aged 18–37, M = 24.46, SD = 0.50)	To examine the relationship between mind wandering and the core symptoms of ADHD: inattention, hyperactivity, and impulsivity	Mind wandering, ADHD traits, inattention, hyperactivity, impulsivity, deliberate mind wandering, spontaneous mind wandering	Connors’ Adult ADHD Rating Scale: Short Version (CAARS‐S:S), Sustained Attention to Response Task (SART), standard and easy versions, cognitive task difficulty, task demands, button press response, experimental trials, random selection of digits (1–9), withheld response for digit 3, opportunity sample, Bournemouth University, research participation system, advertisement,
				inclusion criteria, ethics committee approval, consent form, Bayes factors, data sensitivity, Helsinki Declaration, British Psychological Society, analyses
Kandeger et al. 2023; Excessive mind wandering, rumination, and mindfulness mediate the relationship between ADHD symptoms and anxiety and depression in adults with ADHD	159 adults diagnosed with ADHD	To investigate the mediating role of excessive mind wandering (EMW), rumination, and trait mindfulness between ADHD symptoms and the severity of anxiety/depression in adults with ADHD	ADHD symptoms, anxiety, depression, excessive mind wandering (EMW), rumination, trait mindfulness	Sociodemographic form, Adult ADHD Severity Rating Scale (ASRS), Hospital Anxiety Depression Scale (HADS), Mind Excessively Wandering Scale (MEWS), Ruminative Response Scale (RSS), Freiburg Mindfulness Inventory (FMI), initial diagnostic examination (SCID‐5), exclusion criteria, cross‐sectional design, self‐report measures, clinical population, mediation analysis, Selçuk University Faculty of Medicine Local Ethics Committee approval
Podda et al. 2022; Mind wandering in people with multiple sclerosis: a psychometric study	170 participants with multiple sclerosis (PwMS)	To assess structural and construct validity and reliability of a brief Italian version of the Mind Wandering (MW) Scale in people with Multiple Sclerosis (PwMS)	Mind wandering, Multiple Sclerosis (PwMS), spontaneous mind wandering (MW‐S), deliberate mind wandering (MW‐D), structural validity, construct validity, reliability	Explorative factor analysis (EFA), mood (Hospital Anxiety Depression Scale), personality (10‐items Big Five Inventory Test), Cronbach's *α* for internal consistency, intraclass correlation coefficients, structural and construct validity, demographic and clinical information, McDonald's criteria, Expanded Disability Status Scale (EDSS), written informed consent, Regional Ethics Committee approval, data collection (December 2020 to March 2021), Italian MS Society (AISM) Rehabilitation Services (Genoa, Padua, Vicenza)
Seli et al. 2017; Cognitive aging and the distinction between intentional and unintentional mind wandering	795 individuals (mean age 37.03; 437 females) who completed a Human Intelligence Task (HIT) on Amazon Mechanical Turk	To reexamine the association between age and mind wandering while distinguishing between intentional (deliberate) and unintentional (spontaneous) mind wandering	Mind wandering, cognitive aging, intentional mind wandering (MW‐D), unintentional mind wandering (MW‐S), executive functioning, task motivation	Mind Wandering: Deliberate (MW‐D) scale, Mind Wandering: Spontaneous (MW‐S) scale, four‐item scales, seven‐point Likert scale, brief demographic and mind wandering questionnaires, Amazon Mechanical Turk, Human Intelligence Task (HIT), ethics committee approval, informed consent, payment ($0.50 US dollars), data collection, sample size, University of Waterloo, guidelines, analyses, age, sex, trait levels, executive functioning, task engagement
Ralph et al. 2013; Media multitasking and failures of attention in everyday life	202 undergraduate students (146 female) from the University of Waterloo	To examine the relations between media multitasking and three aspects of everyday attention: (1) failures of attention and cognitive errors, (2) mind wandering, and (3) attentional control	Media multitasking, attention failures, cognitive errors, mind wandering, attentional control, attentional switching, distractibility	Online self‐report measures, Media Multitasking Index (MMI), Mindful Attention Awareness Scale‐Lapses Only (MAAS‐LO), Attention‐Related Cognitive Errors Scale (ARCES), Memory Failures Scale (MFS), Spontaneous Mind Wandering Questionnaire (MW‐S), Deliberate Mind Wandering Questionnaire (MW‐D), Attentional Switching Questionnaire (AC‐S), Attentional Distractibility Questionnaire (AC‐D), Media Multitasking Beliefs Questionnaire (MMBQ), University of Waterloo, self‐reported measures, course credit
Weinstein et al. 2017; Are you mind‐wandering, or is your mind on task? The effect of probe framing on mind‐wandering reports	110 undergraduate participants from the University of Massachusetts Lowell	To examine the effect of probe framing on mind wandering reports and its implications for the probe‐caught mind wandering paradigm	Mind wandering, task‐unrelated thoughts, probe framing, response bias, probe‐caught method	Reading task using a transcript of a TED talk on ecology, edited transcript with screenshots, test consisting of 20 cued recall questions, Turning Technology clickers, java‐based API, Powerpoint slides, variable interval, buttons 1 or 2 on the clicker as responses, timestamp, clicker number, session date and time, framing of probes, two Powerpoint slides with different wordings, undergraduate participant pool, credit toward General Psychology course, session‐based assignment to mind wandering or on‐task conditions, random fluctuations in participant sign‐ups and attendance, data collection, analysis, University of Massachusetts Lowell
Piil et al. 2020; Mindfulness passes the stress test: attenuation of behavioral markers of mind wandering during acute stress	48 participants (students and employees at the University of Southern Denmark, mean age = 37.60; SD = 10.88)	To clarify if mindfulness can successfully mediate the relationship between cognitive performance and acute stress	Mindfulness, cognitive performance, acute stress, mind wandering, behavioral markers	Randomized controlled trial, mindfulness group (Headspace), active control group (NeuroNation), Sustained Attention to Response Task (SART), Mindful Attention Awareness Scale (MAAS), Perceived Stress Scale (PSS), Cold Pressor Test (CPT), baseline and postintervention measurements, stress‐reduction study, recruitment through groups and e‐mails, University of Southern Denmark, exclusion criteria, informed consent, ethics committee approval, data collection, analysis
Kane et al. 2021; Individual differences in task‐unrelated thought in university classrooms	851 undergraduate students from 10 psychology classes at two US universities	To investigate what academic traits, attitudes, and habits predict individual differences in task‐unrelated thought (TUT) during lectures, and whether TUT propensity mediates associations between academic individual differences and course outcomes	Task‐unrelated thought (TUT), academic traits, attitudes, habits, course outcomes, final grade, situational interest	Thought probes during lectures, on‐task thought, self‐report questionnaires, academic predictors, classroom media‐multitasking habits, initial interest in course topic, mind wandering propensity, boredom propensity, classroom seating (front, middle, back), online questionnaires (Qualtrics), experience‐sampling probes, situational interest questionnaire, course grades, Universities A and B, informed consent, extra‐credit points, data analysis, demographic information, sample size, attention‐check items, standardized final grades, three‐phase procedure
Shin et al. 2015; Away from home: the brain of the wandering mind as a model for schizophrenia	33 schizophrenia patients (SZ) and 33 matched healthy controls (CNT)	To investigate the relationship between mind wandering frequency and schizophrenia symptoms, and the underlying neural mechanisms	Mind wandering, schizophrenia, self‐experience, positive psychotic symptoms, resting‐state functional magnetic resonance imaging (rsfMRI), functional connectivity	Resting‐state BOLD imaging, 3.0 T scanner (Siemens Magnetom Trio), echoplanar imaging (EPI) sequence, 82 regions of interest, default‐mode, salience, and frontoparietal networks, mind wandering subscale of Imaginal Processing Inventory (IPI), 12‐item mind wandering frequency scale, five‐point Likert scale, clinical measures (Positive and Negative Syndrome Scale—PANSS, Hamilton Rating Scale for Depression—HAM‐D, Hamilton Anxiety Rating Scale—HAM‐A),
				preprocessing (SPM8 package), slice‐timing correction, spatial normalization (Montreal Neurological Institute—MNI space), smoothing, temporal band‐pass filtering, regression of head‐motion parameters, white matter, cerebrospinal fluid, and global signal activity, Institutional Review Board approval, informed consent
Gorgolewski et al. 2013; A correspondence between individual differences in the brain's intrinsic functional architecture and the content and form of self‐generated thoughts	166 participants (102 females; ages 18–60, first quartile: 23.25, median: 39, third quartile: 50.75) from the NKI Enhanced Rockland Sample (eNKI‐RS)	To explore the relationship between self‐generated mental activity and intrinsic neural fluctuations using the New York Cognition Questionnaire (NYC‐Q) and resting‐state fMRI data	Self‐generated thoughts, intrinsic neural fluctuations, resting‐state functional magnetic resonance imaging (rsfMRI), brain activity, individual differences	New York Cognition Questionnaire (NYC‐Q), exploratory factor analysis, dimensions of content (future related, past related, positive, negative, social), dimensions of form (words, images, specificity), resting‐state fMRI (TR = 645 ms; voxel size = 3 mm isotropic, duration = 10 min), fractional amplitude of low frequency fluctuations, regional homogeneity, degree centrality, individual differences analysis, community representative sample, DSM‐IV‐TR Axis 1 diagnoses, Institutional Review Board approval, written informed consent, Nathan Kline Institute, Montclair State University, data sharing initiative (eNKI‐RS), brain dynamics
Marcusson‐Clavertz et al. 2017; The relation of dissociation and mind wandering to unresolved/disorganized attachment: an experience sampling study	45 participants (40 females, five males; Mage = 25.7, SD = 5.0, range 18–40)	To investigate the everyday mentation of individuals with unresolved/disorganized attachment (U/d attachment) and its association with dissociation, mind wandering, and negative affect	Unresolved/disorganized attachment (U/d attachment), dissociation, mind wandering, negative affect, everyday mentation	Berkeley‐Leiden Adult Attachment Questionnaire‐Unresolved (BLAAQ‐U), Adult Attachment Interview (AAI), Childhood Traumatic Events Scale (CTES), Short Imaginal Processes Inventory (SIPI), Survey of Anomalous Experiences, experience sampling program (Barrett and Barrett 2005), personal digital assistants (PDAs; Palm Tungsten T), 5 days of experience sampling, 10 prompts per day, semistructured interview, self‐report questionnaires, trauma screening, inclusion criterion, clinical and nonclinical samples, borderline personality disorder (clinical sample), structured clinical interview for DSM‐IV (SCID‐II), recruitment via advertisements and university courses, psychological study description, data analysis, study approval, Swedish translation and back‐translation, informed consent, demographic information
Hunkin et al. 2020; Evaluating the feasibility of a consumer‐grade wearable EEG headband to aid assessment of state and trait mindfulness	68 adult participants (Mage = 22.66, SDage = 7.35)	To evaluate the measures from a consumer‐grade EEG headband (Muse, InteraXon, Inc.) as novel correlates of state mindfulness during focused attention meditation	Wearable EEG headband (Muse), state mindfulness, trait mindfulness, mind wandering, attention lapses	Task‐based measure of state mindfulness, thought probe measures of subjective mind wandering, 14 days of home practice for a subset of participants, Muse headband metrics (Muse mind wandering, recoveries), subjective state mind wandering (thought probe experience sampling), Breath Counting Task, Mindful Attention/Awareness Scale—Lapses
				Only (MAAS‐LO), mindfulness‐related trait measures (Adult Temperament Questionnaire Short Form—Attentional Control subscale, Non‐Attachment Scale—NAS‐7), 2016‐model Muse MU‐02 EEG headband, Muse app, Apple iPad tablets, audio muted during lab tasks, background soundscapes, feedback suppression, attention lapses, attentional control, nonattachment, decentering, data analysis
Scheibner et al. 2017; Internal and external attention and the default mode network	20 healthy, meditation‐naive participants (15 females, mean age = 30, SD = 10, range 18–57)	To clarify the relationship between focused attention meditation and activity in the default mode network (DMN) by distinguishing internal and external attention	Focused attention meditation, internal attention, external attention, default mode network (DMN), mindful attention, mind wandering, refocusing	Thought‐probe paradigm, external (mindfulness of sound) and internal (mindfulness of breathing) attention meditation, home practice (4 consecutive days), fMRI scanning (3 T MRI Scanner, MAGNETOM Trio, TIM‐Technology), four runs alternating between internal and external attention, pseudorandom intervals for thought probes, brain regions (medial prefrontal cortex, posterior cingulate cortex, left temporoparietal junction, left inferior frontal gyrus), reduced DMN activity, ethical approval, informed consent, compensation
Ros et al. 2013; Mind over chatter: plastic up‐regulation of the fMRI salience network directly after EEG neurofeedback	34 right‐handed participants (mean age: 32.6, SD: 10.7, 24 women, 10 men)	To examine whether functional connectivity of distinct fMRI networks would be plastically altered after a 30‐min session of voluntary reduction of alpha rhythm compared to a sham‐feedback condition	EEG neurofeedback (NFB), salience network, functional connectivity, alpha rhythm, cognitive control, brain plasticity	Randomized controlled trial, EEG‐neurofeedback (NFB) group (*n* = 17), sham‐neurofeedback (SHAM) group (*n* = 17), fMRI scanning before and after neurofeedback, structured SCID‐I Interview, neurological or psychiatric disorder screening, randomization, auditory oddball fMRI task, Spielberger's State Anxiety Inventory, Thayer's Activation–Deactivation Checklist, EEG recording, MRI data acquisition, localizer and anatomical scan, sound attenuating MRI‐compatible headphones, offline analysis, veridical feedback, sham‐feedback, experimental protocol, written informed consent, Western Ontario, Canada
Miller 2021; Scene meaningfulness guides eye movements even during mind‐wandering	57 undergraduate students (mean age = 18.84, SD = 0.79, 64% female) from the University of Michigan	To investigate how meaning and visual salience account for fixation allocation during mind wandering compared to on‐task viewing	Mind wandering, visual salience, semantic richness, fixation allocation, meaningful regions, on‐task viewing	Thought probes, real‐world scenes, memory test, salience maps (Graph‐Based Visual Saliency), meaning maps (Henderson and Hayes), eye‐tracking, 5‐point calibration, study–test structure, randomized trial order, thought probe experience sampling (Seli et al. 2016), intentional and unintentional mind wandering, course credits, informed consent, data analysis, University of Michigan
Wilson et al. 2015; Spider stimuli improve response inhibition | experimental	67 undergraduate students (39 females, 28 males, age range 17–42 years, M = 21.7 years, SD = 5.0) from the University of Canterbury	To investigate whether task‐relevant anxiety could improve people's ability to withhold responses in a response inhibition task	Task‐relevant anxiety, response inhibition, Sustained Attention to Response Task (SART), motor response inhibition,	Modified and unmodified versions of SART, Go stimuli, No‐Go targets, probability of occurrence, 225 trials, stimulus presentation (250 ms), mask (900 ms), response window
	in Christchurch, New Zealand		mind wandering, speed‐accuracy trade‐off, task‐unrelated thoughts	(1100 ms), picture SART (spiders and neutral objects/scenes from Geneva Affective Picture Database—GAPED), arousal and valence ratings, four DSSQ subscales (energetic arousal, tense arousal, task‐related thoughts, task‐unrelated thoughts),
Perry et al. 2022; How chanting relates to cognitive function, altered states and quality of life	456 English speaking participants from 32 countries who regularly chant	To examine how chanting relates to cognitive function, altered states, and quality of life across various traditions	Chanting, cognitive function, altered states, quality of life, flow states, mystical experiences, mindfulness, mind wandering	Global survey, psychometric scales (Mindful Attention Awareness Scale—MAAS, Mind Wandering Questionnaire—MWQ), flow states, mystical experiences, engagement (experience, practice duration, regularity), intentionality (devotion, intention, sound), quality of life, call and response chanting, repetitive prayer, social media recruitment, community newsletters, online chanting forums, prize draw, Macquarie University Ethics committee approval, informed consent, data analysis
Belardi et al. 2024; Effects of 5 Hz auditory beat stimulation on mind wandering and sustained attention in an online experiment	541 participants (217 English‐speaking, 324 German‐speaking)	To explore auditory beat stimulation (ABS) to reduce mind wandering and increase sustained attention	Mind wandering (MW), auditory beat stimulation (ABS), sustained attention, Sustained Attention to Response Task (SART), experience sampling, meta‐awareness	Binaural or monaural presentation, pure tones at 437.5 Hz and 442.5 Hz, control conditions (no sound and uniform pure tone at 440 Hz), experimental manipulations (SART interstimulus interval, sequence of SART stimuli, expectancy of a creativity task), SART implementation, experience sampling probes, Mindful Attention Awareness Scale (MAAS), UniDistance Suisse Ethics Commission approval, MTurk recruitment, course credits, exclusion criteria, sensitivity analysis, data analysis, SART % NOGO success, minimal detectable effect size, G*Power software, reliability and validity of MAAS, Unusual Uses Task (UUT), compensation
Zhang et al. 2023; The influence of neuroticism on insomnia: the chain mediating effect of mind wandering and symptom rumination	1790 online participants (804 males, 986 females; average age = 22.07 years, SD = 2.28)	To investigate the relationship between neuroticism and insomnia, and the mediating effect of mind wandering and symptom rumination	Neuroticism, insomnia, mind wandering, symptom rumination, psychological mechanisms	Big Five Personality Inventory (BFI‐10), Athens Insomnia Scale (AIS), Chinese version of the Mind Wandering Questionnaire (MWQ), Chinese version of the Nolen‐Hoeksema Ruminative Responses Scale (RRS), online survey (www.wjx.cn), social media recruitment (WeChat, Bilibili, Weibo, QQ), attention check questions
Babo‐Rebelo et al. 2016; Neural responses to heartbeats in the default network encode the self in spontaneous thoughts	20 right‐handed volunteers (mean age: 24.1 years, eight male)	To investigate the link between selfhood and neural responses to heartbeats in the default network (DN)	Default network (DN), self‐related cognition, bodily state monitoring, autonomic regulation, neural monitoring of internal organs, selfhood, heartbeats, mind wandering	Magnetoencephalography (MEG), neural responses to heartbeats, thought‐sampling task, self‐relatedness of interrupted thought, visual stimulus, ventral precuneus, ventromedial prefrontal cortex, peripheral autonomic measures, pilot participants, training procedure, ratings of spontaneous thoughts, ethical approval, informed
				consent, data analysis, CPP Ile de France III
Li et al. 2024; Big five personality and mind wandering in athletes: mediating role of trait anxiety	681 athletes (350 male, 331 female; average age 19.44 years; average years of sports experience 6.17 years) from various provinces in China	To clarify which personality type is more prone to mind wandering and the mediating role of trait anxiety in athletes	Big Five personality traits (extraversion, agreeableness, conscientiousness, neuroticism, openness), mind wandering, trait anxiety, sports performance	Athlete Mind Wandering Scale (five dimensions: weak attentional control, spontaneous thinking, psychological gap, competition mood, somatic sensation), Chinese adjectives scale of Big‐Five factor personality short scale version, Pre‐Competition Emotion Scale‐Trait (four dimensions: individual failure anxiety, social expectancy anxiety, somatic anxiety, trait confidence)
Shinagawa et al. 2023; Coexistence of thought types as an attentional state during a sustained attention task	31 university students (12 males, 19 females; mean age = 21.4, SD = 1.43; range = 19–25 years) from Keio University	To examine whether coexistence of thought types occurred during a sustained attention task	Thought types (task‐focus, mind wandering, task‐related, external stimuli‐related), attentional state, sustained attention task (SART), thought probes, immersion level	Thought probes, random intervals, five‐point scale (0%–100% by 25%), task‐focused thoughts, task‐related thoughts, external stimuli‐related thoughts, task‐unrelated thoughts (TUTs), self‐reports, behavioral indexes, hidden Markov model
Lübber 2020; Motivational based performance trade‐off in sequential tasks and the role of mind‐wandering	146 participants from Wake Forest University, reduced to 107 valid participants after exclusions	To investigate the motivational‐based trade‐off in performances between two sequential tasks (SART and name listing task), mediated by mind wandering	Mind wandering, performance trade‐off, motivation, sequential tasks, SART, name listing task	Multilevel structural equation model, repeated blocks design (seven blocks)
Sharma et al. 2020; Indian classical music with incremental variation in tempo and octave promotes better anxiety reduction and controlled mind wandering—a randomised controlled EEG study	21 male undergraduate medical students (11 in Varying Music group, 10 in Stable Music group)	To examine the anxiolytic effect of incremental variations in tempo and octave in Carnatic classical music	Anxiety reduction, mind wandering, incremental variations in tempo and octave, Carnatic classical music, EEG, HRV	Varying Music (VM) group: instrumental music with incremental variations in tempo and octave, Stable Music (SM) group: instrumental music without variations, control: silence, EEG and ECG recordings, Beck's Anxiety Inventory (BAI), State‐Trait Anxiety Inventory (STAI), bilateral temporo–parieto–occipital regions, lower frequency EEG power, higher frequencies, default mode network (DMN) activity, alpha/beta asymmetry, midline power reduction, heart rate variability (HRV)
Wong and Yu 2024; Left superior parietal lobe mediates the link between spontaneous mind‐wandering tendency and task‐switching performance	173 native German‐speaking adults (80 females; age range: 20–80 years)	To investigate the association between spontaneous mind wandering tendency, task‐switching performance, structural connectivity, and resting‐state functional connectivity	Spontaneous mind wandering, task‐switching performance, structural connectivity, resting‐state functional connectivity, left superior parietal lobe, dorsal attention network, default mode network (DMN)	Mind wandering scales (MW‐D and MW‐S), emotional switching task, cortical thickness analysis, resting‐state functional MRI (rs‐fMRI), 3 T Siemens Magnetom Verio Scanner, negative dorsal attention network–DMN functional connectivity, multimodal MRI, state and trait phenotypic assessments, excessive head motion
Irving et al. 2020; What does “mind‐wandering” mean to the folk? An empirical investigation	Study 1: 364 participants (Gender: 210 men, 153 women, one other; Median age group: 24–35), Study 2: 182 participants (Gender: 98 men, 82 women, two others; Median age group: 24–35)	To investigate the ordinary understanding of the term “mind wandering” and test the four major accounts of mind wandering	Task‐unrelated thought, stimulus‐independent thought, unintentional thought, dynamically unguided thought, folk judgments	Study 1: Vignettes varying with respect to task‐relatedness, intentionality; Study 2: Vignettes varying with respect to dynamic guidance and stimulus‐dependence, four major accounts of mind wandering
Zhang et al. 2020; Perceptual coupling and decoupling of the default mode network during mind‐wandering and reading	339 participants (Experiment 1: 29 undergraduate students; Experiment 2: two separate resting‐state samples, one with 244 participants and another with 69 participants)	To understand how the mind wandering state emerges during reading and its neural correlates, particularly focusing on the default mode network (DMN)	Mind wandering, autobiographical memory retrieval, narrative comprehension, default mode network (DMN), functional connectivity, perceptual decoupling, visual input	Experiment 1: Functional magnetic resonance imaging (fMRI), reading expository texts, memory retrieval, DMN regions, left temporal and lateral prefrontal regions, ventral visual cortex; Experiment 2: Resting‐state functional connectivity, intrinsic connectivity of DMN regions, primary visual cortex, mind wandering during reading, reading comprehension, self‐reported measurement of off‐task thoughts, behavioral assessments, neural regions involved in memory and reading
Berthié et al. 2015; The restless mind while driving: drivers’ thoughts behind the wheel	128 drivers (61.7% women, aged between 20 and 68 years, mean age = 36.46 years)	To examine the prevalence and nature of mind wandering (MW) among drivers during their most recent trip	Mind wandering (MW), driving, inattention, road safety, off‐task thoughts, driving performance	Questionnaire (73 questions) covering four sections: (1) General information (biographical and trip's contextual characteristics), (2) Presence of MW (number of off‐task thoughts, percentage of trip spent in MW), (3) Content of thoughts (temporal focus, emotional valence, theme of thoughts), (4) Perception of driving performance (awareness of MW, variation in driving behavior)
Seli et al. 2012; Wandering minds and wavering rhythms: linking mind wandering and behavioral variability	Sample 1 = 41 undergraduates (43 initially, two removed), Sample 2 = 39 undergraduates from the University of Waterloo	To explore the association between mind wandering and behavioral variability in tasks requiring executive control	Mind wandering (tuned‐out and zoned‐out), behavioral variability, task‐related executive control, response variability	Metronome response task (MRT), continuous rhythmic presentation of tones, key presses (spacebar)
Walpola et al. 2020; Mind‐wandering in Parkinson's disease hallucinations reflects primary visual and default network coupling	38 Parkinson's disease patients (18 with hallucinations, 20 without) and 40 controls (a subset of 20 also underwent neuroimaging)	To explore the association between mind wandering and visual hallucinations in Parkinson's disease, and their relationship with brain network coupling	Mind wandering, visual hallucinations, Parkinson's disease, brain network coupling, primary visual cortex, default mode network (DMN)	Thought‐sampling task, validated for use in patient populations with cognitive impairment, 9 trials with two‐dimensional colored shapes (varying durations: Short: 20 s, Medium: 30–60 s, Long: ≥ 90 s), resting‐state functional magnetic resonance imaging (fMRI), internetwork connectivity, seed‐to‐voxel analyses, Montreal Cognitive Assessment (MoCA), motor severity (Hoehn and Yahr Scale, MDS UPDRS‐III), mood (Beck Depression Inventory‐II), general neuropsychological measures (working memory, attentional set‐shifting, memory), neuroimaging, dopaminergic dose equivalence (DDE) scores, control group (age‐ and education‐matched, screened for neurological or psychiatric disorders), ethical approval, written informed consent, Declaration of Helsinki, local Ethics Committees, data access procedures
Kim and Lee 2022; Spectral dynamic causal modeling of mindfulness, mind‐wandering, and resting‐state in the triple network using fMRI	59 right‐handed, healthy volunteers (all males, age = 25.1 ± 2.9 years, mean ± SD)	To compare effective connectivity patterns across mindfulness, mind wandering, and resting‐state conditions using the triple network	Mindfulness, mind wandering, resting‐state, triple network (salience network, default mode network—DMN, central executive network—CEN), functional connectivity, effective connectivity	Spectral dynamic causal modeling, functional MRI (fMRI) data
Marcusson‐Clavertz and Kjell 2019; Psychometric properties of the spontaneous and deliberate mind wandering scales	Study 1: 284 participants (139 males, 145 females), Study 2: 323 participants (details not provided)	To investigate the psychometric properties of the Spontaneous and Deliberate Mind Wandering Scales (SDMWS)	Spontaneous mind wandering, deliberate mind wandering, psychometric properties, stability, relations to other psychological variables, test–retest reliability	Two studies: Study 1 ‐ evaluated stability of SDMWS over 2 weeks, Study 2 ‐ evaluated relations to Generalized anxiety disorder symptoms, Openness, Social desirability, experience‐sampling reports of mind wandering; measures: Spontaneous and Deliberate Mind Wandering Scales (SDMWS), Short Imaginal Processes Inventory (SIPI), Social Desirability Scale (SDS)
Mrazek et al. 2013b; Young and restless: validation of the Mind‐Wandering Questionnaire (MWQ) reveals disruptive impact of mind‐wandering for youth	Study 1: 663 undergraduates (262 males, 401 females); Study 2: 77 undergraduate students (26 males, 51 females); Study 3: 106 high school students (all female); Study 4: 78 middle school students (35 males, 43 females)	To validate the Mind‐Wandering Questionnaire (MWQ) across different age groups and examine the impact of mind wandering on performance and well‐being among youth	Mind wandering, task‐unrelated thought, reading comprehension, mood, life‐satisfaction, stress, self‐esteem	Study 1: Developed MWQ, assessed internal consistency; Study 2: Examined convergent validity with thought sampling during a task, operation span task (OSPAN); Study 3: Assessed mind wandering and well‐being in high school students, reading comprehension test with thought sampling, MWQ, MAAS, SWLS, PSS, PANAS, RSES; Study 4: Extended MWQ to middle school students, same experimental procedure as Study 3; thought probes during reading tests
Demanet et al. 2013; Biasing free choices: the role of the rostral cingulate zone in intentional control	25 healthy subjects (five males, age range: 19–25 years, mean age = 21.4 years) from Ghent University	To investigate the role of the rostral cingulate zone (RCZ) in intentional control and how free choices are biased by past experiences	Intentional control, free choices, medial frontal cortex, rostral cingulate zone (RCZ), biased choices, mind wandering	Functional magnetic resonance imaging (fMRI)
Kang et al. 2014; Pupil dilation dynamics track attention to high‐level information	22 Dartmouth undergraduates (13 females), 16 passed quality control (11 females)	To investigate whether pupil dilations reflect online cognitive processing beyond sensory gain during attention	Pupil dilation, attention, mind wandering, online cognitive processing, high‐level information, sensory gain	Choice Reaction Time (CR) task, Working Memory (WM) task, eye‐tracking (ASL Eye‐Trac 6 eye‐tracker, 120 Hz), pupil diameter collection
Hart et al. 2022; Task‐unrelated thought increases after consumption of COVID‐19 and general news	Study 1: 58 participants (average age = 29.97 years, 48 identified as female, 36 identified as white, 15 identified as Asian), Study 2: 66 participants (average age = 19.68 years, 62 identified as female, 26 identified as white, 28 identified as Asian)	To examine the impact of news consumption on task‐unrelated thoughts (TUTs) in daily life	Task‐unrelated thoughts (TUTs), news consumption, COVID‐19 news, general news, Ecological Momentary Assessment (EMA), productivity, safety, personally salient concerns	EMA surveys throughout the day for 10 days, regression models
Girardeau et al. 2023; The benefits of mind wandering on a naturalistic prospective memory task	60 participants (mean age: 22.08 ± 4.76 years) recruited through Université Paris Cité intranet or RISC	To investigate the role of mind wandering (MW) in prospective memory (PM) performance, specifically in naturalistic settings	Mind wandering (MW), prospective memory (PM), retrospective PM, prospective PM, cognitive load, event‐based (EB) PM, time‐based (TB) PM	Virtual reality (VR) immersive walk in a town, Dell monitor, HTC Vive PRO set, Unity software, virtual environment resembling Paris, MW manipulation (high vs. low cognitive load), spontaneous past‐oriented MW, spontaneous future‐oriented MW, voluntary future‐oriented MW, Neuropsydia 1.0.581, episodic future thinking (EFT), episodic past thinking (EPT), *n*‐back task (1‐back, 3‐back), thought probes, classification task, audio tapes, random assignment, stimulus presentation, data analysis, VR interaction, twofold functional role of MW (consolidate intention
Gao et al. 2024; Longitudinal associations between metacognition and spontaneous and deliberate mind wandering during early adolescence	4302 Chinese students (47.4% female; initial Mage = 9.84, SDage = 0.47	To examine the developmental trajectories of spontaneous and deliberate mind wandering and their dynamic associations with metacognition during early adolescence	Metacognition, spontaneous mind wandering, deliberate mind wandering, developmental trajectories, early adolescence	Five assessment waves over 2.5 years (from Grade 4 to Grade 6), questionnaires, Mind Wandering: Deliberate (MW/D) and Mind Wandering: Spontaneous (MW/S) scales
Ford et al. 2016; Using concurrent EEG and fMRI to probe the state of the brain in schizophrenia	30 schizophrenia patients (24 with DSM‐IV schizophrenia, six with schizoaffective disorder) and 23 healthy comparison subjects (HC)	To explore when and where auditory perception is affected by schizophrenia using EEG and fMRI data	Auditory perception, schizophrenia, EEG‐derived event‐related potentials (ERPs), functional magnetic resonance imaging (fMRI), joint independent components analysis (jICA)	EEG and fMRI data acquisition, ERP‐fMRI joint independent components (JIC), N100 JIC, P200 JIC, temporal weights, fMRI spatial weights, superior and middle temporal gyri (STG/MTG), frontal areas, default mode network (DMN), visual cortex
Arnau et al. 2019; Inter‐trial alpha power indicates mind wandering	100 participants recruited via local newspaper advertisement, social media, and flyers in Heidelberg	To clarify the electrophysiological correlates of mind wandering using EEG and examining the temporal dynamics of mind wandering	Mind wandering, task performance, intertrial alpha power, internally oriented state of attention	Switching task, thought probes, response accuracy, alpha power measurements during intertrial intervals, cluster‐based permutation approach, EEG recording
Chou et al. 2020; Transcranial direct current stimulation of default mode network parietal nodes decreases negative mind‐wandering about the past	90 participants recruited from the community (exclusion criteria applied)	To assess changes in maladaptive mind wandering following criticism using transcranial direct current stimulation (tDCS)	Mind wandering, negative thoughts about the past, default mode network (DMN), transcranial direct current stimulation (tDCS), bilateral inferior parietal lobe (IPL) nodes, cognitive modulation	Participants received either excitatory, inhibitory, or sham tDCS, changes in mind wandering frequency and content assessed, cathodal stimulation effects
McVay and Kane 2013; Dispatching the wandering mind? Toward a laboratory method for cuing “spontaneous” off‐task thought	Undergraduates at the University of North Carolina at Greensboro (UNCG), tested in groups of 1–6 (aiming for *N* = 60 per experiment)	To develop a method for inducing mind wandering episodes on demand in response to task‐embedded cues	Mind wandering, spontaneous off‐task thought, task‐embedded cues, correlational and observational methods	go/no‐go task, thought probes, four questionnaires (Cognitive Failures Questionnaire—Memory and Attention Lapses, Personal Concerns Inventory, AD/HD Rating Scale, Action Orientation Scale)
Wereszczyński and Niedźwieńska 2022; Deficits in spontaneous and stimulus‐dependent retrieval as an early sign of abnormal aging	27 healthy older adults and 27 individuals with amnestic Mild Cognitive Impairment (aMCI)	To provide evidence supporting the Spontaneous Retrieval Deficit hypothesis in preclinical stages of dementia	Mind wandering, spontaneous retrieval, stimulus‐dependent retrieval, episodic memory, amnestic Mild Cognitive Impairment (aMCI), Alzheimer's disease	Comparison of mind wandering between aMCI individuals and healthy controls (HC), task with exposure to highly meaningful or unmeaningful pictures, reduction in mind wandering among aMCI individuals with highly meaningful stimuli, pronounced reduction in past‐oriented thoughts, involuntary autobiographical memories, neuropsychological evaluation (HVLT, CVLT, ACE‐III)
Smith et al. 2018; Mind‐wandering rates fluctuate across the day: evidence from an experience‐sampling study	144 participants recruited from a large public Canadian university, compensated with class credit	To examine diurnal fluctuations in mind wandering rates using everyday experience sampling	Mind wandering, diurnal fluctuations, experience sampling, free movement of thought, task‐unrelated thought, stimulus‐independent thought	Study 1: Self‐reportsStudy 2: Replication with reanalysis of past data, differences in parameter values for models representing freely moving thought, task‐unrelated thought, and stimulus‐independent thought, UBC Behavioral Research
Martarelli et al. 2020; A personality trait‐based network of boredom, spontaneous and deliberate mind‐wandering	German‐speaking sample (*n* = 418) and English‐speaking sample (*n* = 364)	To translate and validate the Spontaneous and Deliberate Mind‐Wandering Scales (SDMWS) and the Short Boredom Proneness Scale (SBPS) into German, and explore their relationships	Mind wandering (spontaneous and deliberate), boredom, personality traits, measurement invariance, confirmatory factor analyses (CFA), exploratory graph analysis (EGA)	Translation procedure (forward‐ and back‐translation by independent translators), confirmatory factor analyses, measurement invariance across samples, spontaneous and deliberate mind wandering scales (SDMWS), short boredom proneness scale (SBPS), open‐mindedness scale (BFI‐2‐S), data analysis
Zuberer et al. 2021; Integration and segregation across large‐scale intrinsic brain networks as a marker of sustained attention and task‐unrelated thought	29 healthy, right‐handed adults (13 males, 16 females; mean age = 26.7 ± 3.9)	To investigate brain activity during a cognitive task, focusing on sustained attention and task‐unrelated thought	Sustained attention, task‐unrelated thought, large‐scale intrinsic brain networks, information processing, integration, segregation, mind wandering	Functional magnetic resonance imaging (fMRI), cognitive task, motor responses, subjective reports, global attributes of communication, integration and segregation of information processing across large‐scale networks, optimal and suboptimal attention states, auditory and sensorimotor systems, intrinsic neural fluctuations, neural markers
Carciofo et al. 2017; Metacognitive beliefs mediate the relationship between mind wandering and negative affect	Study 1: 254 participants (aged 18–28), Study 2: 130 participants (aged 18–28)	To investigate associations between mind wandering, metacognitive beliefs, and their relationship with negative affect	Mind wandering, metacognitive beliefs, negative affect, daydreaming, mindfulness, sleep quality	Questionnaire measures, MetaCognitions Questionnaire (MCQ‐30), Imaginal Processes Inventory (Daydreaming Frequency, Mind Wandering, Problem‐Solving Daydreams scales), Mindful Attention Awareness Scale‐Lapses Only (MLO), Pittsburgh Sleep Quality Index (PSQI), Positive and Negative Affect Schedule (PANAS), Sustained Attention to Response Task (SART) with thought‐probe assessment
Xu et al. 2014; Nondirective meditation activates default mode network and areas associated with memory retrieval and emotional processing	14 experienced practitioners of Acem meditation (eight men, six women, 13 right‐handed, aged 28–61 years)	To assess brain activity during nondirective meditation and compare it to concentrative practicing and rest	Nondirective meditation, concentrative practicing, mind wandering, default mode network (DMN), episodic memories, emotional processing	Functional magnetic resonance imaging (fMRI), two experimental conditions (nondirective meditation vs. rest, nondirective meditation vs. concentrative practicing)
Slepian et al. 2017; The experience of secrecy	Study 1: 200 participants (Mage = 34.24 years, SD = 11.39; 63% female), Study 2: 200 participants (Mage = 33.03 years, SD = 10.34; 53% female), Study 3: 200 participants (Mage = 33.72 years, SD = 10.47; 62% female)	To explore the secrets people have, the experience of having a secret, and the relationship between secrecy and well‐being	Secrecy, mind wandering, well‐being, relationship satisfaction, authenticity, physical health	Ten studies, in‐person recruitment of tourists in a major metropolitan area, online samples (Amazon's Mechanical Turk—MTurk), more diverse and representative of the US population than undergraduate samples, anonymity in recalling secrets, Commons Secrets Questionnaire (CSQ), list of categories of secrets, analysis of more than 13,000 secrets, recall tasks, longitudinal designs, outcomes for well‐being, relationship satisfaction, authenticity, physical health, harmful effects of spontaneously thinking about secrets,
Zhao et al. 2023; The relationship between schizotypal personality features and mind wandering among college students during COVID‐19 pandemic: a moderator of depression	Study 1: 153 Chinese college students, Study 2: 557 college students	To examine changes in schizotypal personality features, and the relationship between schizotypal personality features, mind wandering, and depression during the COVID‐19 pandemic	Schizotypal personality features, mind wandering, depression, COVID‐19 pandemic, moderator effect	Study 1: Longitudinal tracking of schizotypal personality features using the Schizotypal Personality Questionnaire (SPQ; Study 2: Relationship between schizotypal personality features, mind wandering, and depression assessed using SPQ, Beck Depression Inventory, and Mind‐Wandering Questionnaire
Ottaviani et al. 2015; Cognitive rigidity is mirrored by autonomic inflexibility in daily life perseverative cognition	42 participants (19 men, 23 women; mean ages 26.9 and 26.5 years, respectively)	To investigate the relationship between cognitive rigidity, perseverative cognition (PC), and mind wandering (MW)	Mind wandering (MW), perseverative cognition (PC), cognitive rigidity, attentional, behavioral, affective, autonomic manifestations, heart rate (HR), heart rate variability (HRV)	Ambulatory HR and HRV recording for 24 h, ongoing thoughts and moods reported using electronic diaries every 30 min, MW characterized by less effort to inhibit thought, less interference with activities, higher HRV, absence of mood worsening, PC characterized by rigid and defensive patterns,
				worse sleep quality predicted by higher levels of trait rumination and daily PC, participants invited via study announcement
Jin et al. 2019b; Distinguishing vigilance decrement and low task demands from mind‐wandering: a machine learning analysis of EEG	30 participants (16 females, aged 18–31 years, M = 23.73, SD = 3.47)	To investigate whether mind wandering is dependent on vigilance and task demands, or is an independent phenomenon	Mind wandering, vigilance, task demands, support vector machine (SVM) classifiers, self‐reported mind wandering, EEG data, thought probes	Training SVM classifiers on EEG data under low and high vigilance conditions, low and high task demands, testing classifiers on self‐reported mind wandering, intermittent thought probes, dipole fitting for source localization of neural correlates, neural structures, distinct phenomena, value of machine‐learning classifiers, continuous EEG recording, visual search task, Sustained Attention to Response Task (SART
Conrad and Newman 2021; Measuring mind wandering during online lectures assessed with EEG	52 students (36 women, 16 men, aged 17–28 years; M = 20.6, SD = 2.5)	To explore the relationship between mind wandering and online education using electroencephalography (EEG)	Mind wandering, online education, EEG, electroencephalography, spectral power, event‐related potentials (ERP), attention, task‐unrelated thoughts	a 75‐min educational video lecture, task‐irrelevant auditory tones, comparison of EEG spectral power and ERP between high and low degrees of self‐reported mind wandering
Welz et al. 2018; Happy thoughts: mind wandering affects mood in daily life	43 university students (aged 19–32 years; 61% women)	To examine the prospective effects of mind wandering (MW) on mood in daily life and possible moderating effects of dispositional mindfulness and rumination	Mind wandering, mood, positive affect, negative affect, dispositional mindfulness, rumination, electronic ambulatory assessment	Participants filled out questionnaires on trait mindfulness and rumination, 5 days of electronic ambulatory assessment of MW and affect ten times a day, prospective models
Mendes et al. 2017; A functional connectome phenotyping dataset including cognitive state and personality measures	194 native German‐speaking participants (94 females, mean age = 34 years, median age = 27, SD = 16 years)	To explore higher‐order cognitive faculties, self‐generated mental experience, and personality features in relation to the intrinsic functional architecture of the brain	Mind wandering, personality traits, cognitive abilities, state and trait phenotypic assessments, multimodal magnetic resonance imaging (MRI), resting‐state functional MRI (rs‐fMRI), 3 Tesla Siemens Magnetom Verio scanner, multiband EPI sequence, 3D MP2RAGE sequence	Participants filled out 31 questionnaires, performed 7 tasks, reported 4 probes of in‐scanner mind wandering
Rahl et al. 2017; Brief mindfulness meditation training reduces mind‐wandering: the critical role of acceptance	147 participants (aged 18–30 years, 74 male)	To evaluate whether the attention monitoring component or the acceptance training component of mindfulness training reduces mind wandering	Mind wandering, mindfulness, acceptance, attention monitoring, meditation, emotion regulation	Participants randomized to one of four conditions (attention monitoring + acceptance mindfulness training, attention monitoring only mindfulness training, relaxation training, reading control), 3‐day brief mindfulness training, pretraining measures of dispositional mindfulness and treatment expectancies, post‐training Sustained Attention Response Task (SART) measuring mind wandering, lowest mind wandering observed in attention monitoring + acceptance condition
Tarailis et al. 2022; The resting‐state paradigm is frequently applied to study spontaneous activity of the brain in normal and clinical conditions	226 young healthy participants (*F* = 131, M = 95, age 23.41, ± 3.87)	To investigate the relationship between brain activity and subjective experiences during the resting‐state using Global Field Synchronization (GFS)	Spontaneous thought, resting‐state, Global Field Synchronization (GFS), functional connectivity, EEG, Amsterdam Resting State Questionnaire (ARSQ)	Reanalysis of resting‐state data, ARSQ domain of Comfort, strong evidence of relationship between Comfort and GFS values in alpha range (*r* = 0.210, BF10 = 12.338) and beta frequency range (*r* = 0.196, BF10 = 6.307), assessments of spontaneous
				thought during resting‐state, intrinsic electrical brain activity
Bozhilova et al. 2020; Electrophysiological correlates of spontaneous mind wandering in attention‐deficit/hyperactivity disorder	69 adults with ADHD and 29 controls, sex‐ and age‐matched	To investigate cognitive‐EEG markers associated with increased spontaneous mind wandering (MW‐S) in ADHD	Spontaneous mind wandering (MW‐S), ADHD, cognitive‐EEG markers, attention‐deficit/hyperactivity disorder, EEG, P3, time‐frequency brain‐oscillations	Sustained Attention to Response Task (SART), task performance, EEG measures (P3, event‐related alpha and beta suppression, theta power activations), self‐report questionnaire of MW‐S, hierarchical regression model, comparisons between groups, data analysis
Sekiguchi 2023; Curiosity makes your mind wander: effects of epistemic curiosity and trait anxiety on mind wandering	260 graduate and undergraduate students from Tokyo Gakugei University (162 female, 96 male, two undisclosed)	To examine how epistemic curiosity and trait anxiety predict mind wandering (MW) tendency	Mind wandering (MW) tendency, epistemic curiosity, trait anxiety, executive control, intentional MW, unintentional MW	Self‐report questionnaire, thought probes during behavioral task, Epistemic Curiosity Scale (diversive and specific curiosity), State‐Trait Anxiety Inventory
Latinjak 2018; Athletes’ self‐reports on mind wandering while practicing sports: an exploratory two‐study project	Study 1: 94 athletes (Mage = 19.51, SD = 1.65), Study 2: 115 athletes (Mage = 22.82, SD = 3.61)	To describe athletes’ experiences with mind wandering (MW) during sports practice, and explore its frequency, effects, and control	Mind wandering (MW), sports, frequency, effects, control	Study 1: 19‐item questionnaire, test–retest reliability, hierarchical content analysis, informed consent, sports‐related activities, verbal invitations; Study 2: Qualitative descriptions, hierarchical content analysis, descriptive data, informed consent, local sport, life and health science faculties, invited to lectures, situations of mind wandering
Atchley et al. 2017; EEG frequency changes prior to making errors in an easy stroop task	27 older adults (Mage = 60 years, SD = 6)	To assess potential electroencephalographic markers of task‐unrelated thought or mind wandering state related to error rates	Mind wandering, task‐unrelated thought, EEG, alpha activity, theta activity, errors	Computerized Stroop matching task, congruent and incongruent trials, EEG recording of frontal frequency band activity, behavioral data, analysis of 1‐s epochs prior to stimulus presentation, comparison of correct vs. incorrect responses, convenience sample, mindfulness meditation randomized controlled trial, Perceived Stress Scale (PSS), Eprime 2.0, response logging
Zanesco et al. 2021; Self‐reported mind wandering and response time variability differentiate prestimulus electroencephalogram microstate dynamics during a sustained attention task	36 undergraduate students (18 women, Mage = 18.83 years, SDage = 1.28)	To investigate the association between EEG microstate temporal dynamics and self‐reported mind wandering	Mind wandering, EEG microstates, sustained attention, response time variability	Sustained Attention to Response Task with Faces (F‐SART), experience sampling probes, broadband electroencephalography, segmentation into brain electric microstates, data‐driven clustering of topographic voltage patterns, comparison of on‐ vs. off‐task moments in prestimulus epochs, variability in response times
Rummel and Nied 2017; Do drives drive the train of thought?—Effects of hunger and sexual arousal on mind‐wandering behavior	130 participants (Mage = 25, SD = 8; 66% female)	To investigate whether and how hunger and sexual arousal influence mind wandering frequency and content	Mind wandering, hunger, sexual arousal, experience‐sampling, reading task, need‐related off‐task thoughts	Hunger induction, sexual arousal induction, reading task, experience‐sampling method, random moments, thought probes, open‐response format, time perspective selection, space bar presses, War and Peace (Tolstoi 2015)
Orwig et al. 2024; Intentionality of self‐generated thought: contributions of mind wandering to creativity	155 healthy adults from the mind–brain–body dataset (71 females; age range: 20–35)	To distinguish between intentional and unintentional mind wandering and explore their behavioral and neural correlates in relation to creativity	Mind wandering, creativity, intentional mind wandering, unintentional mind wandering, resting‐state network connectivity, divergent thinking task	Resting‐state fMRI scans, self‐report measures of mind wandering (deliberate and spontaneous), Alternative Uses Task (AUT), graph theory analysis, weighted degree connectivity, default network, left temporal pole, topological connectivity differences, data from Open fMRI database, translated
				questionnaires, written informed consent, IRB approval, monetary compensation, data analysis
McVay and Kane 2012; Drifting from slow to “D'oh!”: working memory capacity and mind wandering predict extreme reaction times and executive control errors	288 undergraduates (18–35 years of age)	To test predictions of executive attention and coordinative binding theories of working memory capacity (WMC)	Working memory capacity (WMC), mind wandering, sustained attention, executive control errors, reaction times	WMC screening (OSPAN, SSPAN, RSPAN tasks), Sustained Attention to Response Task (SART), experimental and individual‐differences design, thought‐sampling, quantitative evidence‐accumulation RT model, intrasubject RT patterns
Johannes et al. 2018; Mind‐wandering and mindfulness as mediators of the relation between online vigilance and well‐being	371 participants (students from Radboud University)	To investigate whether online vigilance is related to cognitive consequences in the form of increased mind wandering and decreased mindfulness, and examine the implications for well‐being	Mind wandering, mindfulness, online vigilance, well‐being, Daydreaming Frequency Scale	Online survey hosted by Qualtrics, snowball sampling, course credit, path model estimation, survey on media use and personality, small effect sizes detection, two‐tailed correlation, statistical power analysis, sample size determination, dissemination on Facebook and personal networks, data analysis
Ottaviani et al. 2015; Cognitive rigidity is mirrored by autonomic inflexibility in daily life perseverative cognition	42 participants (19 men, 23 women; mean ages 26.9 and 26.5 years, respectively)	To investigate the relationship between cognitive rigidity, perseverative cognition (PC), and mind wandering (MW)	Mind wandering (MW), perseverative cognition (PC), cognitive rigidity, heart rate (HR), heart rate variability (HRV), electronic diary, trait rumination, trait worry	Ambulatory HR and HRV recording for 24 h, ongoing thoughts and moods reported using electronic diaries every 30 min, exclusionary criteria, recruitment of university students and employees, protocol approval by Bioethical Committee of S. Lucia Foundation, compensation for participation, Android phone diary implementation, random signaling, questionnaires on socio‐demographic and lifestyle factors, SRRS and PSWQ scales
Chaieb et al. 2022b; Modulation of mind wandering using monaural beat stimulation in subjects with high trait‐level mind wandering	107 subjects (mean age 26.2 ± 0.6; 80 female); 34 subjects for experimental part (age 25.7 ± 0.8, 23 female)	To investigate whether mind wandering (MW) can be reduced by monaural theta beats in subjects with high trait‐levels of MW	Mind wandering (MW), monaural beat stimulation, trait‐level MW, sustained attention	Online survey, Self‐Rating Mind Wandering Scale (Mrazek et al. 2013a), selection based on MW scores, experimental paradigm at Department of Epileptology, University Hospital Bonn, Sustained Attention to Response Task (SART), thought‐probes, meta‐awareness, temporal orientation of MW, auditory beat stimulation conditions, experience sampling
Tang and Li 2024; EEG complexity measures for detecting mind wandering during video‑based learning	28 participants (average age 22.8 years; 14 males, 14 females)	To explore the efficacy of various EEG complexity measures in detecting mind wandering during video‐based learning	Mind wandering (MW), EEG complexity, multiscale permutation entropy (MPE), detrended fluctuation analysis (DFA), band power (BP)	Modified probe‐caught method, EEG data recording, educational videos, comparison of MW and non‐MW states, various EEG complexity metrics, data augmentation, feature selection, mean AUC, eye‐movement artifacts, ethical approval, written informed consent, declaration of Helsinki, probe triggers, random intervals, manual triggers, facial expression monitoring, Neuroscan Grael system
Peloso et al. 2024; Cross‐cultural adaptation of the Mind‐Wandering Questionnaire (MWQ) for Brazilian Portuguese and evidence of its validity	2682 Brazilian adults (aged 18–69 years) from different regions	To adapt the Mind‐Wandering Questionnaire (MWQ) for the Brazilian context and present evidence of its validity	Mind wandering (MW), Mind‐Wandering Questionnaire (MWQ), confirmatory factor analysis, reliability indexes, McDonald's omega	Confirmatory factor analyses (CFA), multigroup confirmatory factor analyses (MGCFA), McDonald's omega for reliability, cross‐cultural adaptation, self‐report scale, Adult ADHD Self‐Report Scale (ASRS‐18), Maladaptive Daydreaming Scale (MDS‐16)
Qiu et al. 2024; The relationships between effortful control, mind wandering, and mobile phone addiction based on network analysis	1684 participants (aged 18–54 years) | To investigate the dimension‐level relationships between effortful control, mind wandering, and mobile phone addiction	To investigate the dimension‐level relationships between effortful control, mind wandering, and mobile phone addiction	Mobile phone addiction, effortful control, mind wandering, bridge expected influence (BEI), self‐report scales	Online survey through Wenjuanxing platform, convenience sampling, informed consent, anonymity emphasis, SPSS 26.0 for reaction time statistics, exclusion of repetitive/long/short responses, effective response rate, sociodemographic characteristics, Mobile Phone Addiction Tendency Scale, Effortful Control Questionnaire (Ellis et al.), Mind Wandering Questionnaire (Song et al.)
Franklin et al. 2017; Tracking distraction: the relationship between mind‐wandering, meta‐awareness, and ADHD symptomatology	105 participants (71 females, Mage = 23.1, SD = 7.4)	To assess the relationship between mind wandering and ADHD symptomatology, considering recent advances in mind wandering research	Mind wandering, ADHD symptoms, meta‐awareness, detrimental mind wandering, strategic mind wandering	Recruitment via posted flyers, laboratory measures, experience sampling during daily life, Sustained Attention to Response Task (SART), Reading and Mind‐Wandering Task, IPI questionnaire, ARCES scale, MAAS‐LO scale, Self‐Consciousness Scale,
Podda et al. 2022; Mind wandering in people with multiple sclerosis: a psychometric study	170 PwMS (people with multiple sclerosis)	To assess structural and construct validity and reliability of a brief Italian version of Mind Wandering (MW) Scale in PwMS	Mind wandering (MW), spontaneous MW (MW‐S), deliberate MW (MW‐D), structural validity, construct validity, reliability	Explorative factor analysis (EFA), construct validity correlation with mood and personality (Hospital Anxiety Depression Scale, Big Five Inventory Test)
Mckeown et al. 2020; The relationship between individual variation in macroscale functional gradients and distinct aspects of ongoing thought	254 healthy participants (169 females, mean age 20.7 ± 2.4 years)	To explore the relationship between whole‐brain functional connectivity patterns at rest and patterns of ongoing thought	Ongoing thought, functional connectivity, resting‐state fMRI, sensorimotor system, visual system	Resting‐state functional magnetic resonance imaging (rs‐fMRI), retrospective questionnaire assessing ongoing thoughts, nonlinear dimension reduction algorithm
Smallwood et al. 2013; The default modes of reading: modulation of posterior cingulate and medial prefrontal cortex connectivity associated with comprehension and task focus while reading	61 healthy native German speakers (mean age = 27.9 years; 37 females)	To explore whether the intrinsic functional connectivity of DMN hubs is predictive of individual differences in reading comprehension and task focus	Reading comprehension, task focus, default mode network (DMN), posterior cingulate cortex (PCC), anterior medial prefrontal cortex (aMPFC)	Resting‐state functional magnetic resonance imaging (rs‐fMRI), intrinsic functional connectivity, individual differences, sample of native German speakers, three excerpts from “A Short History of Everything” by Bill Bryson, retrospective assessment of ongoing thoughts, text comprehension assessment, MRI data acquisition and analysis, Max Planck Institute for Human Cognitive and Brain Sciences database
Martz et al. 2023; Disentangling racing thoughts from mind wandering in adult attention deficit hyperactivity disorder	84 adults with ADHD (aged 18–57, M = 32.48 years, 57.12% female)	To disentangle mind wandering (MW) from racing thoughts in adults with ADHD and investigate their associations with ADHD symptomatology	Mind wandering (MW), racing thoughts, ADHD symptomatology, emotional lability, functional impairment	Self‐reported questionnaires (Mind Wandering‐Deliberate, Mind Wandering‐Spontaneous, Racing and Crowded Thoughts Questionnaire, Daydreaming Frequency Scale), factorial analysis, multiple linear regressions, recruitment from outpatient psychiatry clinics of the University Hospital of Strasbourg, DSM‐5 criteria for ADHD and comorbidities diagnosis, no exclusion criteria, demographic data, comorbidities and treatment records, written informed consent, Declaration of Helsinki, data analysis
Shinagawa et al. 2021; Temporal changes in attentional resources consumed by mind‐wandering that precede awareness: an ERP study	33 healthy university students (21 women; mean age 20.93 years)	To elucidate whether changes in attentional resources used by mind wandering (MW) exist	Mind wandering (MW), attentional resources, P300 (P3), event‐related potentials (ERPs), self‐caught method	Simple reaction task with tone presentation, self‐reports of MW, electroencephalography (EEG) recording, state–space model analysis, P3 amplitude measurement, randomized interstimulus interval, auditory stimuli, stopping response upon MW realization, Keio University Research Ethics Committee approval, written informed consent, data analysis
Ibaceta et al. 2024; Mind‐wandering contents and characteristics: an exploratory study comparing between work and non‐work contexts	148 Chilean employees	To explore the nuances of mind wandering by examining its content and characteristics across work‐related and nonwork‐related settings	Mind wandering, work‐related content, nonwork‐related content, deliberateness, temporal orientation, Context and Content Regulation Hypothesis	Daily prompts for 5 working days, three times daily reporting, social media recruitment (LinkedIn, Twitter), snowball sampling technique, full‐time and part‐time employees, range of occupations, no direct compensation, raffle entry, 15‐item Mindful Attention Awareness Scale, diary entries on nature of thought
Brosowsky et al. 2023; Mind wandering, motivation, and task performance over time: evidence that motivation insulates people from the negative effects of mind wandering	166 participants	To examine whether participant motivation is associated with fluctuations of attentional engagement and performance over time	Mind wandering, motivation, attentional engagement, task performance, Metronome Response Task (MRT)	Participants completed “human intelligence task” on Amazon Mechanical Turk, Metronome Response Task (MRT), self‐reported motivation and mind wandering
Hong et al. 2023; Relationship of momentary volition to occupational experience and life perspective in undergraduate students	42 Korean university students	To investigate how momentary volition affects activity satisfaction and mind wandering while performing occupations, and examine its relationship with life satisfaction and life balance	Momentary volition, activity satisfaction, mind wandering, life satisfaction, life balance, Model of Human Occupation	Experience sampling method (ESM), Satisfaction With Life Scale (SWLS), Life Balance Inventory (LBI), participants received ESM survey eight times a day for 1 week, reports on current mood,
McVay et al. 2009; Tracking the train of thought from the laboratory into everyday life: an experience‐sampling study of mind wandering across controlled and ecological contexts	72 participants (undergraduates aged 18–35)	To investigate the relationship between mind wandering in laboratory tasks and everyday life, and its impact on task performance	Mind wandering, executive control, task performance, thought probes, daily‐life performance	Laboratory Sustained Attention to Response Task (SART), go/no‐go responses, thought probes, experience‐sampling method (ESM) with Palm Pilot PDAs, daily‐life activities, self‐reports of mind wandering, comparison of laboratory and everyday life mind wandering
Macaulay et al. 2022; Comparing the effect of mindful and other engagement interventions in nature on attention restoration, nature connection, and mood	215 participants	To compare different forms of engagement with nature and their psychological benefits	Mindful engagement, directed engagement, mind wandering, unguided control, attention restoration, nature connection, mood	Online experimental study, 20‐min outdoor experience, surveys on state‐mindfulness, connection with nature, and mood, Sustained Attention to Response Task (SART
Brishtel et al. 2020; Mind wandering in a multimodal reading setting: behavior analysis and automatic detection using eye‐tracking and an EDA sensor	21 graduate and undergraduate students (17 male; mean age 25.3 years)	To investigate the effects of text semantics and music on the frequency and type of mind wandering, and to propose a technique for automatic detection of mind wandering	Mind wandering, text semantics, music, eye‐tracking, electrodermal activity (EDA), automatic detection	Repeated‐measure design, independent variables (Text Type and Music Type), dependent variables (Interest, Difficulty, Tiredness, Perceived Mood, Attentional Focus, Type of Thoughts), self‐reports, behavioral data, physiological measurements, Random Forest classification model, recruitment from University of Kaiserslautern, course credits or gift card, scientific texts in German, audio stimuli from Taruffi et al.,
Marchetti et al. 2014; Self‐generated thoughts and depression: from daydreaming to depressive symptoms	117 native Dutch‐speaking students at Ghent University (mean age 21.51 ± 3.04, range: 20–46; 116 females, 11 males)	To investigate mechanisms linking daydreaming to depressive symptoms	Daydreaming, depressive symptoms, self‐focus, ruminative thought, indirect effect model	Questionnaires: Daydreaming Frequency Scale (DDFS), Self‐Reflection and Insight Scale (SRIS), Ruminative Responses Scale (RRS), Beck Depression Inventory second Edition (BDI‐II), Mindful Attention Awareness Scale (MAAS), recruitment of native Dutch‐speaking students, Ethical Committee approval, data analysis
Simpraga et al. 2021; Adults with autism spectrum disorder show atypical patterns of thoughts and feelings during rest	88 adults with autism spectrum disorder (ASD) and 90 controls	To investigate the character and content of thoughts and feelings experienced during mind wandering episodes in adults with ASD	Mind wandering, thoughts and feelings, autism spectrum disorder (ASD), Amsterdam Resting‐State Questionnaire (ARSQ)	Eyes‐closed rest condition to stimulate mind wandering, ARSQ to quantify subjective psychological states, recruitment of ASD cohort through Netherlands Autism Register, clinical diagnosis of ASD, inclusion criteria, control cohort (university students and project controls), exclusion criteria, AQ‐short questionnaire
Berry et al. 2014; Shared and distinct factors driving attention and temporal processing across modalities	64 participants (32 visual CTET: 16 female, mean age 18.63 years; 32 auditory CTET: 19 female, mean age 18.84 years)	To examine how modality, distraction, and time‐on‐task affect duration judgments and to identify factors driving performance across modalities	Temporal processing, modality effects, distraction, time‐on‐task, Continuous Temporal Expectancy Task (CTET), auditory and visual conditions	Visual and auditory versions of CTET, video distractor, performance measurement, behavioral and electrophysiological measures of mind wandering and attention lapses
Jiang et al. 2024; The cognitive neural mechanism of trait anxiety influences mind wandering: an ERP study	20 individuals with high trait anxiety (HTA) and 20 with low trait anxiety (LTA)	To examine the neural and cognitive time courses of how trait anxiety affects mind wandering	Trait anxiety, mind wandering, event‐related potentials (ERPs), Sustained Attention to Response Task (SART), late positive component (LPC), late slow waveform (LSW)	Recruitment of 600 volunteer university students, Trait Anxiety Inventory (T‐AI), selection of highest and lowest T‐AI scores, ERP experiment, comparison of behavioral performance and electroencephalographic waves, SART, response to frequent nontargets and infrequent targets, LPC and LSW measurement
Greve and Was 2022; Mind wandering probes as a source of mind wandering depends on attention control demands	138 undergraduates	To examine how the number of thought probes influences mind wandering during tasks that require a greater deal of thought control	Mind wandering, thought probes, attention control, operation span task (OSPAN), video lecture	Random assignment to four conditions (1, 3, 5, or 6 probes), OSPAN task with mathematical operations and letter recall, practice blocks, thought probes during experimental trials, Likert‐type scale for mind wandering
Rodriguez‐Larios and Alaerts 2020; EEG alpha‐theta dynamics during mind wandering in the context of breath focus meditation: an experience sampling approach with novice meditation practitioners	25 novice meditation practitioners (11 males; mean age 23.46 years, age range: 20–29)	To assess spectral changes in the theta‐alpha frequency range during mind wandering in the context of breath focus meditation	Mind wandering, theta oscillations (4–8 Hz), alpha oscillations (8–14 Hz), amplitude, frequency, harmonicity, phase synchrony	Probe‐caught experience sampling paradigm, electroencephalography (EEG) measurement, repeated probes to report focus on breath or mind wandering
Allen et al. 2013; The balanced mind: the variability of task‐unrelated thoughts predicts error monitoring	42 participants (27 females, mean age 34.8 years)	To examine the influence of subjective intensity, ruminative quality, and variability of mind wandering on response inhibition and monitoring	Task‐unrelated thoughts (TUTs), error monitoring, response inhibition, Error Awareness Task (EAT), mind wandering, BOLD activity, DMN, salience networks, executive networks	Recruitment from online participant pool system in Aarhus, Denmark, inclusion of mindfulness meditation practitioners, fMRI scans, informed consent, compensation, matched groups for age, gender, education, 6 runs of EAT, debriefing survey, psychophysical vision sensitivity test
Kane et al. 2021; Testing the construct validity of competing measurement approaches to probed mind‐wandering reports	1108 undergraduates (760 UNCG and 348 WCU students)	To explore the construct validity of probed mind wandering reports with a combined experimental and individual‐differences approach	Task‐unrelated thought (TUT), mind wandering, TUT‐content probes, TUT‐intentionality probes, TUT‐depth probes, performance‐evaluative thoughts	Recruitment from UNCG and WCU, comparison of four thought‐probe types across two cognitive tasks, demographics of participants, cognitive tasks (Antisaccade letters, Semantic Sustained Attention to Response Task), self‐report questionnaires (Dundee Stress State Questionnaire),
Krasich et al. 2020; Where the eyes wander: the relationship between mind wandering and fixation allocation to visually salient and semantically informative static scene content	51 volunteers from the University of Notre Dame	To assess how fixations are allocated to visually salient and semantically informative scene content prior to self‐reported mind wandering	Mind wandering, fixation allocation, visual salience, semantic informativeness, gaze control	Reanalysis of data from Krasich et al. (2018), static scene viewing task, probe‐caught self‐reported mind wandering, computational models of visual salience, semantic interest maps, meaning maps, recruitment from university psychology subject pool, course credit compensation, Amazon Mechanical Turk (MTurk), university IRB approval, data analysis
Bozhilova et al. 2022; Context regulation of mind wandering in ADHD	56 individuals (27 with ADHD and 29 controls)	To understand the association between MW frequency and clinical measures, context regulation of MW, and group differences in task performance	Mind wandering (MW) frequency, context regulation, sustained attention, working memory, task performance	Tasks manipulating demand on working memory and sustained attention, MW frequency recorded using probes, comparison of ADHD and control groups, matching on age, sex, and IQ, Sustained Attention task (SAT) with three levels of sustained attention load
Henríquez et al. 2015; Fluctuating minds: spontaneous psychophysical variability during mind‐wandering	33 healthy undergraduates (three males; mean age 23.71 years, range 18–30)	To investigate whether the transition from on‐task state to mind wandering is a gradual process or an abrupt event	Mind wandering, response times (RTs), behavioral variability, psychophysical performance	Continuous, online assessment of individual psychophysical performance, probe questions when RTs exceeded 2 standard deviations, comparison of RTs preceding mind wandering and on‐task reports
Groot et al. 2022; Catching wandering minds with tapping fingers: neural and behavioral insights into task‐unrelated cognition	27 healthy adult volunteers (15 male; mean age = 27.5 years, age range: 20–45)	To investigate the relationship between mind wandering and executive functions using a finger‐tapping task	Mind wandering, executive functions, finger‐tapping task, functional magnetic resonance imaging (fMRI), pupillometry, attention networks	Finger‐Tapping Random‐Sequence Generation Task, periodic experience sampling during fMRI and pupillometry, increases in finger‐tapping variability, entropy of random finger‐tapping sequences, neural correlates of behavioral performance, pupillary dynamics, self‐reported attentional state, recruitment from Amsterdam ultra‐high field adult lifespan database (AHEAD),
Shi et al. 2022; Relations between physical activity and hippocampal functional connectivity: modulating role of mind wandering | experimental | 99 healthy adults (mean age 22.78 years, SD 2.91; 49 female)	99 healthy adults (mean age 22.78 years, SD 2.91; 49 female)	To examine whether mind wandering modulated the relations between physical activity and resting‐state hippocampal functional connectivity	Physical activity, hippocampal functional connectivity, mind wandering, International Physical Activity Questionnaire (IPAQ), Mind Wandering Questionnaire (MWQ)	Neuroimaging data collection, IPAQ to measure physical activity level, MWQ to measure propensity to mind wandering, exclusion of participants with excessive head motion during fMRI or incomplete questionnaires
Yang et al. 2022; The steady state visual evoked potential (SSVEP) tracks “sticky” thinking, but not more general mind‐wandering	40 participants (25 female and 15 male)	To examine whether the SSVEP can track and allow for the prediction of the stickiness and task‐relatedness dimensions of spontaneous thought	Mind wandering, steady‐state visual evoked potential (SSVEP), stickiness of thoughts, task‐relatedness, sustained attention to response task (SART)	Modified SART to incorporate SSVEP elicited by 12.5‐Hz flicker, recruitment through social media, exclusion of participants with a history of epilepsy, Perseverative Thinking Questionnaire (PTQ), Rumination Response Scale (RRS), CES‐D for depression severity, screening and selection based on standardized scores, additional
				questionnaires before the experiment, embedding concerns in SART task, EEG recording
Cheung and Djekou 2024; Self‐compassion and grit mediated the relation between mindfulness and mind wandering based on cross‐sectional survey data	487 self‐identified meditators (241 female, mean age 38.98 years)	To test self‐compassion and grit as mediators for the relation between mindfulness and mind wandering	Mindfulness, self‐compassion, grit, mind wandering	Mind Excessively Wandering Scale (MEWS)
Whitehead et al. 2021; Mind wandering at encoding, but not at retrieval, disrupts one‐shot stimulus‐control learning	60 Amazon Mechanical Turk workers (31 women, mean age 37.03 years, SD 10.74)	To examine the role of task‐focused attention in the encoding and implementation of stimulus‐control bindings in episodic event‐files	Mind wandering, stimulus‐control bindings, episodic memory, task switching, pupillometry, attention levels	self‐reports of mind wandering during encoding and implementation,
Alperin et al. 2021; More than off‐task: increased freely‐moving thought in ADHD	79 individuals (40 controls, 39 ADHD)	To examine the differences in the amount and type of off‐task thought in individuals with ADHD compared to those without ADHD	Off‐task thought, freely moving thought, constrained thought, self‐report, cognitive performance, EEG measures	Self‐report measures, cognitive performance tasks, EEG recording, Sustained Attention to Response Task (SART), thought probes, thought probe confidence, reaction time variability, attention task errors
Smith et al. 2023; Examining the relation between oral contraceptive use and attentional engagement in everyday life	| Study 1: 1801 participants (471 using OCs, 1330 naturally cycling), Study 2: 1175 participants (246 using OCs, 929 naturally cycling)	To examine the relation between oral contraceptive use and self‐reported everyday attention	Oral contraceptives (OCs), mind wandering, attention‐related errors, attention lapses	Spontaneous (MWS) and deliberate (MWD) mind wandering scales, Mindful Attention Awareness Scale‐Lapses Only (MAASLO), Attention‐Related Cognitive Errors Scales (ARCES)
Soffer‐Dudek 2018; Dissociative absorption, mind‐wandering, and attention‐deficit symptoms: associations with obsessive‐compulsive symptoms	303 Israeli undergraduate students (74.3% females; mean age 23.5, SD 1.4)	To validate dissociative absorption as unique from ADHD symptoms and mind wandering, and show its incremental predictive value in predicting OC symptoms	Dissociative absorption, mind wandering, attention‐deficit/hyperactivity disorder (ADHD), obsessive‐compulsive (OC) symptoms	The Mind‐Wandering questionnaire (MWQ; Mrazek et al. 2013a), a self‐report measure for the assessment of task‐unrelated thought
Sayette et al. 2010; Out for a smoke: the impact of cigarette craving on zoning out during reading	44 native‐English‐speaking smokers (ages 18–55)	To examine the effects of cigarette craving on the occurrence and awareness of mental lapses during reading	Cigarette craving, mind wandering, mindless‐reading task, self‐caught and probe‐caught mind wandering episodes, metacognitive capacity	probe‐caught zoning out (response to prompts)
Kim Lux et al. 2023; Getting personal: brain decoding of spontaneous thought using personal narratives	49 healthy right‐handed Koreans (21 female, mean age 22.8 years)	To decode the content dimensions of spontaneous thought—self‐relevance and valence—directly from fMRI signals	Spontaneous thought, self‐relevance, emotional valence, functional Magnetic Resonance Imaging (fMRI), default mode and ventral attention networks	fMRI experiment with story‐reading and thought‐sampling tasks
Hidaka et al. 2023; No relationships between frequencies of mind‐wandering and perceptual rivalry	Study 1: 181 participants (72 females, mean age 42.96 years); Study 2: 100 participants (70 females, mean age 25.12 years)	To investigate possible relationships between mind wandering and perceptual rivalry	Mind wandering, perceptual rivalry, Necker‐cube (NC), structure‐from‐motion (SfM), Sustained Attention to Response Task (SART)	intentional mind wandering and perceptual rivalry induction, Japanese versions of MWQ, MW‐D/MW‐S
Fanning et al. 2016; Physical activity, mind wandering, affect, and sleep: an ecological momentary assessment	33 college‐aged adults	To examine associations between hourly mind wandering and moderate‐to‐vigorous physical activity (MVPA), and the impact of affect and daily sleep on these relations	Mind wandering, MVPA, affect, sleep quality and duration	mobile phone‐delivered prompts assessing mind wandering and affect
Smith et al. 2022; Fixation, flexibility, and creativity: the dynamics of mind wandering	225 participants (95 women, mean age 38.13 years)	To test the predictions of the Dynamic Framework of mind wandering, which characterizes mind wandering as “freely moving” thoughts	Freely moving thought, ADHD, depression, anxiety, OCD, divergent thinking	thought probes, Short‐Form of the Adult Self‐Report ADHD Scale, Dimensional Obsessive‐Compulsive Scale, Depression, Anxiety, and Stress Scale
Chiorri et al. 2023; The role of mindfulness, mind wandering, attentional control, and maladaptive personality traits in problematic gaming behavior	506 participants from the Italian general population	To investigate the pattern of association between mindfulness, mind wandering, attentional control, maladaptive personality traits, and problematic gaming	mindfulness, mind wandering (spontaneous and deliberate), attentional control, maladaptive personality traits	Five Facet Mindfulness Questionnaire‐15 (FFMQ‐15), Mind Wandering‐Spontaneous and Deliberate scales (MW‐S and MW‐D), Attention Control‐Distraction (AC‐D) and Attentional Control‐Shifting (AC‐S) scales, Personality Inventory for DSM‐5‐Brief Form (PID‐5‐BF),
Smith et al. 2023; The relation between trait flow and engagement, understanding, and grades in undergraduate lectures	139 undergraduate psychology students (final sample)	To explore the relation between trait‐level flow and in‐class reports of engagement, understanding, and academic performance	Trait flow, engagement, understanding, course grades, mind wandering, grit	experience sampling probes during lectures via laptop application, measurement of trait flow, mind wandering
Zhang et al. 2019; Distinct individual differences in default mode network connectivity relate to off‐task thought and text memory during reading	69 undergraduate or postgraduate students (mean age 19.87 years)	To understand the neural processes underpinning off‐task thought and its impact on text memory	Off‐task thought, text‐based memory, default mode network connectivity, fMRI	behavioral assessments (reading and off‐task thought), naturalistic reading experience with a printed booklet, participants circling words when attention wandered, answering open‐ended questions about the text, New‐York Cognition Questionnaire (NYC‐Q), structural and functional neuroimaging with 3T GE HDx Excite MRI
Deng et al. 2022; The effect of mind wandering on cognitive flexibility is mediated by boredom	Study 1: 449 participants (338 males, 111 females; mean age 19.61 years), Study 2: 182 participants (76 males, 106 females; mean age 20.60 years), Study 3: 190 participants (71 males, 119 females; mean age 21.68 years)	To investigate the hypothesis that boredom can mediate the effect of mind wandering on cognitive flexibility at trait‐level	Mind wandering (spontaneous and deliberate), boredom, cognitive flexibility	Mind‐Wandering Questionnaire (MWQ), Mind Wandering scale: Deliberate and Spontaneous (MW‐D and MW‐S)
El Haj et al.; The subjective experience of mind wandering in Alzheimer's disease | survey study	32 AD patients (19 women, 13 men; mean age 71.32 years) and 35 control older adults (22 women, 13 men; mean age 72.16 years)	To evaluate the subjective experience of mind wandering in Alzheimer's disease (AD)	Mind wandering, occurrence, intentionality, emotionality, visual imagery, specificity, self‐relatedness, temporal orientation	questionnaire inspired by general definitions of mind wandering, rating of mind wandering attributes (occurrence, intentionality, emotionality, visual imagery, specificity, self‐relatedness, temporal orientation),
Andrews‐Hanna et al. 2010; Evidence for the default network's role in spontaneous cognition	199 right‐handed young adults (97 male; mean age 22.2 years)	To examine the default network's role in spontaneous cognition by manipulating factors promoting spontaneous cognition separately from changes in external attention	Default network activity, spontaneous cognition, medial temporal lobe, distributed cortical regions, self‐report questionnaires, fMRI	functional MRI study, self‐report questionnaires during extended fixation epochs
Wießner et al. 2021; Low‐dose LSD and the stream of thought: increased discontinuity of mind, deep thoughts and abstract flow	24 healthy participants	To elucidate the effects of LSD on the stream of thought	Mind wandering, Amsterdam Resting State Questionnaire (ARSQ), free association, Forward Flow Task (FFT)	mind wandering measured by ARSQ
Cárdenas‐Egúsquiza and Berntsen 2023; Individual differences in autobiographical memory predict the tendency to engage in spontaneous thoughts	Study 1: 292 participants (excluding 31 for failing attention checks); Study 2: 257 participants (excluding four for failing attention checks or providing incoherent answers)	To examine individual differences in autobiographical memory in relation to various forms of spontaneous thought and fantasy proneness	Autobiographical memory, spontaneous thought, fantasy proneness	Study 1: Recruitment via Amazon Mechanical Turk, self‐report questionnaires (ART, MWQ, DDFS, MW‐D, MW‐S, IAMI, PANAS), hierarchical regressions; Study 2: Recruitment via Prolific, self‐report questionnaires (same as Study 1 plus CEQ)
Hou et al. 2023; Mindfulness profiles among Chinese university students: exploring differences in phenomenon, cognition, and performance of mind wandering	1557 Chinese university students (67% women; mean age 21.27 years)	To investigate the latent profiles of mindfulness and explore their links to mind wandering outcomes	Mindfulness, mind wandering, cognitive errors, sustained attention response task (SART)	SART, questionnaire (FFMQ, ARCES, DMW, SMW, MWQ),
Taruffi 2021; Mind‐wandering during personal music listening in everyday life: music‐evoked emotions predict thought valence	26 participants (18 female; mean age 30.46 years)	To capture mind wandering during personal music listening in everyday life and test the capacity of music to facilitate beneficial styles of mind wandering	Mind wandering, mood, music‐evoked emotions, thought valence	experience sampling methodology using MuPsych app
D'Anselmo et al. 2020; Creativity in narcolepsy type 1: the role of dissociated REM sleep manifestations	66 NT1 patients (31 females, mean age 38.62 years)	To explore the role of narcolepsy symptoms in predicting creativity	Narcolepsy symptoms, creativity, mind wandering, sleep paralysis, hypnagogic hallucinations	questionnaire on mind wandering (MW‐D and MW‐S)
Welhaf and Kane 2023; A nomothetic span approach to the construct validation of sustained attention consistency: re‑analyzing two latent‑variable studies of performance variability and mind‑wandering self‑reports	58	To examine the construct validity of attention consistency by reanalyzing two latent‐variable studies of performance variability and mind wandering self‐reports	Sustained attention consistency, reaction time (RT) variability, task‐unrelated thought (TUT), working memory capacity, attention control, processing speed, state motivation, alertness, cognitive failures, positive schizotypy	Psychomotor Vigilance Task (PVT), Semantic SART, Choice RT (CRT), Continuous Tracking, Working Memory Capacity (WMC) tasks, Attention Control tasks, Stroop, Cognitive Failures Questionnaire—memory and attention lapses (CFQ‐MAL)
Chaieb and Fell 2024; Insights into the time course of mind wandering during task execution	74 participants across two studies (Study 1: 40 participants, mean age 26.5 years; Study 2: 34 participants, mean age 25.7 years)	To examine the time course of mind wandering (MW) during a sustained‐attention‐to‐response task (SART) and its interaction with auditory beat stimulation	Mind wandering, meta‐awareness, temporal orientation, auditory beat stimulation, monaural beat	self‐rating MW questionnaire (Mrazek et al. 2013a), experience sampling probes
Peper et al. 2019; Which quiets the mind more quickly and increases HRV: toning or mindfulness?	Observation 1: 91 undergraduate students (average age 22.4 years); Observation 2: 11 undergraduate students (average age 21.4 years)	To compare mindfulness practice (MP) and toning practice (TP) in reducing mind wandering and intrusive thoughts, and increasing heart rate variability (HRV)	Mind wandering, intrusive thoughts, body vibrations, peacefulness, relaxation, stress, warmth, anxiety, depression, respiration rate, heart rate variability (HRV)	Tasks and Materials:** Mindfulness practice (MP), Toning practice (TP), subjective assessment form, eight‐channel polygraph, respiration monitoring, heart rate monitoring
Ceh et al. 2021; Neurophysiological indicators of internal attention: an fMRI‐eye‐tracking coregistration study	30 participants (20 female; average age 22.7 years)	To investigate brain mechanisms and eye behavior related to internally vs. externally directed cognition	Internally directed cognition, lingual gyrus, inferior parietal lobe, visual networks, pupil diameter, blink duration, fixation disparity, microsaccades, fMRI‐eye‐tracking covariation	fMRI, eye tracking, conditional stimulus masking, questionnaire for MRI safety, written informed consent, data exclusion criteria
Irish et al. 2018; Age‐related changes in the temporal focus and self‐referential content of spontaneous cognition during periods of low cognitive demand	30 younger adults (mean age 31.3 years) and 33 healthy older adults	To explore age‐related changes in off‐task, self‐generated thought (mind wandering) under low cognitive demand	Mind wandering, temporal focus, self‐referential/social content, Shape Expectations task, daydreaming propensity	coding of mind wandering
Heinilä et al. 2023; Penalized canonical correlation analysis reveals a relationship between temperament traits and brain oscillations during mind wandering	29 participants (aged 21–48 years)	To investigate the relationship between brain oscillatory activity and temperament traits during mind wandering	Brain oscillatory activity, temperament traits, behavioral inhibition, anxiety, alpha and beta power, brain oscillation patterns	Focused attention (FA), self‐centered future planning (FP), rumination on anxious thoughts (AT), magnetoencephalography (MEG), spatial contrast maps, permutation cluster tests, penalized canonical correlation analysis (CCA), trait questionnaires (BIS/BAS, BDI, BAI),
				visual imagery stimuli (anxiety‐inducing and neutral images)
Carciofo 2019; Morningness‐eveningness and tertiary academic achievement: an exploration of potential mediators, including sleep factors, mind wandering, and metacognitive beliefs	153 s‐year undergraduate Business School students	To explore the relationship between morningness–eveningness and academic achievement, and investigate potential mediators	Morningness–eveningness, academic achievement, sleep quality, mind wandering, conscientiousness, affect, metacognitive beliefs	Morningness–Eveningness Questionnaire (rMEQ), Mind Wandering scale (Imaginal Processes Inventory), Pittsburgh Sleep Quality Index (PSQI), Positive and Negative Affect Schedule (PANAS), Big Five Inventory (Conscientiousness scale)
Figueiredo and Mattos 2021; Disentangling the phenomenology of mind‐wandering	53 participants (54.7% male; age range 18–36 years)	To explore the phenomenology of mind wandering (MW) and its relation to ADHD, impulsivity, anxiety, and depressive symptoms	Mind wandering, ADHD, impulsivity, anxiety, depressive symptoms	Mind Excessively Wandering Scale (MEWS), psychiatric, neuropsychological, and language assessments, ADHD symptoms, anxiety and depressive symptoms
Gruberger et al. 2013; The wandering mood: psychological and neural determinants of rest‐related negative affect	29 participants (age: 33 ± 11; 12 females)	To investigate the psychological and neural aspects of rest‐related negative affect (RRNA) during resting‐state and its relation to mind wandering (MW) and vigilance	Rest‐related negative affect (RRNA), mind wandering (MW), vigilance levels, functional connectivity (FC), default mode network (DMN), executive network (EXE), salience network (SAL)	fMRI‐EEG simultaneous scans, positive and negative affect scale (PANAS), visual analog scale for MW, baseline rest scan, intermediate task, postscan questionnaires, data analysis of functional connectivity, EEG‐based vigilance levels
Kawashima et al. 2022; Pavlovian‐based neurofeedback enhances meta‐awareness of mind‐wandering	36 participants (23 males; mean age 29.25 years)	To investigate the effectiveness of Pavlovian‐based neurofeedback in enhancing meta‐awareness of mind wandering (MW)	Mind wandering, meta‐awareness, Pavlovian conditioning, electroencephalogram (EEG), behavioral measures, neuroscientific measures	Sustained Attention Response Task (SART)‐A, SART‐B, breath attention task, Quick‐30 EEG headset, electrooculography (EOG) electrode, machine‐learning estimation method, real‐time feedback on neural information
Li et al. 2022; Neural representations of self‐generated thought during think‐aloud fMRI	86 participants (45 females; mean age = 22.1 ± 2.7 years)	To investigate the role of self‐generated thoughts in resting‐state fMRI and their neural representation	Self‐generated thoughts, resting‐state fMRI, think‐aloud method, brain activation patterns, representational similarity analysis (RSA)	Think‐aloud method, MRI data acquisition, verbal report data preprocessing, fMRI data preprocessing, voxel‐wise whole‐brain searchlight level analysis
Zhang et al. 2020; Wandering eyes: eye movements during mind‐wandering in video lectures	Study 1: 72 participants (mean age = 18.86 years); Study 2: 71 participants (mean age = 18.85 years)	To examine eye movement patterns of mind wandering during video lectures	Mind wandering, eye movements, fixations, dispersion, engagement, comprehension	Video lectures, self‐caught reports, thought probes, eye tracking
Ibaceta and Madrid 2021; Personality and mind‐wandering self‐perception: the role of meta‐awareness	273 college students (44% female; average age 19.01 years)	To explore the relationship between personality traits and mind wandering self‐perception, mediated by meta‐awareness	Mind wandering, personality traits, neuroticism, openness to experience, meta‐awareness	Three‐wave survey, Spanish version of Benet‐Martínez and John scales (five‐factor model of personality), Mind‐Wandering Questionnaire, cognitive ability assessment, meta‐awareness assessment, data analysis
Liu et al. 2021; The neural markers of self‐caught and probe‐caught mind wandering: an ERP study	40	To explore the neural patterns of different types of mind wandering (MW) using event‐related potentials (ERPs)	Self‐caught mind wandering, probe‐caught mind wandering, reaction times (RTs), ERP components (N1, P2, N300, P300)	Participants performed a modified sustained attention to response task (mSART); MW was captured via self‐reports (self‐caught MW) and probes (probe‐caught MW); ERP data
Zhang et al. 2020; Refixation patterns of mind‐wandering during real‐world scene perception	57 participants (mean age = 18.84 years)	explore how mind wandering (MW) affects eye movement patterns during real‐world scene perception	Mind wandering, eye movements, refixations, fixation sequences, scene memory	Real‐world scene encoding task, thought probes, Recurrence Quantification Analysis (RQA), eye tracking using Eyelink 1000 tracker, OpenSesame software, PyGaze package
Warden et al. 2018; Absence of age effects on spontaneous past and future thinking in daily life	Study 1: 40 participants (21 young adults, mean age 21.71 years; 19 older adults, mean age 72.32 years); Study 2: 47 participants (24 young adults, mean age 25.00 years; 23 older adults, mean age 74.35 years)	To investigate the effects of age on the frequency and nature of spontaneous future thoughts in everyday life	Spontaneous future thoughts, involuntary autobiographical memories, prospective memory tasks	Study 1: 2‐week diary study, spontaneous thoughts of prospective memory tasks and involuntary autobiographical memories; Study 2: 1‐day experience sampling method, WatchMinder3 wristwatch, 30 random signals, thought recording, Hospital Anxiety and Depression Scale, Martin and Park Environmental Demands Questionnaire
Nakatani et al. 2019; Long‐term dynamics of mind wandering: ultradian rhythms in thought generation	32 participants (69% female; mean age 19 years)	To study the fluctuations in thought generation and cognitive control strength during the day centered around episodes of mind wandering	Thought generation, cognitive control strength, mind wandering, ultradian rhythms	Participants received a short message on their smartphone with a link to a probe; the probe consisted of a visual task and a questionnaire; the task involved rating of scene closeness to estimate strength of deliberate constraints; the questionnaire asked participants to report the number and type of their thoughts
Thomson et al. 2014; The more your mind wanders, the smaller your attentional blink: an individual differences study	Study 1: 121 undergraduates (48 male, 73 female); Study 2: 102 undergraduates (12 male, 90 female)	To examine whether self‐reported instances of mind wandering predict the magnitude of the attentional blink (AB) in a rapid serial visual presentation (RSVP) task	Mind wandering, attentional blink (AB), sustained attention, Spontaneous and Deliberate Mind Wandering Questionnaire	Study 1: Sustained Attention to Response Task (SART), Rapid Serial Visual Presentation (RSVP) Task, Python programming, PsychoPy software, Mac Mini computer, Phillips 244E LCD monitor; Study 2: RSVP task with Lags of 2, 4, 6, and 8, Spontaneous and Deliberate Mind Wandering Questionnaires (MW:S and MW:D)
Price et al. 2023; Investigating the protective effects of mindfulness‐based attention training on mind wandering in applied settings	Five studies with a total of 304 participants from various organizational cohorts	To examine the effects of Mindfulness‐Based Attention Training (MBAT) on mind wandering in applied settings	Mind wandering, meta‐awareness, sustained attention, response time variability, standardized mean change (SMC)	Five longitudinal studies conducted by the research group; MBAT program consists of four 2‐h sessions over 4 weeks; themes: concentration, body awareness, receptivity, connection; mindfulness exercises: focused attention, body scan, open monitoring, connection practices; participants completed self‐reported measures and performance‐based metrics (SART) at baseline (T1) and 4 weeks later (T2); data analysis of standardized mean change (SMC)
Wereszczyński et al. 2023; Investigating the relationship between periodontitis and specific memory processes in the search for cognitive markers of Alzheimer's disease risk	60 community‐dwelling dementia‐free older adults (mean age = 72.52 years; 86% women)	To investigate the relationship between mind wandering and periodontitis as a risk factor for Alzheimer's disease, and to explore the association between periodontitis and memory	Mind wandering, periodontitis, Alzheimer's disease risk, episodic memory, spontaneous retrieval deficit (SRD) hypothesis	‐ Neuropsychological tests‐ Computer‐based Man‐made/Natural Task‐ Periodontal health assessment‐ Oral examination‐ Qualified dentist‐ Mini‐Mental State Examination (MMSE)‐ California Verbal Learning Test (CVLT)‐ Mind wandering evaluation‐ Thought probes
Faber et al. 2018; Driven to distraction: a lack of change gives rise to mind wandering	108 college students (66% female; average age = 20.1 years)	To examine the relationship between event structure and attention, specifically how narrative shifts predict attentional lapses	Mind wandering, event structure, narrative shifts, attentional lapses	Participants watched the film “The Red Balloon” and self‐reported instances of mind wandering; prior knowledge or control condition
Smallwood et al. 2011; Self‐reflection and the temporal focus of the wandering mind	Study 1: 45 undergraduate students (mean age = 20.3); Study 2: 70 undergraduate students (mean age = 20.5)	To explore the extent to which self‐reflection impacts retrospection and prospection during mind wandering	Mind wandering, self‐reflection, retrospection, prospection, self‐reference effect (SRE)	Study 1: Personality test, social network survey, political survey, trait adjectives, Working Memory (WM) task, Choice Reaction Time (CRT) task, experience sampling probes; Study 2: Trait‐ascription task, CRT task, experience sampling probes, surprise recognition‐memory test
Raij and Riekki 2017; Dorsomedial prefontal cortex supports spontaneous thinking per se	51 healthy participants (aged 21–41 years; mean age 31 years; 15 women)	To investigate brain activation linked to the experience of spontaneous thinking during mind wandering	Spontaneous thinking, mind wandering, brain activation, default mode network, cortical midline structures	experience sampling during 3T functional magnetic resonance imaging (fMRI); 32 subjects underwent two 15‐min fMRI sessions, 19 subjects completed a single session
Xu et al. 2017; Mindfulness and mind wandering: the protective effects of brief meditation in anxious individuals	82 undergraduate students (55 females; mean age = 20.0 years)	To examine the impact of mindfulness on mind wandering in highly anxious individuals	Mind wandering, mindfulness, meditation, sustained attention	Participants completed a sustained‐attention task with thought probes; randomly assigned to meditation or control condition; neuropsychological tests included STICSA, MAAS, and PANAS; mindfulness intervention involved listening to “Mindfulness of body and breath”; control intervention involved listening to “The Hobbit”; MRT was used to measure response time and thought probes assessed mind wandering
Bernhardt et al. 2014; Medial prefrontal and anterior cingulate cortical thickness predicts shared individual differences in self‐generated thought and temporal discounting	37 healthy volunteers (18 females; mean age = 27.0 years)	To investigate the structural brain basis of self‐generated task‐unrelated thought (TUT) and its relationship with temporal discounting (TD)	Self‐generated thought, task‐unrelated thought (TUT), temporal discounting (TD), cortical thickness, medial prefrontal cortex (mPFC), anterior/midcingulate cortex	Participants performed a choice reaction time (CRT) task and a 1‐back working‐memory (WM) task; TUT was recorded using thought probe sampling; TD task measured participants' preference for future rewards; MRI data were acquired using a 3T Siemens Verio scanner and processed using FreeSurfer for cortical thickness analysis
Marcusson‐Clavertz et al. 2016; Daydreaming style moderates the relation between working memory and mind wandering: integrating two hypotheses	111 students from Lund University (41 males, 70 females; mean age = 24.75 years)	To examine the interaction between working memory, daydreaming style, and mind wandering	Mind wandering, working memory, daydreaming style, control‐failure hypothesis, global availability hypothesis	Participants reported mind wandering over 4 days using experience sampling; completed the Sustained Attention to Response Task (SART), Symmetry Span (SSPAN) task, and Stroop task; daydreaming tendencies measured by the Short Imaginal Processes Inventory (SIPI); personal digital assistants (PDAs) used for experience sampling
Amos‐Oluwole et al. 2019; Compliant activity accelerates all thought probe responses and inhibits deliberate mind wandering	28 healthy subjects (19 females, nine males; mean age = 22.7 years)	To investigate the impact of compliant activity on mind wandering and response times	Mind wandering, compliant activity, thought probes, reaction times	Participants interacted with pairs of stimuli (low‐interactivity and high‐interactivity versions); mind wandering assessed by thought probes and visual analogue scales (VAS); reaction times measured using Superlab with an RB530 response pad; primary variables: response times, spontaneous and deliberate mind wandering, challenge, boredom
Pepin et al. 2021; Impact of mind‐wandering on visual information processing while driving: an electrophysiological study	60 healthy volunteers (age: 26.88 ± 4.08; 24 males)	To investigate how the level of attention devoted to driving impacts visual information processing and reaction time	Mind wandering, visual information processing, driving, Event‐Related Potentials (ERP), reaction time	Participants used a driving simulator equipped with a 24‐in. screen, speakers, a steering wheel, and pedals; stimulus presentation and response collection controlled with LEPSIS‐IFSTTAR simulator software (ArchiSim2); electrophysiological data recorded using Biosemi ActiveTwo system; EEG and EOG signals acquired; attention assessed using a continuous scale from 0 to 100 during twelve 3‐min driving
				sessions; comparison of sessions with highest and lowest reported levels of attention
Stawarczyk et al. 2014; Relationships between mind‐wandering and attentional control abilities in young adults and adolescents	164 French‐speaking participants (87 young adults, mean age 22.71 years; 77 adolescents, mean age 14.88 years)	To examine the relations between attentional control abilities and mind wandering in adolescents and young adults	Mind wandering, attentional control, external distractions, Sustained Attention to Response Task (SART)	Participants completed a listening span task (working memory capacity), AX‐Continuous Performance Test (proactive and reactive control), SART with thought‐probes (mind wandering and external distractions), general fluid intelligence assessment; questionnaires: CES‐D (depressive symptoms), BAI (anxiety), DDFS (daydreaming frequency)
Bozhilova et al. 2022; Event‐related brain dynamics during mind wandering in attention‐deficit/hyperactivity disorder: an experience‐sampling approach	23 participants with ADHD and 25 controls (adults)	To investigate brain dynamics during mind wandering in adults with ADHD using EEG	Mind wandering, ADHD, brain dynamics, EEG, experience‐sampling	Participants performed Mind Wandering Task and Sustained Attention Task (SAT); MW captured using experience‐sampling with thought probes (15 per session, 30 in total) at 1‐min intervals; EEG recording and preprocessing; recruitment centers: South London and Maudsley NHS Trust, Barnet, Enfield and Haringey Mental Health Trust adult ADHD clinics, online platforms, UKAAN
Brosowsky et al. 2022; On the relation between mind wandering, PTSD symptomology, and self‐control	5387 undergraduate psychology students (mean age 21)	To investigate the relationships between mind wandering, PTSD symptomology, and self‐control	Spontaneous mind wandering, deliberate mind wandering, PTSD symptomology, self‐control	Participants completed questionnaires: spontaneous and deliberate mind wandering scales, brief self‐control scale (BSCS), PTSD Checklist for DSM‐5 (PCL‐5); data analysis using linear regression models; power analysis conducted with “WebPower” R package
Pelagatti et al. 2019; A closer look at the timecourse of mind wandering: pupillary responses and behaviour	24 undergraduate students from the University of Florence (age range 19–32 years; mean age 21.50 years; 14 females)	To investigate pupillary changes associated with the onset and duration of self‐reported mind wandering (MW) episodes	Mind wandering, pupillary responses, vigilance task, self‐caught method	Participants performed a computer‐based vigilance task with cue‐words; MW episodes were self‐reported using the self‐caught method; eye position and pupil diameter were monitored with a CRS LiveTrack system; participants completed a thought questionnaire about their mental contents, temporal focus, specificity, emotional valence, concentration, and boredom
Robison 2019; Working memory capacity and mind‐wandering during low‐demand cognitive tasks	124 participants from the University of Oregon undergraduate pool	To investigate the relationship between working memory capacity (WMC) and mind wandering during low‐demand cognitive tasks	Working memory capacity, mind wandering, low‐demand tasks, context‐regulation hypothesis	| Participants completed measures of WMC (Operation span, Symmetry span, Reading span), choice reaction time task, and digit reaction time task; thought probes used to assess mind wandering
Durantin et al. 2015; Characterization of mind wandering using fNIRS	23 male students from ISAE school of engineering (mean age: 22.6; age range 21–24 years)	To test the ability of functional near‐infrared spectroscopy (fNIRS) to detect mind wandering (MW) episodes	Mind wandering, fNIRS, Sustained Attention to Response Task (SART), default mode network (DMN)	Computerized SART task, Sixteen‐channel fNIRS, Frontal cortices, Medial prefrontal cortex (mPFC), Mind wandering (MW), SART errors
Brennan et al. 2020; Intrasubject functional connectivity related to self‐generated thoughts	22 healthy individuals	To investigate the relationship between self‐generated thoughts (mind wandering) and functional connectivity (FC) during rsfMRI	Self‐generated thoughts, functional connectivity (FC), mind wandering	Multifactor analysis (MFA) of self‐generated thoughts during high‐field (7T) rsfMRI; network‐based statistic (NBS) method for whole‐brain connectivity analysis
Kane et al. 2021; Testing the construct validity of competing measurement approaches to probed mind‐wandering reports	1108 undergraduate students from UNCG and WCU	To explore the construct validity of probed mind wandering reports using different thought‐probe types	Task‐unrelated thought (TUT) reports, retrospective mind wandering ratings, executive‐control performance, questionnaire assessments	Comparison of four different thought‐probe types across two cognitive tasks; primary analyses compared probes asking about different dimensions of experience
Rodriguez‐Boerwinkle et al. 2024; Variation in divergent thinking, executive‐control abilities, and mind‐wandering measured in and out of the laboratory	541 UNCG undergraduates (first session), 492 (second session), 472 (third session)	To assess links between divergent thinking, executive control abilities, and mind wandering in lab and daily‐life contexts	Divergent thinking, executive control, working memory capacity (WMC), attention control, mind wandering (TUT rates)	Subjects completed six WMC measures (operation, reading, symmetry, rotation spans); TUT rates calculated from thought probes in five tasks; daily‐life mind wandering measured through a 7‐day experience sampling protocol
Preiss et al. 2016; Examining the influence of mind wandering and metacognition on creativity in university and vocational students	233 Chilean students (116 university, 117 vocational)	To examine the relationship between mind wandering, metacognition, and creativity	Mind wandering, metacognition, creativity, divergent thinking, creative problem solving, fluid intelligence	Participants took tests of divergent thinking, creative problem solving, fluid intelligence, and answered self‐report scales on mind wandering
Albert et al. 2022; Negative mood mind wandering and unsafe driving in young male drivers	40 healthy male drivers aged 20–24	To test whether negative mood increases MW and unsafe driving, and to examine the moderating role of trait rumination and inhibitory control	Mind wandering (MW), unsafe driving, negative mood, emotional arousal, trait rumination, inhibitory control	; driving simulator task; driving speed, headway distance, steering behavior, overtaking, heart rate, and thought probes
Xie et al. 2021; Spontaneous and deliberate modes of creativity: multitask Eigen‐connectivity analysis captures latent cognitive modes during creative thinking	32 participants (mean age 30.4 years; 13 females, four left‐handed)	To capture the latent modes of creative thinking using a data‐driven approach	Creative thinking, deliberate cognition, spontaneous thought, Eigen‐connectivity (EC), functional connectivity (FC)	Continuous multitask paradigm (CMP) with eight task blocks ranging from undirected mind wandering to goal‐directed working memory task; fMRI session
Smallwood et al. 2003; Task unrelated thought whilst encoding information	28 healthy participants from the Department of Psychology	To compare distributed and encapsulated views of cognition, and to examine the effects of TUT on information retrieval	Task unrelated thought (TUT), word‐fragment completion, word recognition	; thought probes used to classify thoughts; memory retrieval measured through word‐fragment completion and word recognition; experiments examined the impact of TUT on retrieval accuracy
Kandeğer et al. 2023; Mentation processes such as excessive mind wandering, rumination, and mindfulness mediate the relationship between ADHD symptoms and anxiety and depression in adults with ADHD	159 medication‐free adults with ADHD (ages 18–39)	To investigate whether mentation processes (excessive mind wandering, rumination, mindfulness) mediate the relationship between ADHD symptoms and anxiety, and depression	ADHD symptoms, mind wandering, rumination, mindfulness, anxiety, depression	sociodemographic form, Adult ADHD Severity Rating Scale, Hospital Anxiety Depression Scale, Mind Excessively Wandering Scale, Ruminative Response Scale, Freiburg Mindfulness Inventory
Krimsky 2018; The allocation of attentional resources: exploring fluctuations in mind wandering with variation in performance and affective variables	69 undergraduate students from the University of Miami (43 females; mean age 18.99)	To investigate mechanisms that increase mind wandering (MW) in relation to task requirements and individual differences tied to negative mood and depressive symptoms	Mind wandering (MW), task performance, negative mood, depression	Participants completed self‐report measures of depression and negative mood, and the sustained attention to response task (SART) with intermittent mind wandering probes
Franklin et al. 2013; The silver lining of a mind in the clouds: interesting musings are associated with positive mood while mind‐wandering	105 participants (71 female; mean age 23.1)	To investigate the relationship between mind wandering content and mood	Mind wandering, mood, experience sampling	Participants used PDAs to respond to experience sampling probes about mind wandering content and mood approximately eight times per day; rated off‐task thoughts and mood on a five‐point scale
May et al. 2011; An attentional control task reduces intrusive thoughts about smoking	27 volunteers (aged 20–61, mean age 30; 11 males)	To test the effect of body scanning instructions on smoking‐related thoughts and cravings	Smoking‐related thoughts, cravings, body scanning, attentional control	mind wandering and body scanning blocks, thoughts probed ten times per block; smoking thought frequency and cravings
Carciofo et al. 2015; Psychometric evaluation of Chinese‐language 44‐item and 10‐item big five personality inventories, including correlations with chronotype, mindfulness and mind wandering	2496 participants (aged 18–82)	To evaluate the psychometric properties of Chinese‐language BFI‐44 and BFI‐10 personality scales and examine correlations with chronotype, mindfulness, and mind wandering	Big Five personality traits, chronotype, mindfulness, mind wandering	self‐report scales
Green et al. 2016; Trauma‐related versus positive involuntary thoughts with and without meta‐awareness	87 subjects from Flinders University (aged 18–38)	To investigate the frequency and characteristics of trauma‐related vs. positive involuntary thoughts and meta‐awareness	Involuntary thoughts, meta‐awareness, trauma, positive events, self‐caught thoughts, probe‐caught thoughts	reported involuntary thoughts and responded to probes during a reading task; thoughts classified as self‐caught or probe‐caught
Deng et al. 2016; The role of mindfulness and self‐control in the relationship between mind‐wandering and metacognition | not specified	434 Chinese college students (98 females; mean age 19.55)	To explore the relationship between mind wandering and metacognition, and the potential mediating effects of self‐control and mindfulness	Mind wandering, metacognition, mindfulness, self‐control	multiple questionnaires: Mind‐Wandering Questionnaire (MWQ), Metacognitions Questionnaire (MQ), Mindfulness Attention and Awareness Scale (MAAS), and Tangney's Self‐control Scale (SCS)
Tan et al. 2015; Mind wandering and the incubation effect in insight problem solving	91 Chinese college students (42 women, 49 men; mean age 22.5)	To assess whether insightful solutions are related to mind wandering during the incubation stage of the creative process	Insightful solutions, mind wandering, number reduction task (NRT), creativity	sustained attention response task (SART); overall creativity, working memory capacity, motivation, and daydreaming frequency
Seli et al.; Motivation, intentionality, and mind wandering: implications for assessments of task‐unrelated thought	166 undergraduate students from the University of Waterloo	To explore the relationship between motivation, intentionality, and mind wandering during task performance	Task‐unrelated thoughts (TUTs), motivation, intentional TUTs, unintentional TUTs, performance decrements	Participants performed a sustained‐attention task (MRT) and reported their motivation levels; Rhythmic‐Response Times (RRTs) calculated for each trial; participants asked about their motivation to perform well on the task; analysis of intentional vs. unintentional TUTs and their relation to performance
Hollander and Huette 2022; Extracting blinks from continuous eye‐tracking data in a mind wandering paradigm	41 students (right‐handed native English speakers)	To investigate the relationship between mind wandering and blink durations across multiple task modalities investigate the relationship between mind wandering and blink durations across multiple task modalities and to provide recommendations for accurately deriving blink events from continuous eye‐tracking data	Blink characteristics, mind wandering, eye‐tracking, stimulus/task engagingness	Participants completed experiments online using their own computers; eye movements recorded; reading tasks varied in engagingness; tasks included articles on Salem witch trials, law and punishment in Plymouth colony, and the Constitution of the United States; readings presented one page at a time; blink durations measured and analyzed
Zukosky and Wang 2021; Spontaneous state alternations in the time course of mind wandering	Not specified | Experiment 1: Not specified; Experiment 2: eight undergraduate students from University of Illinois (aged 18–22)	To investigate the dynamics of shifting between focus and mind wandering, challenging the two‐stage model	Mind wandering, focus state, meditation, probe‐caught method, self‐caught method	self‐caught and probe‐caught methods to assess mind wandering
Wong et al. 2022; Spontaneous mind‐wandering tendencies linked to cognitive flexibility in young adults	79 university students (69 females, 15 males) from the University of Otago	To investigate the relationship between spontaneous mind wandering tendencies and cognitive flexibility	Mind wandering, cognitive flexibility, task‐switching performance	Attentional Control Scale (ACS), Mind Wandering: Deliberate (MW‐D) and Spontaneous (MW‐S) Scales, Awareness of Self‐Initiated Task‐Switches, and Dundee Stress State Questionnaire (DSSQ)
Figueiredo et al. 2018; Transcultural adaptation to Portuguese of the Mind Excessively Wandering Scale (MEWS) for evaluation of thought activity	40 adults (20 with ADHD, 20 normal controls) from Hospital Copa D'Or in Rio de Janeiro	To describe the cross‐cultural adaptation of the Mind Excessively Wandering Scale (MEWS) to Brazilian Portuguese	Mind wandering, ADHD, frequency, intensity, negative outcomes	the Mind Excessively Wandering Scale (MEWS)
Barron et al. 2011; Absorbed in thought: the effect of mind wandering on the processing of relevant and irrelevant events	25 right‐handed adults (16 female, nine male; mean age 27.84)	To explore whether mind wandering (TUT) is due to general distraction, task‐relevant processing deficits, or general reduction in attention	Task‐unrelated thought (TUT), cortical processing, sensory information	Participants performed a visual oddball task; differentiated between rare target stimulus, rare novel stimulus, and frequent nontarget stimulus; TUT measured using a validated retrospective measure; EEGs recorded from 32 channels
Jha et al. 2017; Practice is protective: mindfulness training promotes cognitive resilience in high‐stress cohorts	55 US Marine Corps reservists (31 MT, 24 MC)	To investigate if mindfulness training (MT) promotes cognitive resilience by curbing attentional lapses in high‐stress cohorts	Attentional performance, mind wandering, mindfulness training (MT), Sustained Attention to Response Task (SART)	MT group attended 8‐week MMFT course; SART used to index objective attentional performance and subjective ratings of mind wandering before (T1) and after (T2) the MT course; changes in SART measures analyzed for correlation with MT practice
Uzzaman 2011; The use of eye movements as an objective measure of mind wandering | not specified	33 undergraduate students from the University of Toronto Scarborough	To investigate whether eye movement behavior differs when reading for comprehension vs. experiencing a mind wandering episode	Mind wandering, eye movements, reading comprehension	Reading task, Eye movements tracked and recorded, Probed randomly every 2–3 min, Mind wandering indication, Eye movement behavior analyzed, Fixation duration, Blink count, Pupil size
Andrillon et al. 2021; Predicting lapses of attention with sleep‐like slow waves	Thirty‐two (*N* = 32) healthy adults recruited. 26 participants included in analyses (age: 29.8 ± 4.1 years; 10 females)	To understand the neural mechanisms underlying attentional lapses by studying behavior, subjective experience, and neural activity of healthy participants performing a task	High‐density electroencephalography (EEG)	Experimental design with modified SARTs for Face and Digit tasks
Gonçalves et al. 2018; Neuromodulating attention and mind‐wandering processes with a single session real time EEG	Thirty healthy college students (21 women, nine men) with normal or corrected to normal vision participated in the study (age: 18–32 years; M = 20.7, SD = 3.7)	To test the effects of two distinct single‐session real‐time EEG (rtEEG) protocols on external attention (EA) and mind wandering (MW) processes	Attention Network Task (ANT), Thought Identification Task (TIT), Resting State Questionnaire (ReSQ), MW Intentionality scales (MW‐D/S), Visual Analogue Scale (VAS)	Random group design with rtEEG training protocols
Eusebio 2016; Mind wandering differences between younger and older adults: a new neurocognitive framework	Sample of 22 older adults (69.7 ± 5.2 years; 10 males) and 27 younger adults (25.4 ± 3.1 years; 11 males) recruited through the Rotman Research Institute subject pools.	To behaviorally test a new neurocognitive framework for mechanisms behind age‐related differences in mind wandering.	Commission errors, response time (RT) patterns, and ex‐Gaussian model parameters (mu, sigma, tau)	Participants performed the sustained attention to response task (SART) in an MRI scanner, with RT patterns analyzed using an ex‐Gaussian model.
Antonova et al. 2022; EEG microstates: functional significance and short‐term test–retest reliability	Twenty participants (16 males, mean age = 31.5, standard deviation = 12.5) recruited via university circular emails and local online forums.	To investigate the functional significance of the canonical EEG microstate classes and their pairwise transitions, and to establish their within‐session test–retest reliability.	Duration and coverage of EEG microstates, transition probabilities, subjective ratings of alertness, and sense of effort.	Recorded 36‐channel EEGs during three eyes‐closed conditions: mind wandering, verbalization (silently repeating “square”), and visualization (visualizing a square). Analyzed EEG data
Gmehlin et al. 2016; Attentional lapses of adults with attention deficit hyperactivity disorder in tasks of sustained attention	Total of 48 adult participants (24 with ADHD, 24 healthy controls) aged 19–63 years (M = 34.1 years; SD = 12.3 years).	To analyze basic deficits in sustaining attention and their relation to more complex attentional dysfunctions in adults with ADHD.	Ex‐Gaussian parameters, self‐report scales for ADHD symptoms, intellectual functions, sustained alertness, selective and divided attention	Tests of sustained alertness (WAF‐A), selective attention (WAFS), and divided attention (WAFG) using the Vienna Test System (VTS).
Unsworth and Robison 2018; Tracking arousal state and mind wandering with pupillometry	165 participants aged 18–35 years, recruited from the University of Oregon subject pool.	To examine the association between arousal state and different mind wandering states using pupillometry.	Thought probes, psychomotor vigilance task, Stroop task, eye tracking.	Sustained attention task with continuous pupil response recording and periodic thought probes to assess on‐task and mind wandering states.
Denkova et al. 2019; Dynamic brain network configurations during rest and an attention task with frequent occurrence of mind wandering	Forty‐six healthy adults (30 women; M age = 31.22, SD = 11.51) with normal or corrected‐to‐normal vision.	To investigate the dynamic functional connectivity (DFC) of large‐scale brain networks during rest and tasks with frequent mind wandering.	Functional connectivity, frequency of DFC states during rest and task, MRI scans.	Neuroimaging data from resting‐state scan followed by two scans of sustained attention to response task (SART) with embedded probes indicating high prevalence of mind wandering.
Kruger et al. 2021; Contrasting mind‐wandering, (dark) flow, and affect during multiline and single‐line slot machine play	110 participants (56 female, 53 male, one nonbinary) aged 22–82 years (mean age = 59.93 years, SD = 13.46) recruited from Elements Casino in Brantford, Ontario, Canada.	To assess differences in mind wandering, dark flow, and affect during multiline and single‐line slot machine play, and their relationship to problem gambling severity.	Mindfulness (MAAS), gambling problems (CPGI), depressive symptoms (DASS‐21), boredom proneness (BPS), dark flow, affect, thought probes	thought probes
Gonçalves et al. 2017; Mind wandering and task‐focused attention: ERP correlates	Thirty‐three healthy individuals (22 female) aged 18–40 years (mean age = 23.45 years, SD = 5.01).	To test if individuals predominantly focusing on mind wandering or focused attention show distinct cortical processing during the Attention Network Task (ANT).	Event‐related potentials (ERPs) such as pN1, pP1, P1, N1, pN, and P3.	EEG high‐density acquisition while performing the ANT, with MW assessed using an adapted version of the Resting State Questionnaire (ReSQ).
Morrison et al. 2014; Taming a wandering attention: short‐form mindfulness training in student cohorts	58 healthy University of Miami students (30 female) with a mean age of 18.20 years (SD = 1.29).	To examine the benefits of short‐form mindfulness training (MT) in reducing mind wandering and improving working memory in University students.	Sustained Attention to Response Task (SART), operation span, delayed‐recognition with distracters.	SART and working memory tasks
Jamshidi et al. 2020; Effectiveness of EMDR therapy on post‐traumatic stress symptoms, mind‐wandering, and suicidal ideation in Iranian child abuse victims	Thirty female victims of child abuse (aged 18–30 years) living in the Welfare Organization Center of Shiraz, Iran.	To investigate the effectiveness of EMDR therapy on reducing PTSD symptoms, suicidal ideations, and mind wandering in female victims of child abuse.	Civilian Mississippi Scale for PTSD (CMS), Child Abuse and Self‐Report Scale (CASRS), Mind‐Wandering Questionnaire (MWQ), Beck Scale for Suicidal Ideation (BSSI), Brief Dissociative Experiences Scale (DES‐B)	EMDR sessions
Jin et al. 2020; Decoding study‐independent mind‐wandering from EEG using convolutional neural networks	Thirty participants (13 females, ages 18–30 years, M = 23.33, SD = 2.81) in the training dataset and 30 participants (16 females, ages 18–31 years, M = 23.73, SD = 3.47) in the testing dataset.	To train EEG classifiers using convolutional neural networks (CNN) to track mind wandering across studies.	Raw EEG band‐frequency information, single‐trial ERP (stERP) patterns, connectivity matrices between channels (ISPC).	Training CNN models for each input type from each EEG channel as the input model for the meta‐learner, using leave‐N‐participant‐out cross‐validations and testing on independent study data.
Mooneyham et al. 2017; States of mind: characterizing the neural bases of focus and mind‐wandering through dynamic functional connectivity	Thirty‐eight college undergraduates (16 men and 22 women; mean age = 20.38 years, SD = 2.28 years) from the University of California, Santa Barbara.	To characterize the neural bases of focus and mind wandering using dynamic functional connectivity during tasks requiring sustained attention to the sensations of breathing.	Functional connectivity between regions of the executive control, salience, and default networks.	Dynamic functional connectivity approach using a sliding window during a mindful breathing scan, with pretesting and post‐testing, MRI data processing, and ROI selection.
Belardi et al. 2022; On the relationship between mind wandering and mindfulness	Total sample of 541 participants: 313 German‐speaking unpaid participants (GUP) aged 16–85 (M = 38.78, SD = 12.95) and 228 English‐speaking paid participants (EPP) aged 19–68 (M = 34.27, SD = 11.39).	To examine the association between mind wandering (MW) and mindfulness, and evaluate the psychometrics of measures often used to quantify them.	Mindful Attention Awareness Scale (MAAS), Sustained Attention to Response Task (SART), self‐reported MW, meta‐awareness of MW (experience sampling probes).	Online experiment with MAAS and SART, using ES probes to record self‐reports of MW and meta‐awareness, analyzed for internal consistency and reliability.
Macaulay et al. 2024; Examining the facets of mindful engagement and mind wandering in nature	215 participants (mostly university students, 74% female, mean age = 24.3 years, SD = 9.4).	To examine the facets of mindfulness and mind wandering in nature and their associations with psychological restoration and nature connection.	State mindfulness (SMS, MSMQ, TMS), state mind wandering (MW‐D subscale, state version of MAAS), positive and negative affect, perceived restorativeness.	Participants engaged in a 20‐min outdoor experience with different engagement instructions, completing online surveys and the Sustained Attention to Response Task (SART) before and after.
Van Opstal et al. 2022. Mind‐wandering in larks and owls: the effects of chronotype and time of day on the frequency of task‐unrelated thoughts	130 participants (mean age: 20.65 years, range: 18–38; 96 females, 33 males, 1 nonbinary) classified as Morning, Intermediate, and Evening types.	To investigate the synchrony effect in the frequency of mind wandering (MW) based on chronotype and time of day.	Morningness–Eveningness Questionnaire (MEQ), Sustained Attention to Response Task (SART), thought probes, accuracy, reaction time coefficient of variance.	Participants completed the SART twice, once in the morning and once in the evening, with MW measured using a probe‐caught method and analyzed for the synchrony effect and the relation to sleep‐related factors.
van Son et al. 2019b; Electroencephalography theta/beta ratio covaries with mind wandering and functional connectivity in the executive control network	26 participants who performed a 40‐min breath‐counting task and reported mind wandering episodes while EEG was measured and again during MRI.	To test if mind wandering‐related fluctuations in theta/beta ratio (TBR) covary with functional variation in executive control network (ECN) and default mode network (DMN) connectivity.	Frontal resting‐state EEG, TBR, MW episodes, ECN and DMN connectivity.	Continuous EEG and MRI scans during a breath‐counting task, with MW episodes reported by participants and analyzed for TBR and functional connectivity variations.
Kam and Mickleborough 2014; Migraine and attention to visual events during mind wandering	28 participants: 14 in the migraine group (11 women; mean age = 24.4, SD = 5.1) and 14 in the nonmigraine control group (eight women; mean age = 21.6, SD = 2.9).	To examine whether cortical hypersensitivities in migraineurs extend to mind wandering by analyzing ERP responses during a sustained attention to response task (SART).	Event‐related potentials (ERPs) such as P3 component, EEG data, attentional state reports (on‐task vs. mind wandering).	SART performance with EEG recorded from 64 active electrodes, ERP responses to task‐relevant stimuli analyzed based on attentional state reports, data processed using ERPLAB and EEGLAB.
Marcusson‐Clavertz et al. 2023; Mind wandering and sleep in daily life: a combined actigraphy and experience sampling study	202 participants (139 from Lund, Sweden, and 63 from Mannheim, Germany; mean age = 23.95 years, SD = 5.11; 37% males, 63% females)	To examine the relations between several features of sleep (duration, fragmentation, disturbances) and mind wandering (task‐unrelated, stimulus‐independent, unguided thoughts) using ambulatory assessments.	Patient‐reported outcomes measurement information system (PROMIS) sleep disturbance scale, Short Imaginal Processes Inventory (SIPI), actigraphy, experience sampling.	Participants wore a wristband device that collected actigraphy and experience‐sampling data across 7 days and 8 nights, with signal‐contingent beep questionnaires administered 10 times per day.
Jonkman et al. 2017; Mind wandering during attention performance: effects of ADHD‐inattention symptomatology, negative mood, ruminative response style, and working memory capacity	90 college students: 46 with high ADHD‐Inattention symptomatology (11 men, 35 women) and 44 with low ADHD‐Inattention symptomatology.	To investigate the effects of dysphoric mood on task‐unrelated mind wandering and its consequences on cognitive task performance in college students with high or low ADHD‐Inattention symptomatology.	Self‐reported mind wandering frequency, Sustained Attention to Response Task (SART), reading task, ruminative response style, working memory capacity.	Mood induction (negative or positive), mind wandering frequency measured during SART and reading task, with additional measures of ruminative response style and working memory capacity.
Zawadzki et al. 2024; For whom is mind wandering stressful: the moderating role of dispositional emotionality and personality in predicting emotional experiences in everyday life	264 participants (aged 19–73 years, average age = 40.73 years, SD = 11.93; 137 males, 127 females).	To test whether dispositional emotionality and personality traits moderate the emotions experienced when engaging in mind wandering.	State‐Trait Anger Scale, NEO Five Factor Inventory, ecological momentary assessment (EMA) of mind wandering and emotional states.	Participants completed measures of dispositional emotionality and personality, and then a 24‐h EMA protocol responding every 30 min about mind wandering and emotional states.
Banks and Boals 2016; Understanding the role of mind wandering in stress‐related working memory impairments	150 undergraduates (84 females; mean age = 21.28 years) from the University of North Texas.	To examine the impact of mind wandering on working memory (WM) task performance following laboratory stressors and explore the role of thought suppression.	Automated Operation Span Task (AOSPAN), Automated Reading Span Task (RSPAN), probe‐caught task‐unrelated thoughts (TUTs), self‐reported percentage of TUTs, Daily Inventory of Stressful Events (DISE), Life Experiences Scale (LES), Impact of Events Scale	WM measures before (Time 1) and after (Time 2) a writing manipulation to induce mind wandering regarding negative, positive, or neutral events, and subsequent measurement of mind wandering and WM task performance.
			(IES), White Bear Suppression Inventory (WBSI).	
Madiounia et al. 2020; Mind‐wandering and sleepiness in adults with attention‐deficit/hyperactivity disorder	25 adults with ADHD recruited from the Adult ADHD outpatient clinic of the Gui de Chauliac Hospital, Montpellier, France, and a control group of 28 participants with no history of neurological or psychiatric disorders.	To investigate the relationship between mind wandering, sleepiness, and ADHD symptoms in adults.	SART with embedded thought‐probes, Karolinska Sleepiness Scale (KSS), Adult ADHD Self Report Scale v1.1 (ASRS), Epworth Sleepiness Scale (ESS), Daydreaming Frequency Scale (DDFS), Thought Characteristics Questionnaire (TCQ).	SART with embedded thought‐probes and KSS, self‐report questionnaires assessing ADHD symptoms, sleepiness, mind wandering, and daydreaming, conducted in accordance with the Declaration of Helsinki.
Lu and Rodriguez‐Larios 2022; Nonlinear EEG signatures of mind wandering during breath focus meditation	25 participants (age 23.46 years, range 20–29 years, 11 males) with no previous meditation experience.	To assess whether nonlinear EEG signatures can be used to characterize mind wandering during breath focus meditation in novice practitioners.	EEG complexity metrics (Higuchi's fractal dimension (HFD), Lempel‐Ziv complexity (LZC), Sample entropy (SampEn)), experience sampling reports.	Breath focus meditation with eyes closed, interrupted by bell sounds to report mind wandering or breath focus states, EEG recorded and analyzed for complexity metrics using HFD, LZC, and SampEn.
Helfer et al. 2019; The effects of emotional lability, mind wandering and sleep quality on ADHD symptom severity in adults with ADHD	81 English‐speaking adults with ADHD (60 male, 51 female; mean age 32.4 years, SD 10 years, mean IQ 110, SD 13) recruited via South London and Maudsley Adult ADHD Outpatient Services.	To examine the influence of mind wandering, sleep quality, and emotional lability on ADHD symptom severity using serial multiple mediation models.	Conners' Adult ADHD Rating Scales (CAARS), Affective Lability Scale (ALS), Mind Excessively Wandering Scale (MEWS), Pittsburgh Sleep Quality Index (PSQI).	Serial multiple mediation models to examine the relationship between mind wandering, sleep quality, emotional lability, and ADHD symptom severity
Simor et al. 2024; Reduced REM and N2 sleep, and lower dream intensity predict increased mind‐wandering	67 healthy participants (47 females; mean age = 24.7, Std. Dev = 3.3, range = 19–33) recruited through flyers at Université Libre de Bruxelles, Belgium.	To examine if objective sleep parameters predict the tendency for increased mind wandering on the following day using mobile sleep EEG headbands and self‐report scales.	Sleep EEG (Dreem2 headband), Groningen Sleep Quality Scale (GSQS), self‐reports on mind wandering and dream intensity.	Sleep EEG assessments with Dreem2 headband for 7 consecutive nights, self‐report scales on sleep quality and mind wandering, data collected and analyzed for correlations between sleep stages and mind wandering.
Groot et al. 2021; Probing the neural signature of mind wandering with simultaneous fMRI‐EEG and pupillometry	28 healthy adult volunteers (25 female, aged 21 ± 2.51 years) recruited from the University of Amsterdam	To investigate the neural mechanisms underlying task‐unrelated thoughts (TUTs) using simultaneous fMRI, EEG, and pupillometry.	fMRI, EEG, pupillometry, experience sampling probes, reaction time coefficient of variability (RTCV), omission error rate, commission error rate.	Simultaneous fMRI‐EEG and pupillometry during a sustained attention to response task (SART) with experience sampling probes, features extracted and analyzed using a supervised learning algorithm.
Martínez‐Pérez et al. 2023; Vigilance decrement and mind‐wandering in sustained attention tasks: two sides of the same coin?	78 participants (65 females; mean age = 19.8 years) undergraduates at the University of Murcia.	To investigate whether processes leading to vigilance decrement and mind wandering (MW) share a common mechanism or can be dissociated in sustained attention tasks.	SART performance, thought probes on intentional and unintentional MW, pre–post resting EEG, working memory capacity (symmetry span and rotation span tasks).	Random assignment to factorial combination of task demand (low, high) and stimulation (anodal HD‐tDCS, sham), SART performance with thought probes, pre–post resting EEG, analysis of alpha oscillations.
Günay Aksoy et al. 2022; Linguistic equivalence, validity and reliability study of the mind excessively wandering scale	64 patients previously diagnosed with adult ADHD, 60 students for transliteral equivalence, and 80 healthy controls for test–retest reliability.	To study the linguistic equivalence, validity, and reliability of the Turkish version of the Mind Excessively Wandering Scale (MEWS).	Mind Excessively Wandering Scale (MEWS), Adult Attention Deficit Hyperactivity Disorder Self‐Report Scale (ASRS‐v1.1), Barratt Impulsiveness Scale (BIS‐11), Difficulties in Emotion Regulation Scale (DERS).	Transliteral equivalence, validity, and reliability stages conducted with various study groups, confirmatory factor analysis, and test–retest reliability analysis.
Bozhilova et al. 2020; Electrophysiological modulation of sensory and attentional processes during mind wandering in attention‐deficit/hyperactivity disorder	23 adults with ADHD and 25 controls who met quality control criteria for electroencephalography (EEG) data.	| To compare adults with ADHD and controls on event‐related potentials of early sensory processes (P1) and attention allocation (P3) during tasks manipulating cognitive demands and periods of mind wandering (MW) and task focus.	EEG data, event‐related potentials (P1 and P3), mind wandering frequency, cognitive task performance.	Cognitive tasks (Mind Wandering Task, Sustained Attention Task) with simultaneous EEG recordings, diagnostic interview for ADHD, self‐report questionnaires, IQ testing, analysis of EEG data.
Miś and Kowalczyk 2020; Mind‐wandering during long‐distance running and mood change: the role of working memory capacity and temporal orientation of thoughts	53 amateur runners (25 women, 28 men) aged 18–54 years (M = 33.7, SD = 10.25) recruited through social media and personal contacts.	To explore determinants and consequences of mind wandering during long‐distance running training, focusing on working memory capacity (WMC) and temporal orientation of thoughts.	Runner's Thoughts Questionnaire (RTQ), UWIST Mood Adjective Checklist (UMACL), Operation Span Task (OSPAN), Task‐Unrelated Thoughts Questionnaire (TUTQ).	40‐min outdoor run at a comfortable pace, prerun and postrun mood assessment using UMACL, retrospective questionnaire on thoughts during training, assessment of WMC and individual tendencies in mind wandering.
Seli et al. 2018; How pervasive is mind wandering, really?	239 undergraduate students randomly assigned to either the dichotomous (*n* = 122) or multilevel (*n* = 117) probe condition.	To investigate how mind wandering estimates vary depending on the response options provided and the assumptions about introspective judgments.	Dichotomous thought probes, multilevel thought probes.	Experience sampling with thought probes via MetricWire smartphone application, probing participants 10 times per day for 7 days, comparing dichotomous and multilevel probe conditions.
Kawagoe et al. 2018; Different pre‐scanning instructions induce distinct psychological and resting brain states during functional magnetic resonance imaging	30 healthy subjects (18 females and 12 males; mean age: 22.9 years, SD: 2.2 years).	To investigate whether different instructions affect mental state and functional connectivity (FC) during rs‐fMRI scanning.	Functional connectivity (FC) between brain networks, self‐reported mental state.	rs‐fMRI scans under two conditions (think of nothing (TN) and mind wandering (MW)), self‐reported mental state, independent component analysis, functional network connectivity analyses.
Anderson and Farb 2018; The metronome counting task for measuring meta‐awareness	74 young‐adult undergraduates from the University of Toronto Mississauga campus.	To develop and validate the Metronome Counting Task (MCT) as a tool for dynamically measuring meta‐awareness and detecting meta‐awareness loss.	Metronome Counting Task (MCT) performance, Attention Related Cognitive Errors scale, self‐rated performance, motivation, demographic variables.	Continuous performance task with tapping to a steady beat, self‐caught meta‐awareness failures, measurement of response variability, self‐reported motivation and performance.
Chen et al. 2022; The different relationship pattern between mind wandering and daily prospective memory failure in individuals with high and low schizotypal traits	26 participants (17 females) in the high schizotypal group, and 29 participants (25 females) in the low schizotypal group	To investigate the relationship between mind wandering (MW) and daily prospective memory (PM) failure in individuals with high and low schizotypal traits.	Schizotypal Personality Questionnaire, PM subscale of Prospective and Retrospective Memory Questionnaire, thought‐sampling task embedded in modified SART.	Screening using Schizotypal Personality Questionnaire, 16 blocks of modified SART with thought probes, participants indicated temporal direction of MW episodes, analysis of MW and PM failure association.
Hung et al. 2020; A hypothesis‐generating study using electrophysiology to examine cognitive function in colon cancer patients	10 participants tested at baseline (within 3 weeks of starting chemotherapy), 6 months, and 12 months.	To describe the trajectory of cognitive function using neuropsychological tests and electrophysiological measures in individuals receiving 5FU/oxaliplatin chemotherapy for colon cancer.	Neuropsychological tests, electrophysiology recordings of P300 event‐related potential (ERP), SART paired with experience sampling of attentional states (on‐task vs. mind wandering).	Neuropsychological tests and EEG recordings at three timepoints, analysis of P300 ERP amplitudes as a function of attentional states, and comparison with mean neuropsychological test performance.
88 undergraduate students from a medium‐sized private US university (*N* = 65) and a large public US university (*N* = 23).	88 undergraduate students from a medium‐sized private US university (*N* = 65) and a large public US university (*N* = 23).	To test the idea that semantically rich stimuli yield patterns of mind wandering closely coupled with the stimuli compared to being more internally triggered.	Self‐reported mind wandering, latent semantic analysis of thought content.	Participants read an instructional text and watched a film for 20 min each, reporting mind wandering whenever they zoned out, analysis of thought content and triggers.
Hardikar et al. 2022; Macro‐scale patterns in functional connectivity associated with ongoing thought patterns and dispositional traits	144 participants (74 men, mean age = 26.77 years, SD = 4.03; 70 women, mean age = 26.93 years, SD = 5.55).	To explore the relationship between macro‐scale brain activity patterns, ongoing thought patterns, and dispositional traits during wakeful rest.	Self‐reported personality measures, multidimensional experience sampling (MDES), resting‐state fMRI (rs‐fMRI).	Participants completed self‐reports on personality and thought patterns, underwent rs‐fMRI scans, and completed MDES surveys immediately after each 15 min scan, analysis of cortical gradients and functional connectivity.
Kucyi et al. 2021; Prediction of stimulus‐independent and task‐unrelated thought from functional brain networks	Multiple datasets including healthy adults and adults with ADHD. Total *n* = 1115.	To develop and test the generalizability, specificity, and clinical relevance of a functional brain network‐based marker for stimulus‐independent, task‐unrelated thought (SITUT).	fMRI, experience sampling, trait‐level measures, structural and resting‐state functional MRI (rs‐fMRI).	Combined fMRI with online experience sampling, developed connectome‐wide model of inter‐regional coupling predicting SITUT fluctuations, tested generalizability in independent sample of adults with ADHD, assessed further prediction in three additional resting‐state fMRI studies.
Hsu et al. 2020; Spontaneous thought‐related network connectivity predicts sertraline effect on major depressive disorder	22 drug‐naïve MDD patients and 35 normal controls (NC), matched for gender, age, education, and handedness.	To investigate whether the functional integrity of cortical networks associated with spontaneous thoughts is modulated by sertraline treatment and to predict post‐treatment effects based on pretreatment rsFC.	Resting‐state functional connectivity (rsFC) in spontaneous brain networks, 17‐item Hamilton Depression Rating Scale (HAM‐D).	MDD patients received sertraline treatment, MRI scans conducted at baseline and after 6 weeks of treatment, analysis of rsFC and its predictive power for sertraline treatment outcome.
Smeekens and Kane 2016; Working memory capacity, mind wandering, and creative cognition: an individual‐differences investigation into the benefits of controlled versus spontaneous thought	Undergraduate students from the University of North Carolina at Greensboro.	To test whether executive control, indicated by working memory capacity (WMC) and mind wandering propensity, helps or hinders creativity.	WMC assessed by complex span tasks, mind wandering assessed by probed reports of task‐unrelated thought (TUT), retrospective self‐reports of Openness, mind wandering, and daydreaming propensity.	Incubation periods inserted into divergent thinking tasks, mind wandering measured by thought probes during cognitive tasks (SART and n‐back), analysis of WMC and creativity correlation.
Figueiredo et al. 2020; Mind‐wandering, depression, anxiety and ADHD: disentangling the relationship	78 adolescents (53 males and 25 females) comprising ADHD, clinical controls, and typically developing individuals.	To investigate the role of anxiety and depression in mind wandering (MW) in patients with ADHD.	Mind Excessively Wandering Scale (MEWS), SCARED, CDI, SNAP‐IV, IQ, age.	Correlational analysis, multiple linear regression analysis, group comparisons (ADHD vs. non‐ADHD, Anxiety vs. Non‐Anxiety)
Ottaviani and Couyoumdjian 2013; Pros and cons of a wandering mind: a prospective study	40 subjects (21 women, 19 men; mean age = 24.5 years) recruited among students at Sapienza University of Rome	To investigate whether proneness to mind wandering (MW) was prospectively associated with negative health outcomes.	Electrocardiogram (ECG), thought probes during tracking task, personality questionnaires, Ecological Momentary Assessment (EMA).	Laboratory session at time 0 with baseline ECG, tracking task with thought probes, personality questionnaires; follow‐up at time 1 with 24‐h EMA, ambulatory ECG recording, and electronic diaries.
Stawarczyk et al. 2020; Drowsiness or mind‐wandering? Fluctuations in ocular parameters during attentional lapses	33 participants (25 women; mean age = 21.76 years) from the University of Liège.	To assess fluctuations in mind wandering and sleepiness during a sustained attention task while ocular parameters were recorded, and determine whether variations are related to mind wandering, drowsiness, or both.	Eyeblink frequency, pupil dynamics, mind wandering, sleepiness, SART performance.	Participants performed an adaptation of the SART with embedded thought probes and KSS, ocular parameters recorded, analysis of fluctuations in mind wandering and sleepiness, comparison of effects on task performance.
Ottaviani and Couyoumdjian 2022; Regulating test anxiety by joy: based on the Mutual Promotion and Mutual Counteraction (MPMC) theory of affect	Study 1: 95 participants (45 males, 21.27 ± 2.27 years old on average); Study 2: 42 participants (24 males, 21.05 ± 2.39 years old on average).	To verify the effectiveness of a new emotion regulation strategy called “joy counteracts fear” (JCF) in alleviating test anxiety (TA).	Mood Induction Task (MIT), improved Sustained Attention to Response Task (SART), Test Anxiety Scale (TAS), Positive and Negative Affect Scale (PANAS).	Study 1: Mood induction and improved SART conducted to explore TA's influence on mind wandering (MW); Study 2: Verification of JCF strategy effectiveness by comparing pre‐ and postintervention states and MIT, SART with comedy clips, PANAS scores.
Krasich et al. 2018; Gaze‐based signatures of mind wandering during real‐world scene processing	51 participants (Mage = 19 years, SD = 1.1 years, 37 female) from a private, selective, Midwest university.	To investigate gaze allocation during mind wandering (MW) and attentively viewing real‐world scenes.	Eye movements (fixations, fixation dispersion, eyeblinks), self‐reported MW via thought probes.	Participants studied images of urban scenes and responded to thought probes on MW, followed by a two‐alternative forced‐choice recognition task, eye movements recorded during scene viewing phase.
Liu et al. 2023; High‐mind wandering correlates with high risk for problematic alcohol use in China and Germany	1123 participants from China and 1018 participants from Germany, general population over 18 years of age.	To investigate if mind wandering is associated with a risk of developing problematic alcohol use in the general population of China and Germany.	Mind Wandering Questionnaire (MWQ), Alcohol Use Disorders Identification Test (AUDIT).	Cross‐sectional online survey assessing mind wandering and problematic alcohol use, conducted from December 2021 to February 2022, recruited through social media, advertisements, newsletters, and Prolific platform.
Tomescu et al. 2022; Spontaneous thought and microstate activity modulation by social imitation	43 participants (24 men, 19 women; mean age = 25.7 years).	To investigate how social imitation modulates spontaneous thoughts and resting‐state EEG activity, and its correlation with behavioral states and personality traits.	EEG microstate analysis, Amsterdam Resting‐State Questionnaire 2.0 (ARSQ), Neo Personality Inventory‐Revised (NEO PI‐R), self‐reported stress and well‐being levels.	Participants performed social imitation (SI) and control (CTRL) tasks on two separate days, EEG recorded during resting state, self‐reported data collected, analysis of EEG, ARSQ, and NEO PI‐R.
Jaswal et al. 2019; The influence of acetaminophen on task related attention	40 participants (26 females; M = 23.4 years old, SD = 6.61; 39 right‐handed) from the community.	To examine whether acetaminophen impacts off‐task attentional states, such as mind wandering, during a sustained attention to response task (SART).	Event‐related potentials (ERPs), P300 and LPP ERP components, attentional reports (on‐task vs. off‐task).	Placebo‐controlled between‐groups design, participants performed SART while ERPs to target events were recorded, queried for attentional reports at random intervals, analysis of ERP amplitudes and attentional states.
Anderson and Farb 2020; The metronome response task for measuring mind wandering: replication attempt and extension of three studies by Seli et al.	300 undergraduate participants recruited from three testing sites: University of Toronto Mississauga campus (UTM), University of Toronto Scarborough campus (UTSC), and York University campus.	To replicate three studies that used the Metronome Response Task (MRT) to measure mind wandering and examine differences between intentional and unintentional mind wandering.	Metronome Response Task (MRT), thought probes, motivation, confidence, Attention‐Related Cognitive Errors scale, demographic variables, extension questions.	Participants performed MRT by tapping along to a steady aural beat, thought probes presented randomly, self‐reported attentional state, motivation, confidence, and additional constructs like boredom and effort were measured.
Compton et al. 2019; The wandering mind oscillates: EEG alpha power is enhanced during moments of mind‐wandering	62 undergraduates (33 female, 25 male, four other/unreported gender) from Haverford College.	To test whether episodes of mind wandering during a demanding cognitive task are associated with increases in EEG alpha power.	EEG alpha power, self‐reported mind wandering, six‐choice Stroop task performance.	Participants completed a six‐choice Stroop task with more than 800 trials while EEG was recorded, intermittent experience‐sampling probes asked participants to report their mind wandering state, analysis of EEG alpha power preceding probes.
Moukhtarian et al. 2020; Wandering minds in attention‐deficit/hyperactivity disorder and borderline personality disorder	98 females aged 18–65 years recruited from ADHD and borderline personality outpatient clinics and volunteer databases.	To investigate excessive spontaneous mind wandering (MW‐S) in daily life across ADHD, BPD, comorbid ADHD+BPD, and control groups.	Mind Excessively Wandering Scale (MEWS), Brief Symptom Inventory (BSI), Wechsler Abbreviated Scale of Intelligence—Second edition (WASI‐II), experience sampling via MoodMapper.	Participants completed self‐reported MW‐S assessments using MEWS, experience sampling of MW‐S carried out eight times daily over 5 days using MoodMapper app, BSI for comorbid depression and anxiety, IQ assessment using WASI‐II.
Kawashima and Kumano 2017; Prediction of mind‐wandering with electroencephalogram and non‐linear regression modeling	50 participants recruited from Waseda University, aged 21.77 (SD = 2.27) years, 21 males and 22 females, all right‐handed.	To investigate the use of electroencephalogram (EEG) variables and nonlinear regression modeling to predict the intensity of mind wandering (MW).	EEG power and coherence value, Center for Epidemiologic Studies Depression Scale (CES‐D).	Participants performed a Sustained Attention to Response Task (SART) with thought sampling probes, EEG data recorded, MW rated on a 7‐point Likert scale, analysis of EEG variables and application of Support Vector Machine Regression (SVR) models.
Jang et al. 2020; Detecting mind‐wandering from eye movement and oculomotor data during learning video lecture	24 preservice teachers (16 females and eight males) with an average age of 23.5 years.	To detect mind wandering experienced by preservice teachers during a video learning lecture on physics using oculomotor data and eye movements.	Oculomotor data (blink count, pupil size), eye movements (saccade velocity, amplitude, count, fixation duration, fixation dispersion).	Participants watched a 30‐min video lecture on Gauss's law while their eye‐gaze was tracked using a Tobii Pro Spectrum, mind wandering was reported using the self‐caught method and interviewed after the lecture.
Taruffi et al. 2017; Effects of sad and happy music on mind‐wandering and the default mode network	Experiment 1A: 224 participants (137 female, mean age = 33.2, age range 18–55). Experiment 1B: 140 participants (67 female, mean age = 31.4, age range 18–63).	To investigate the influence of sad and happy music on mind wandering and its underlying neuronal mechanisms using thought sampling and fMRI.	Thought sampling probes, meta‐awareness ratings, self‐referential thoughts, fMRI scans, centrality maps of the Default Mode Network (DMN).	Participants listened to sad and happy music while thought probes assessed mind wandering and meta‐awareness, analyzed fMRI data to compare centrality in DMN regions between sad and happy music conditions, examined content of participants' thoughts.
Portnova et al. 2020; The effect of experimental conditions, the sample size and session duration on resting‐state subjective experience	217 native Russian speakers; EEG group: 109 healthy volunteers (61 females, age 23.36 ± 3.2); fMRI group: 108 healthy volunteers (50 females, age 24.95 ± 4.1).	To study the differences in resting‐state subjective experience during EEG and fMRI experimental conditions using the Amsterdam Resting‐State Questionnaire (ARSQ) 2.0.	Amsterdam Resting‐State Questionnaire (ARSQ) 2.0 responses, confirmatory factor analysis, Mann–Whitney *U*‐test.	Participants completed a 10‐min resting‐state session with EEG or fMRI recording, followed by filling out the ARSQ 2.0 questionnaire, data analysis compared differences in subjective experience between EEG and fMRI groups.
Cole and Tubbs 2022; Predictors of obsessive‐compulsive symptomology: mind wandering about the past and future	102 UK‐residing participants aged 19–80, recruited via York St John University, social networks, and local groups.	To investigate the role of spontaneous and future‐oriented mind wandering (MW) in predicting obsessive‐compulsive (OC) symptoms.	Mind wandering: Deliberate and Spontaneous Scale (MW‐D, MW‐S), Dimensional Obsessive‐Compulsive Scale (DOCS), Involuntary Autobiographical Memory Inventory.	Cross‐sectional online questionnaire assessing OCD symptomology, MW frequency, and spontaneous past and future thoughts, data collected in May 2020, analysis of relationships between MW types and OC symptoms.
Kam et al. 2012; Mind wandering and motor control: off‐task thinking disrupts the online adjustment of behavior	Experiment 1: 22 participants, all right‐handed. Experiment 2: 15 participants (nine females; M = 24.8 years, SD = 2.20), all right‐handed.	To determine whether mind wandering episodes can also be considered as periods of “response‐independent” thought with the mind disengaged from adjusting behavioral outputs.	Visuomotor tracking task, time‐estimation task, event‐related potentials (ERPs), P3 ERP component, error‐related negativity (fERN).	Participants performed a motor tracking task (Experiment 1) or time‐estimation task (Experiment 2) while occasionally reporting their attention state, EEG and behavioral data recorded, analysis of tracking error, P3 ERP component, and fERN.
Raymond et al. 2018; Increased frequency of mind wandering in healthy women using oral contraceptives	71 participants (28 women using OC, 14 naturally cycling women, 29 men; aged 18–35)	To examine the frequency and nature of mind wandering in women using oral contraceptives compared to naturally cycling women and men	Frequency and nature of mind wandering (guilt/fear oriented and positive), salivary sex hormone levels	Short version of Imaginal Process Inventory, salivary hormone assessment, Beck Depression Inventory‐II
Benedek et al. 2017; Eye behavior associated with internally versus externally directed cognition	46 young adults (65% female, 33% male, 2% other; aged 18–33; M = 23.3, SD = 4.0)	To investigate potential differences in eye behavior between goal‐directed forms of internally directed cognition (IDC) and externally directed cognition (EDC)	Eye behavior (fixations, pupil diameter, eye vergence, blink duration, microsaccade frequency, angle of eye vergence)	Experimental tasks (anagram and sentence generation) with manipulated focus of attention (internal vs. external)
Qu et al. 2015; The relationship between mind wandering and dangerous driving behavior among Chinese drivers	295 drivers (aged 19–55; balanced in gender and driving years; all had driving licenses and more than 1 year of driving experience	To investigate the relationship between mind wandering during everyday life and dangerous driving behavior	Frequency of mind wandering, risky driving, aggressive driving, negative cognitive/emotional driving, drunk driving, self‐reported traffic accidents, penalty points, fines	Mind Wandering scale (MW), Dula Dangerous Driving Index (DDDI), Demographic questionnaire, surveyed participants around parking lots or residential areas in Beijing
Seli et al. 2016; Intrusive thoughts: linking spontaneous mind wandering and OCD symptomatology	2636 undergraduate psychology students (mean age = 22.49; 1857 females)	To examine how rates of deliberate and spontaneous mind wandering vary with symptoms of obsessive–compulsive disorder (OCD)	Mind Wandering: Spontaneous (MWS) scale, Mind Wandering: Deliberate (MW‐D) scale, Dimensional Obsessive–Compulsive Scale (DOCS)	Questionnaires administered in the first month of classes, randomized order of presentation, completion of every item of each questionnaire, partial course credit awarded
Palagini et al. 2016; Multiple phenotypes of resting‐state cognition are altered in insomnia disorder	47 individuals with insomnia disorder (48.66 ± 15.62 years; 31 women) and 29 healthy controls (50.66 ± 15.14 years; 17 women)	To understand the quantitative nature of thoughts and feelings during mind wandering in insomniacs and healthy controls and their relationship with sleep‐related parameters	Amsterdam Resting‐State Questionnaire (ARSQ), Insomnia Severity Index (ISI), Pittsburgh Sleep Quality Index (PSQI), Dysfunctional Beliefs and Attitudes About Sleep Scale (DBAS)	5‐min eyes‐closed wakeful rest model, ARSQ completed after resting session, ISI, PSQI, DBAS, multiple regression for statistical analysis
Scheutz et al. 2023; Estimating systemic cognitive states from a mixture of physiological and brain signals	82 participants (aged ∼20 years, 46.8% female; recruited from local community; all had driving licenses and drove at least once a week)	To investigate the detection of human cognitive states (e.g., cognitive load, distraction, mind wandering) using physiological and neurophysiological measurements	Physiological parameters (heart rate, respiration rate, blood pressure, skin conductance) and brain activity (fNIRS, EEG)	Multitasking interactive experimental setting, machine learning framework, driving simulator, surveys on driving history and demographics, physiological monitoring equipment, fNIRS, EEG, DRT setup
Sanders et al. 2016; Can I get me out of my head? Exploring strategies for controlling the self‐referential aspects of the mind‐wandering state during reading	96 undergraduate students (42 males; mean age = 20.1, SD = 2.0; range = 18–29 years)	To explore the effects of different instructions on participants' capacity to control mind wandering and maximize reading comprehension	Self‐focus induction (self‐prime vs. other‐prime), reading instructions (external vs. internal), mind wandering measure (NYC–Q), incidental memory for the prime	Counterbalanced design, reading two texts, self‐focus induction using adjectives, mind wandering and comprehension assessed using NYC–Q, reading comprehension questions
Mrazek et al. 2012; Mindfulness and mind‐wandering: finding convergence through opposing constructs	Study 1: 113 undergraduate students (mean age = 19, SD = 1.33); Study 2: 60 undergraduate students (mean age = 19, SD = 1.17)	To clarify the relationship between mindfulness and mind wandering and examine the effects of mindfulness on mind wandering	MAAS (Mindful Attention and Awareness Scale), self‐reported daydreaming, experience sampling of mind wandering, SART (Sustained Attention to Response Task)	Study 1: Correlational design; Study 2: Experimental design with mindful breathing exercise, passive relaxation, and reading
Compton et al. 2023; Effects of task context on EEG correlates of mind‐wandering	59 undergraduates (aged 18–22; 21 men, 33 women, four nonbinary individuals, one undisclosed)	To examine how mind wandering and its neural correlates vary across tasks with different attentional demands	Self‐reported episodes of mind wandering, alpha oscillations, P2 component amplitudes	SART and Stroop selective attention tasks, experience‐sampling probes, EEG recording, counterbalanced task order, retrospective reports
McVay and Kane 2009; Conducting the train of thought: working memory capacity, goal neglect, and mind wandering in an executive‐control task	244 undergraduates (aged 18–35)	To test the relations among working memory capacity (WMC), mind wandering, and goal neglect in a sustained‐attention‐to‐response task (SART)	WMC (operation span, symmetry span, reading span), mind wandering rates, goal neglect errors	SART versions with conceptual vs. perceptual processing demands, thought content probes following no‐go targets, WMC and SART sessions completed during 1 semester, 90 min WMC screening sessions
Ottaviani et al. 2013a; Flexibility as the key for somatic health: from mind wandering to perseverative cognition	73 healthy participants (31 men, mean age 25.2 years; 42 women, mean age 23.4 years)	To compare the cardiac and cognitive correlates of perseverative cognition (PC) and mind wandering (MW)	Cognitive flexibility (reaction times, intrusiveness, efforts to inhibit), autonomic rigidity (heart rate variability), mood	Recall interviews on neutral and personally relevant negative episodes, 20‐min tracking task with thought probe, ECG recording, personality questionnaires, mood ratings
Hutchison et al. 2020; Measuring task set preparation versus mind wandering using pupillometry	118 Montana State University undergraduate students (typically freshmen, aged 18–20, approximately 55%–60% female)	To investigate participants’ task set preparation by measuring changes in pupil diameter and using thought probes to gauge “on‐task” thoughts vs. mind wandering	Pupil diameter changes, self‐reported mind wandering	Four fixation delays (500, 2000, 4000, 8000 ms), two saccade trial types (prosaccade and antisaccade), thought probes, pupillometry during 4000 and 8000 ms conditions
Smallwood et al. 2014; Subjective experience and the attentional lapse: task engagement and disengagement during sustained attention	Experiment 1: 21 participants (mean age = 27.8 years); Experiment 2: 12 participants (mean age = 21.25 years); Experiment 3: 41 participants (mean age = 24.8 years)	To investigate the relationship between subjective experience and attentional lapses during sustained attention	Thought probes, questionnaires (CES‐D, RSQ, DSSQ, CFQ), reaction time, errors, GSR, HR	Sustained Attention to Response Task (SART), thought probes, galvanic skin response (GSR), heart rate (HR), questionnaires, different target durations (short and long)
Prieto et al. 2021; Local oscillatory brain dynamics of mind wandering in schizophrenia	22 adults from the Mental Health Unit of the San Agustín University Hospital in Spain (aged 23–53; seven females; all right‐handed; diagnosed with schizophrenia)	To explore EEG local synchrony of mind wandering (MW) associated with schizophrenia compared to task‐focused states	Power of EEG oscillations in different frequency bands, cognitive variables (processing speed, working memory), psychopathology (PANSS)	Participants watched 4 video clips (5 min each), visual and auditory information matching or not, cognitive evaluation using SCIP‐S, EEG recording, PANSS, chlorpromazine equivalents for antipsychotic dosage
Smallwood et al. 2015; Representing representation: integration between the temporal lobe and the posterior cingulate influences the content and form of spontaneous thought	None specified | 87 participants (recruited from the Department of Psychology at the University of York; age range 18–31)	To examine how interactions between the nodes of the default mode network (DMN) give rise to particular mental experiences during spontaneous thought	Seed‐based functional connectivity, contents of spontaneous thought	Neurocognitive function during resting state measured on Day 1, subjective experience assessed in a subsequent laboratory session on Day 2, online experience sampling
Arnau et al. 2019; Inter‐trial alpha power indicates mind wandering	33 participants (aged 18–60, mean age = 29.79 years, 20 females) who reported mind wandering at least 30 times during the task	To clarify the temporal dynamics of mind wandering using EEG and a cluster‐based permutation approach	Alpha power during intertrial intervals, response accuracy	Switching task with digits presented at central position, mind wandering assessed via thought probes after trial completion, EEG recording with 32 electrodes
Smallwood and O'Connor 2011; Mind‐wandering and dysphoria	37 participants (11 males, 26 females; mean age = 25.6 years)	To examine whether mind wandering provides a useful marker of cognition in dysphoria during a word learning task	Mind wandering accessibility, response times, physiological arousal (heart rate), skin conductance, autobiographical memory retrieval latency	Word learning task, physiological measures (SCR, heart rate), three questionnaires (CES‐D, RSQ, DSSQ), data collection on encoding and retrieval performance during periods of mind wandering
Golchert et al. 2017; Individual variation in intentionality in the mind‐wandering state is reflected in the integration of the default‐mode, fronto‐parietal, and limbic networks	123 healthy volunteers (age: M = 26.59, SD = 4.23; 59 females)	To examine the cortical organization underlying individual differences in the intentionality of mind wandering	Cortical thickness, functional connectivity, mind wandering scales (MW‐D, MW‐S)	Multimodal MRI analysis, four‐item Mind wandering: Deliberate (MW‐D) scale, four‐item Mind‐wandering: Spontaneous (MW‐S) scale, resting‐state fMRI scans, high‐resolution structural image
Robison et al. 2017; Cognitive and contextual correlates of spontaneous and deliberate mind‐wandering	210 participants (University of Oregon human subjects pool)	To examine whether elevated rates of mind wandering among low‐ability individuals are due to deliberate, intentional episodes or spontaneous, unintentional failures to maintain task‐oriented attention	Working memory capacity (operation span, symmetry span, reading span), mind wandering (thought probes), contextual variables (motivation, alertness, task unpleasantness)	Participants completed all measures in a single 2‐h session, three attention control tasks (psychomotor vigilance, antisaccade, Stroop), thought probes, working memory tasks
Krimsky et al. 2018; The influence of time on task on mind wandering and visual working memory	143 undergraduate students (females = 88, Mage = 19.09, SDage = 1.37) recruited from the University of Miami psychology subject pool.	To investigate the consequences of mnemonic demand, mind wandering, and time on task during a visual working memory task.	Working memory task performance, self‐reported mind wandering (Likert‐type scale: 1 = on‐task, 6 = off‐task)	Delayed‐recognition visual working memory task, mnemonic load manipulation (1 item = low load; 2 items = high load), self‐report Likert‐type scale for mind wandering, hierarchical linear modeling
Faber et al. 2018; An automated behavioral measure of mind wandering during computerized reading	132 college students (90 from a private Midwestern US university, 42 from a public university in the Southern United States; average age 20.3 years, 62% female)	To develop an eye‐gaze‐based, machine‐learned model of mind wandering during computerized reading	Eye‐gaze data, self‐reported mind wandering, text comprehension, trait‐based mind wandering questionnaire, retrospective engagement/attention questionnaire	Participants read an excerpt from a book on a computer screen, eye‐gaze data recorded using Tobii TX300 or T60 eyetracker, machine learning classification models trained to discriminate between mind wandering and normal reading
Wong et al. 2023; Commonalities between mind wandering and task‐set switching: an event‐related potential study	16 participants (13 females, three males; age: mean = 23.00 years, SD = 4.05 years, range = 18–32 years) who had at least 10 correct trials per condition	To test if mind wandering during task‐set switching promotes switching‐related mental processes using event‐related potentials (ERPs).	P300 amplitude, response times, self‐reported mind wandering	Task‐switching paradigm, electroencephalography (EEG), thought probes at the end of each block, event‐related potentials (ERPs), Mind Wandering: Deliberate (MW‐D) and Spontaneous (MW‐S) scales
Garrison et al. 2013; Real‐time fMRI links subjective experience with brain activity during focused attention	Experiment 1A: 22 meditators and 22 nonmeditators; Experiment 1B: nine meditators and 11 nonmeditators; Experiment 2: 10 meditators (nine right‐handed, one ambidextrous)	To link objective measures of brain activity with reports of subjective experience using real‐time fMRI during a focused attention task	Real‐time fMRI feedback, subjective experience reports	Participants completed a focused attention task with real‐time fMRI feedback from the posterior cingulate cortex, self‐reported subjective experience, Experiment 1A and 1B involved real‐time feedback tasks, Experiment 2 involved a blinded discovery fMRI protocol
Zanesco et al. 2020; Quantifying streams of thought during cognitive task performance using sequence analysis	545 undergraduates from the University of North Carolina at Greensboro (UNCG)	To apply sequence analytic methods to quantify the dynamics of thought from time series sequences of categorical experience sampling thought probes over time.	Categorical thought probe sequences, entropy, complexity, turbulence, Markov chain transition probabilities	Experience sampling of thought content during five different cognitive tasks (SART, Arrow Flanker, Letter Flanker, Number Stroop, 2‐Back), sequence analysis using package TraMiner, Markov chain transition analysis using package seqHMM
Young et al. 2021; Contrasting electroencephalography‐derived entropy and neural oscillations with highly skilled meditators	28 participants (seven shamatha, six zazen, six vipassana, five dzogchen, three visualization, and one tonglen practitioner)	To identify the electrophysiological correlates of different meditation practices compared with mind wandering	EEG‐derived entropy (Lempel–Ziv complexity), power spectra at six frequency bands	EEG activity recorded with 16 channel Ultracortex Mark IV according to the International 10–20 System, participants engaged in both meditation and mind wandering tasks, initial EEG baseline collected
Aitken et al. 2023; Task‐related and task‐unrelated thoughts in runners and equestrians: measurement issues in evaluations of thought content	74 trail runners (34 men, 39 women, one unreported; 80.7% between 31 and 55 years old) and 23 equestrians (average age 36–40, all female)	To explore the relationship between self‐reported thought content and performance criteria in runners and equestrians, examining method issues.	Task‐related thoughts, task‐unrelated thoughts	Cross‐sectional survey of trail runners and equestrians, measures of task‐related and task‐unrelated thoughts using items from the Short Task Relevant Evaluation of Stress State (STRESS), self‐reported thought content
Kane et al. 2021; Testing the construct validity of competing measurement approaches to probed mind‐wandering reports	1108 students (760 UNCG and 348 WCU) from 2015 to 2018	To explore the construct validity of probed mind wandering reports using different thought‐probe types across cognitive tasks	Four probe types (content, intentionality, depth, content with performance‐evaluative thoughts), TUT rates, TUT‐report confidence ratings, associations with consciousness‐related constructs	Laboratory data from over 1000 undergraduates at two US institutions, cognitive tasks (ANTIS‐LET, SART, FLANKER, ANTI‐ARO), self‐report questionnaires (AD/HD Rating Scale IV, CFQ‐MAL, CAS, IPI, NEO‐FFI‐3, SAQ, WBSI, ARS), thought probes in both SART and flanker tasks
Prieto‐Alcántara et al. 2023; Alpha and gamma EEG coherence during on‐task and mind wandering states in schizophrenia	Group of schizophrenia patients and a group of healthy controls from a larger investigation (Iglesias‐Parro et al. 2020)	To apply a task‐related EEG coherence approach to understand cognitive processing in patients with schizophrenia and healthy controls	EEG coherence in alpha and gamma frequency bands	EEG coherence analyzed during the performance of an ecological task of sustained attention, comparison of on‐task and mind wandering states, cognitive functioning measured using SCIP‐S, psychopathology assessed using PANSS
Polychroni et al. 2023; Response time fluctuations in the sustained attention to response task predict performance accuracy and meta‐awareness of attentional states	74 healthy controls (48 females, age range: 18–48, MAge = 24.5, SD = 7.1; years of education (postsecondary school): MYoe = 3.6, SD = 2.3)	To investigate the relationship between response time (RT) patterns in the Sustained Attention to Response Task (SART) and meta‐awareness of attentional states	RT patterns, target performance, self‐reported attentional state, meta‐awareness	Principal component analysis of RT patterns in nontarget (go) trials prior to target (no‐go) trials, attentional state and meta‐awareness probes, comparison of RT patterns and performance, implications for introspective methods and measurement of mind wandering
Yeung and Fernandes 2024; Recurrent involuntary memories and mind wandering are related but distinct	2701 undergraduate students from the University of Waterloo (73% women; mean age = 20.2 years, SD = 3.0; range = 17–48)	To investigate the extent to which recurrent involuntary autobiographical memories (IAMs) and mind wandering (MW) converge vs. diverge and their relationships with symptoms of mental health disorders.	Recurrent Memory Scale (Yeung and Fernandes 2020), Deliberate (MW‐D) and Spontaneous (MW‐S) Mind Wandering Scales, PTSD Checklist for DSM‐5, Depression, Anxiety, and Stress Scales (DASS‐21), State‐Trait Inventory of Cognitive and Somatic Anxiety—Trait Version (STICSA‐T)	Self‐report measures of recurrent IAMs, trait MW, and psychopathology (i.e., PTSD, depression, anxiety), data collected in two waves (Winter/January 2020 and Spring/May 2020), regression analysis to assess relationships between IAMs, MW, and disorder symptoms
Leung et al. 2024; Force modulation: a behavioural marker of mind‐wandering	220 University of Waterloo students (67 males, 151 females, two undisclosed; ages 16–30, M = 20.05, SD = 2.21)	To design and test a new mind wandering measure, called the In Sync Task (IST), that can differentiate between spontaneous and deliberate mind wandering more readily than the Metronome Response Task (MRT)	Variabilities in rhythmic response times (RRTs), consistency in modulating clicking force	Participants were randomized to complete either the MRT or the IST, thought probes emerged pseudo‐randomly, behavioral measures tracked using PsychoPy and PowerLab Psychophysiology Data Acquisition System, data analyzed using LabChart 7.2 software
Shin et al. 2015; Media multitasking is linked to attentional errors, mind wandering, and automatised response to stimuli without full conscious processing	238 participants (163 females, Mean age = 35.03, SD age = 11.70, Range 17–67 years)	To examine the relationships between media multitasking, sustained attention, and inhibitory control using a three‐state attentional disengagement model	Media use questionnaire, Barratt Impulsivity Scale (BIS), Boredom Proneness Scale (BPS), Sustained Attention to Response Task (SART)	Participants completed a media use questionnaire, SART with mind wandering probes, Barratt Impulsivity Scale, and Boredom Proneness Scale; data analyzed for relationships between media multitasking and attentional errors, mind wandering, and inhibitory control
Xu et al. 2019; Attenuation of deep semantic processing during mind wandering: an ERP study	29 participants (15 males, 14 females; M = 24.03 years, SD = 4.46) recruited from the Columbia University community	To investigate the effect of mind wandering on deep (semantic) processing by recording event‐related potentials (ERPs) during a task	Perceptual processing (P2 component), deep processing (late, sustained slow wave)	Participants studied English‐Spanish word pairs while intermittently probed for mind wandering, ERP recording, analysis of perceptual and deep processing during on‐task and mind wandering states
Jubera‐García et al. 2021; Influence of content and intensity of thought on behavioral and pupil changes during active mind wandering, off focus and on‐task states	29 participants (mean age: 21 years; 21 females) with normal vision	To disentangle the content of thought (on‐task or MW) from an off‐focus state of mind by probing the intensity of participants' attention	Behavioral performance, pupil dilation, content of thought (on‐task or MW), intensity of attention	Participants completed a Sustained Attention to Response Task (SART), both behavior and pupil size were measured, the off‐focus state was operationalized by probing the intensity of participants' attention
Martinon et al. 2022; Frogs’ legs versus roast beef: how culture can influence mind‐wandering episodes across the lifespan	308 adults over 18 years of age, both in France and the United Kingdom	To investigate the joint effects of culture and age on mind wandering frequency, mindfulness, mood, rumination, self‐reflection, future thinking, depressive symptoms, and cognitive failures.	Daydreaming Frequency Scale (DDFS), Mindful Attention Awareness Scale (MAAS), Positive and Negative Affect Schedule (PANAS), Future‐Self Thoughts questionnaire (FST), Rumination and Reflection Questionnaire (RRQ)	Large‐scale online questionnaire‐based survey, confirmatory factor analyses to explain mind wandering frequency in French and British populations, self‐report measures of various psychological constructs
Poerio et al. 2017; The role of the default mode network in component processes underlying the wandering mind	157 participants (60% female, Mage = 20.43, SD = 2.63; range = 18–31)	To examine the relationships between individual differences in resting‐state DMN connectivity, performance on memory, social and planning tasks, and variability in spontaneous thought.	Resting‐state DMN connectivity, memory tasks, social cognition tasks, planning tasks, variability in spontaneous thought	Resting state MRI acquisition, 3 days of testing with descriptions of naturally occurring thoughts during a simple nondemanding cognitive task, subsequent task battery, several online questionnaires
Kam et al. 2013; Mind wandering and the adaptive control of attentional resources	Experiment 1: 32 participants (17 women, M = 20.83 years, SD = 1.42 years); Experiment 2: 20 participants (13 women, seven men, M = 24.6 years, SD = 7.1 years)	To investigate the specific capacities retained during mind wandering states that allow adaptive responses to the external environment.	Volitional and automatic visual–spatial attentional orienting, ERPs (auditory N1 component), task‐related attention	Experiment 1: Traditional performance measures, volitional and automatic spatial orienting tasks, attention state reports. Experiment 2: ERPs during auditory task, sensitivity to deviant or unexpected sensory events, mind wandering probes.
Cotton et al. 2023; The effects of mind‐wandering, cognitive load, and task engagement on working memory performance in remote online experiments	Experiment 1: 185 participants (132 online, Mage = 19.7, 80% female; 53 in‐person, Mage = 19.8, 51% female); Experiment 2: 191 online participants (Mage = 19.9, 67% female)	To investigate the effects of mind wandering, cognitive load, and task engagement on working memory performance in remote and in‐person environments.	Mind wandering rates, working memory performance, task engagement, secondary task performance	Participants completed a working memory task with varied cognitive load during a secondary task, reported their mind wandering during each trial, procedures conducted either online or in‐person, analyses of performance, task engagement, and cognitive load
Somaraju et al. 2023; Are mindfulness and mind‐wandering opposite constructs? It depends on how mindfulness is conceptualised	552 participants (60% females, 40% males; ages 18–94, M = 54.06 years, SD = 15.87) recruited by Qualtrics from the United States, India, and Australia	To investigate if trait mindfulness and its components (mindful attention, acceptance, and nonjudging) correlate negatively with self‐reported and indirect markers of mind wandering	Trait mindfulness scales, trait mind wandering scales, Sustained Attention to Response Task (SART)	Participants completed an anonymous online questionnaire and the computer‐based Sustained Attention to Response Task (SART), data analyzed for correlations between mindfulness components and mind wandering
Di Gruttola et al. 2014; Revisiting the association between hypnotisability and blink rate	41 students of Pisa University with high, medium, and low hypnotizability scores	To investigate the association between hypnotizability and blink rate (BR) as an index of dopaminergic tone, while controlling for mind wandering (MW)	Blink rate (BR), relaxation (skin conductance), anxiety, proneness to absorption, mind wandering (MW)	Participants completed questionnaires evaluating anxiety, absorption, and mind wandering before the recording session, BR measured in resting conditions, data analyzed for BR differences among hypnotizability groups, controlling for MW
Mrazek et al. 2011; Threatened to distraction: mind‐wandering as a consequence of stereotype threat	Study 1: 43 female undergraduate students from the University of California Santa Barbara (mean age = 19.0, SD = 1.85); Study 2: 72 female undergraduate students (mean age = 18.76, SD = 0.99)	To test the hypothesis that the threat of a negative stereotype increases the frequency of mind wandering, thereby leading to performance impairments.	Mind wandering frequency, Sustained Attention to Response Task (SART), thought sampling during a math test	Study 1: Between‐subjects design comparing mind wandering frequency among women under stereotype threat and control conditions, stereotype threat induced by diagnostic math test context. Study 2: Stereotype threat manipulated similarly, participants took a math test with thought probes at unpredictable intervals, tested mind wandering frequency and performance.
Meier 2021; Testing the attention‐distractibility trait	235 subjects from Western Carolina University (65% female, mean age = 19 years)	To test whether associations between task‐irrelevant distraction and mind wandering, ADHD symptomology, and working memory capacity can be distinguished from associations with task‐relevant distraction.	Task‐irrelevant distraction, working memory capacity, task‐relevant distraction (antisaccade accuracy), mind wandering (questionnaire and in‐task thought probes), ADHD symptomology	daydreaming questionnaire, operation span, antisaccade, ADHD questionnaire, SART, symmetry span, and demographic questionnaire. Experimenters read instructions aloud, white noise machine used, tasks programmed with E‐Prime software.
Pereira et al. 2023; Trait‐level variability in attention modulates mind wandering and academic achievement	Experiment 1: 128 participants recruited via Amazon Mechanical Turk, 97 completed the study (57M, 40F, Mage = 35 years, SDage = 11 years)	To study the role of intrinsic temperament traits in moderating the association between mind wandering and academic success.	Mind Wandering Questionnaire, Adult Temperament Questionnaire, academic grades, visual metronome response task (objective measure of mind wandering)	Experiment 1: Participants completed the Mind Wandering Questionnaire, Adult Temperament Questionnaire, and reported their academic grades. Experiment 2: Used visual metronome response task to confirm links between effortful control and mind wandering, analysis of academic success
Gionet et al. 2011; Psychopathology and mind wandering in young university students	60 university students (65% female; aged 17–32 years, M = 21.67 years, SD = 3.08 years)	To document the associations between cognitive disengagement syndrome (CDS), ADHD symptoms, and mind wandering while controlling for age, sex, internalized symptoms, and sleep.	Mind Wandering Questionnaire (MWQ), thought sampling during a reading task, measures of CDS, ADHD symptoms, internalized functioning, and insomnia	Participants completed measures of CDS, ADHD symptoms, internalized functioning, and insomnia, mind wandering assessed using MWQ and thought sampling during a reading task, multiple regression analyses conducted to identify predictors of mind wandering
Ralph et al. 2017; Wandering minds and wavering goals: examining the relation between mind wandering and grit in everyday life and the classroom	Study 1: 100 undergraduate students (68 female); Study 2: more heterogeneous sample; Study 3: university students during lectures	To examine the relation between mind wandering and the personality trait of “grit,” and how it affects long‐term goals that require sustained interest and effort.	Grit Scale (Duckworth et al. 2007), Spontaneous and Deliberate Mind Wandering Scales (MW‐S and MW‐D), Mindful Attention Awareness Scale—Lapses Only (MAAS‐LO), Attention‐Related Cognitive Errors Scale (ARCES), Attentional Control: Switching (AC‐S) and Distractibility (AC‐D), Media Multitasking Index (MMI)	Study 1: Online questionnaires to examine relation between mind wandering and grit in everyday life. Study 2: Replication with more heterogeneous sample, measure of conscientiousness, and general perseverance. Study 3: Relation between mind wandering and grit in the classroom during university lectures.
Uzzaman and Joordens 2011; The eyes know what you are thinking: eye movements as an objective measure of mind wandering	33 participants	To explore the use of eye movements as an objective measure of mind wandering during a reading task.	Eye movements (fixation, saccadic duration and count, within‐word regressions), self‐reported mind wandering episodes	Participants performed a reading task while their eye movements were recorded. They were probed every 2–3 min to indicate whether their mind was wandering. Eye movements were tracked using an EyeLink 1000 tracker.
Faber et al. 2020; The eye–mind wandering link: identifying gaze indices of mind wandering across tasks	132 college‐aged adults (age: M = 19.8 years, SD = 1.51 years; 74% female	To identify gaze indices of mind wandering across tasks with varying visual demands and understand how the visual system prioritizes external information during mind wandering.	Eye movements (fixations, saccades, dispersion), mind wandering (via thought‐probes)	Participants completed a battery of seven short tasks with different visual demands while their eye movements were recorded using Tobii EyeX and Eyelink 2k eye tracking systems, mind wandering assessed via thought‐probes, data analyzed for gaze behaviors during mind wandering.
Seli et al. 2014; Restless mind, restless body	74 participants enrolled in psychology courses at the University of Waterloo	To investigate the hypothesis that failures of task‐related executive control during episodes of mind wandering are associated with an increase in extraneous movements (fidgeting).	Thought probes to assess mind wandering, metronome response task (MRT), Wii Balance Board to measure fidgeting, self‐report measure of fidgeting (SAQ)	Participants performed the MRT while sitting on a Wii Balance Board, mind wandering assessed using thought probes, movement data collected from Wii Balance Board, data analyzed for fidgeting and response variability during mind wandering and on‐task periods
Nakatani et al. 2023; Prior EEG marks focused and mind‐wandering mental states across trials	Participants performed a task of counting tones (between 20 and 24) while their EEG was recorded.	To investigate neural substrates of mental dynamics during transitions between task‐focused and mind wandering states.	EEG alpha‐band activity (DMN), auditory evoked potentials (AEP, CCN), task performance, self‐reported number of thoughts	Participants reported mind wandering and task‐focused states during a tone counting task, EEG recorded continuously, measures of DMN and CCN activity computed, effects on task performance and number of thoughts estimated.
Zanesco et al. 2021; Associations between self‐reported spontaneous thought and temporal sequences of EEG microstates	61 healthy volunteers aged 18–35 recruited by Portnova et al. (2019)	To examine associations between the intrinsic dynamics of EEG microstates and self‐reported thought measured using the Amsterdam Resting‐State Questionnaire (ARSQ).	EEG microstates, Amsterdam Resting‐State Questionnaire (ARSQ)	Participants completed paper‐based questionnaires, EEG recorded while resting, self‐reported thought using ARSQ, hierarchical clustering of ARSQ ratings, sequence analysis of EEG microstates, reliability evaluated using meta‐analysis.
Long et al. 2024; How mind wandering influences motor control: the modulating role of movement difficulty	Experiment 1: 30 university students (19 women; M = 24.07 years, SD = 2.42 years); Experiment 2: 29 university students (18 women; M = 22.79 years, SD = 2.20 years)	To investigate whether the impact of mind wandering on motor control is modulated by movement difficulty and its associated neural mechanisms.	Reaction time, contralateral delta‐theta functional connectivity, midfrontal delta‐theta activity, self‐reported mind wandering	Participants performed key‐pressing and key‐releasing movements with specified fingers in signal‐response tasks, intermittently reported attention as “On task” or “Off task,” neural measures recorded using EEG, data analyzed for reaction time, connectivity, and activity during different movement difficulties.
Hoffmann et al. 2016; Where the depressed mind wanders: self‐generated thought patterns as assessed through experience sampling as a state marker of depression	25 MDD patients and 26 matched healthy controls recruited from Charité‐Universitätsmedizin Berlin	To investigate the specific contents of self‐generated thoughts (SGTs) in individuals with Major Depressive Disorder (MDD) and how they vary over time.	Self‐generated thoughts (off‐task, positive/negative, self/other‐related, past/future‐oriented), Beck Depression Inventory (BDI), Hamilton Depression Rating Scale (HAMD‐17)	Participants performed a mind wandering task involving nondemanding number discriminations, intermittent probe questions assessed current SGTs, multilevel modeling used to analyze SGT patterns, thoughts assessed on six dimensions.
Fix 2017; Examining how regular meditation practice influences the neural oscillatory activity associated with refocusing attention after a mind wandering episode	Regular meditators recruited from a medium‐sized Mid‐Atlantic university and local meditation groups; meditation‐naive participants recruited from introductory psychology courses	To examine the effects of meditation on the interactions between default mode network (DMN), fronto‐parietal control network (FPCN), and dorsal attention network (DAN) during mind wandering.	EEG activity (event‐related spectral perturbations, ERSP), self‐reported incidences of mind wandering, connectivity between network nodes	Between‐groups design comparing novice and regular meditators, EEG activity and self‐reports of mind wandering recorded, independent component analysis to identify network nodes, functional connectivity analysis conducted, surveys included Five Facet Mindfulness Questionnaire and Perceived Stress Scale
Wang et al. 2018; Patterns of thought: population variation in the associations between large‐scale network organisation and self‐reported experiences at rest	258 participants (females = 162; age range 18 – 55, M = 34.97, SD = 12.24) obtained from the enhanced Nathan Kline Institute‐Rockland sample (NKI‐RS)	To examine whether dimensions of population variation in different modes of unconstrained processing can be described by the associations between patterns of neural activity and self‐reports of experience during the same period.	Resting‐state functional magnetic resonance imaging (fMRI), New York Cognition Questionnaire (NYC‐Q), Delis‐Kaplan Executive Function System (D‐KEFS), Wechsler Abbreviated Scale of Intelligence (WASI‐II), Wechsler Individual Achievement Test—Second Edition Abbreviated (WIAT‐IIA)	Participants' resting‐state fMRI data and self‐reports of experience during the scan were analyzed, machine learning used to determine patterns of association between neural and self‐reported data, four dimensions of experiences identified, cognitive function assessed in a separate laboratory session.
Konishi 2017; Window into the wandering mind: investigating the neural and pupillometric correlates of mind wandering with a dual task paradigm	Behavioral study: 29 healthy participants (nine males, age = 21.7 ± 2 years); Task‐based fMRI study: 20 healthy, right‐handed participants (nine males, age = 23.8 ± 3 years)	To develop a novel paradigm for the study of mind wandering and investigate the potential of DMN activity and baseline pupil size as markers of mind wandering.	Default mode network (DMN) activity, baseline pupil size, self‐reported incidences of mind wandering, event‐related spectral perturbations (ERSP), connectivity between network nodes	Between‐groups design comparing novice and regular meditators, EEG activity and self‐reports of mind wandering recorded, independent component analysis to identify network nodes, functional connectivity analysis conducted, dual‐task paradigm with 0—back and 1—back conditions
Yamaoka and Yukawa 2020; Mind wandering in creative problem‐solving: relationships with divergent thinking and mental health	865 participants (458 men, 390 women, 17 unknown; Mage = 18.99 years, SD = 1.16) from University of Tsukuba	To examine the relations among mind wandering, divergent thinking, and mental health while controlling for each of their confounding effects.	Mind wandering traits, divergent thinking (Unusual Uses Test, UUT), mental health measures (depressive symptoms, schizotypal personality, CES‐D)	Participants completed a questionnaire measuring mind wandering traits, divergent thinking, and mental health measures. Divergent thinking assessed using UUT, depressive symptoms measured using CES‐D, multiple regression analysis conducted to identify relationships.
Morava et al. 2024; Acute stress negatively impacts on‐task behavior and lecture comprehension	40 participants (20 female, mean age = 22.3; SD = 2.6, age range = 18–28) from Western University	To examine the effects of acute stress on mind wandering during a lecture and subsequent lecture comprehension in young adults.	Heart rate, blood pressure, salivary cortisol, state anxiety (STAI), mind wandering assessments (MW1, MW2, MW3), lecture comprehension assessment	Participants randomized to acute stress induction via Trier Social Stress Test or rest before watching a 20‐min video lecture with embedded mind wandering probes, stress responses and mind wandering assessed during the lecture, lecture
				comprehension assessed afterward
Unsworth et al. 2019; Individual differences in baseline oculometrics: examining variation in baseline pupil diameter, spontaneous eye blink rate, and fixation stability	204 participants (66.5% female, ages 18–27, M = 19.09, SD = 1.75) from the University of Oregon	To examine individual differences in baseline oculometrics (baseline pupil diameter, spontaneous eye blink rate, fixation stability) and their relation with cognitive abilities, personality traits, and self‐report assessments.	Baseline pupil diameter, spontaneous eye blink rate, fixation stability, working memory capacity, attention control, off‐task thinking, personality (Big Five Inventory), ADHD symptomology, morningness–eveningness, mind wandering	Participants completed a baseline eye measure, followed by a questionnaire about their thoughts during the measure. Various cognitive ability measures, attention control tasks, and self‐report questionnaires were administered, data analyzed for correlations between baseline oculometrics and individual differences constructs.
Seli et al. 2017; What did you have in mind? Examining the content of intentional and unintentional types of mind wandering	150 undergraduate students from the University of Waterloo, data analyzed from 146 participants	To determine whether intentional and unintentional types of mind wandering differ in content.	Choice Reaction Time task (CRT), thought probes, self‐reported mind wandering content	Participants completed a CRT task, categorized their mind wandering as intentional or unintentional, and responded to questions about the content of their mind wandering. Data analyzed for differences in content ratings between intentional and unintentional mind wandering, paired‐samples *t*‐tests and repeated‐measures ANOVA conducted.
Zanesco et al. 2023; Experience sampling of the degree of mind wandering distinguishes hidden attentional states	537 United States military service members (predominantly male, 24 female; average age = 28.59, SD = 8.079, range = 18–54)	To examine distinct patterns in subjective reports of task‐related attentional focus during a sustained attention task.	Experience sampling probes, task‐related focus ratings (1 = on‐task to 6 = off‐task), behavioral performance, Markov‐chain modeling of probe ratings	| Participants completed the Sustained Attention to Response Task (SART) with embedded experience sampling probes, assessing task‐related focus using a continuum of response ratings. Behavioral performance preceding probes was analyzed, Markov‐chain modeling conducted to reveal hidden states underlying probe rating behavior, findings replicated in two additional independent datasets.
Martarelli and Ovalle‐Fresa 2024; In sight, out of mind? Disengagement at encoding gradually reduces recall of location	Study 1: 54 participants; Study 2: 104 participants (22 participants with enough off‐task trials for standard mixture model)	To investigate the impact of task disengagement at encoding on subsequent recall of location using a continuous delayed estimation paradigm.	Thought probes (dichotomous and continuous response scale), recall of location measured in degrees, standard mixture model	Participants performed a memory task with thought probes assessing task disengagement at encoding, recall of location measured in degrees, dichotomous and continuous response scales used, two studies conducted, analysis of relationship between task disengagement and recall of location, findings replicated in the second study.
Laursen et al. 2023; Examining the effect of expected test format and test difficulty on the frequency and mnemonic costs of mind wandering	Experiment 1: 249 participants (155 from University of Guelph, 94 from Prolific); Experiment 2: 168 participants from Prolific	To understand how the circumstances surrounding a learning episode affect the frequency of mind wandering and its impact on memory performance across different test formats.	Mind wandering rates, memory performance (cued‐recall and forced‐choice recognition tasks), participants' confidence in learning, media multitasking	Participants completed experiments using PsychoPy software, accessible via Pavlovia, with weakly related cue‐target word pairs. Experiment 1 included a cued‐recall task; Experiment 2 varied the difficulty of the forced‐choice recognition task, participants' confidence in learning, changes in confidence, actual memory performance, and media multitasking assessed, data analyzed for mind wandering rates and memory performance costs.
Kuehner et al. 2017; Lab meets real life: a laboratory assessment of spontaneous thought and its ecological validity	43 university students (26 women, 17 men; Mage = 21.74 years, SD = 3.14) from the University of Mannheim	To examine the stability of spontaneous thought (ST) dimensions assessed in the lab and their predictive value with respect to mind wandering (MW), repetitive negative thought (RUM), and affect in daily life.	Amsterdam Resting State Questionnaire (ARSQ 2.0), mind wandering and rumination intensity, positive and negative affect, baseline questionnaires (CES‐D, STAI‐T, RSQ_10D, Mindful Attention and Awareness Scale)	Participants assessed with ARSQ 2.0 during two lab sessions, separated by 5 days of electronic ambulatory assessment (AA) using a smartphone. Participants indicated intensity of MW, RUM, and mood in daily life ten times a day during AA. Hierarchical linear models and mediation analyses conducted to examine relationships between ST dimensions and cognitive and affective states.
Wammes et al. 2016; Mind wandering during lectures I: changes in rates across an entire semester	154 undergraduate students (97 female, 57 male, ages 16–38, M = 20.117, SD = 2.092) from the University of Waterloo	To examine rates of students' mind wandering throughout a 12‐week undergraduate course, and how these rates change over time within an average lecture, an average week, and the term.	Thought probes assessing intentional and unintentional mind wandering rates, individual observations of mind wandering, student demographics	Thought probes placed intermittently within lectures, mind wandering rates assessed on a large scale, analysis of changes over time within lectures, weeks, and the term, statistical analyses conducted to evaluate mind wandering patterns.
Konishi et al. 2017; When attention wanders: pupillometric signatures of fluctuations in external attention	Experiment 1: 42 participants (18–28 years, mean age 19.4; eight males), 32 participants analyzed; Experiment 2: 42 participants (18–39 years, mean age 21.5; 11 males), 36 participants analyzed	To understand the extent to which states of internal and external attention can be determined using pupillometry as an index of ongoing cognition.	Baseline pupil size, thought probes, response accuracy, reaction times	Participants completed a task featuring 0—back and 1—back conditions using PsychoPy2. Pupillometry used to measure baseline pupil size, thought probes assessed internal and external attention states, response accuracy and reaction times recorded. Data analyzed for associations between pupil size, attention states, and task performance.
O'Neill et al. 2020; Dissociating the freely‐moving thought dimension of mind‐wandering from the intentionality and task‐unrelated thought dimensions	120 participants recruited via Amazon Mechanical Turk (age range: 25–30)	To evaluate how the task‐relatedness, intentionality, and constraint dimensions of thought during mind wandering relate to each other.	Clock task performance, thought probes assessing task‐relatedness, intentionality, and thought constraint	Participants completed a clock task with intermittent thought probes, mind wandering indexed using probes assessing task‐relatedness, intentionality, and constraint dimensions. Data analyzed for associations between these dimensions.
Cheyne et al. 2008; Anatomy of an error: a bidirectional state model of task engagement/disengagement and attention‐related errors	504 participants (349 females, 155 males; mean age = 32.23, SD = 11.23) selected from prior respondents to a WWW survey on sleep paralysis	To present a three‐state attentional model of task engagement/disengagement and analyze it using the Sustained Attention to Response Task (SART).	Reaction time (RT) variability, anticipations, omissions, self‐reported mind wandering, everyday cognitive errors	Participants completed 225 trials of the SART, responses analyzed for RT variability, anticipations, and omissions. Self‐report measures included ARCES and MAAS‐LO. Regression and lag‐sequential analyses conducted to test model predictions and examine temporal associations between state indicators and SART errors.
Seli et al. 2016; On the necessity of distinguishing between unintentional and intentional mind wandering	113 undergraduate students	To provide evidence that intentional and unintentional types of mind wandering are qualitatively different and should be separately assessed.	Standard Sustained Attention to Response Task (SART), sequential SART, thought probes assessing intentional and unintentional mind wandering	Participants completed difficult and easy versions of the SART, thought probes used to assess mind wandering types, participants categorized their mental state as on task, intentionally mind wandering, or unintentionally mind wandering. Data analyzed for differences between intentional and unintentional mind wandering.
Drescher et al. 2016; Absence without leave or leave without absence: examining the interrelations among mind wandering, metacognition and cognitive control	63 participants (50 female, aged between 17 and 24 years, Mage = 18.7 years, SD = 1.5) from Vrije Universiteit Brussel	To examine the interrelations among mind wandering, metacognition, and cognitive control.	Mind wandering (Sustained Attention to Response Task, SART), metacognitive efficiency (perceptual decision task with confidence ratings), cognitive control (conflict task)	Participants completed three tasks: SART to measure mind wandering, a perceptual decision task with confidence ratings to measure metacognitive efficiency, and a conflict task to measure cognitive control. Structural Equation Modeling was used to test the interrelations among the three constructs.
He et al. 2023; Tracking resting‐state functional connectivity changes and mind wandering: a longitudinal neuroimaging study	Longitudinal data collection from 122 participants, with assessments at three time points over 2 years	To investigate whether brain functional connectivity could predict mind wandering (MW) and to identify stable associations between brain connectivity and MW.	Functional and structural MRI, self‐reported MW scales (MWFS), Likert‐type MWFS with dimensions SMW, OEMW, UMW	Participants underwent three MRI scans and completed self‐reported MW scales over 2 years. Functional connectivity involving the DMN and FPCN was analyzed for associations with MW frequency across time points. Cross‐lagged effects, group comparisons, and mean connectivity values for LS, MI, and HS groups were examined.
Chen et al. 2022; An effective entropy‐assisted mindwandering detection system with EEG signals based on MM‐SART database	82 participants recruited, 77 participants analyzed (age range: 20–33 years old, 40 females)	To develop an effective mind wandering (MW) detection system using EEG signals and to explore the nonlinear characteristics of EEG signals.	32‐channels electroencephalogram (EEG) signals, photoplethysmography (PPG) signals, galvanic skin response (GSR) signals, eye tracker signals, questionnaires	Participants performed the Sustained Attention to Response Task (SART), EEG, PPG, GSR, and eye tracker signals recorded. Entropy‐based features in time, frequency, and wavelet domains utilized. Random forest classifier used for MW detection with leave‐one‐subject‐out cross‐validation. Techniques of channel and feature selection applied to improve system efficiency and accuracy.
Emadian et al. 2022; Investigating the mediating role of mind‐wandering between achievement motivation and perceived academic stress in nursing students	240 bachelor's nursing students from Islamic Azad University, Sari Branch Faculty of Medicine	To investigate the mediating role of mind wandering between achievement motivation and perceived academic stress in nursing students.	Achievement motivation questionnaire (1970), perceived academic stress questionnaire (2005), mind wandering questionnaire (2013)	Descriptive correlation and structural equation modeling conducted. Data collected using three questionnaires and analyzed using SPSS.22 and Amos.22 software with Pearson correlation coefficient, fitness indices, maximum likelihood estimation, and bootstrap methods.
Arch et al. 2020; Off‐task thinking among adults with and without social anxiety disorder: an ecological momentary assessment study	53 participants from the greater Boulder, Colorado community: 25 adults with SAD and 28 healthy controls (HC), matched for gender, age, race, and ethnicity	To assess off‐task and on‐task thoughts in adults with social anxiety disorder (SAD) and demographically matched controls, and to understand the content and mood correlates of off‐task thoughts in SAD.	Ecological momentary assessment (EMA) of off‐task and on‐task thoughts, trait measure of unintentional and intentional mind wandering	Participants completed EMA to assess off‐task and on‐task thoughts and a trait measure of unintentional and intentional mind wandering. Data analyzed for differences between groups and associations between thought content, mood, and task focus.
Ottaviani et al. 2013b; Flexibility as the key for somatic health: from mind wandering to perseverative cognition	73 healthy university students: 31 men (mean age 25.2) and 42 women (mean age 23.4)	To compare the cardiac and cognitive correlates of perseverative cognition (PC) and mind wandering (MW).	Electrocardiogram (ECG), thought probes, personality questionnaires	Participants engaged in two 5‐min recall interviews (neutral and personal event) and performed a 20‐min tracking task with thought probes while ECG was recorded. Thought probes assessed focus on task, distraction by external stimuli, MW, worry, and rumination. HR and HRV measured.
Huijser et al. 2018; —The wandering self: tracking distracting self‐generated thought in a cognitively demanding context	38 native Dutch speakers (32 after exclusions: 18 female, Mage = 22.4, SDage = 2.6)	Investigated how self‐referential processing (SRP) affected self‐generated thought in a complex working memory task (CWM) to test predictions of a computational cognitive model.	Self‐referential processing (SRP) instigation of self‐generated thought; Eye movement and pupil size	| Spatial CWM task, eye‐tracking to examine rehearsal interference in eye‐movement, self‐generated thinking in pupil size, and analyzed data from 32 participants after exclusions based on performance and data loss.
Liu et al. 2023; —Spontaneous mind wandering impairs model‐based decision making	33 right‐handed healthy subjects (17 females; mean age: 24.6 years, SD = 3.5)	Investigated if and how model‐based vs. model‐free decision making is reduced by trait spontaneous mind wandering.	Trait spontaneous mind wandering's effect on model‐based vs. model‐free decisions	Sequential two‐step Markov decision task, self‐report questionnaire assessing trait spontaneous and deliberate mind wandering, computational neurocognitive dual‐control model of decision making, 201 main task trials, and breaks scenarios.
Franklin et al. 2013; —Window to the wandering mind: pupillometry of spontaneous thought while reading	28 undergraduates (8 male; mean age = 19.43 years, SD = 1.54)	Examined whether pupil dilation (PD) systematically varies within a subject as they report becoming disengaged from a task, to inform theories of mind wandering and lead to interventions that improve task focus.	Pupil dilation (PD) prior to on‐task vs. off‐task reading	| PD measured while participants advanced through a passage one word at a time, spontaneous mind wandering assessed using thought probe methodology, 26 thought probes at pseudorandom intervals, participants asked if they were mind wandering.
Salavera and Usán 2020; —The mediating role of affects between mind‐wandering and happiness	270 university students (133 men, 137 women)	Assessed the mediating role of affects between mind wandering and happiness.	Mind Wandering Questionnaire (MWQ), Positive and Negative Affect Questionnaire (PANAS), SHS subjective happiness scale	Universities were contacted for cooperation, participants filled out questionnaires, data collected in October and November 2019, individual differences compared, dependent and independent variables recorded, and data treated anonymously.
Plimpton et al. 2015; —Role of triggers and dysphoria in mind‐wandering about past, present and future: a laboratory study	40 university students (19 dysphoric, 21 nondysphoric)	Compared the frequency and temporal focus of task‐unrelated thoughts about past, present, and future between dysphoric and nondysphoric participants, using a modified laboratory method for studying IAMs.	Task‐unrelated thoughts about past, present, and future; dysphoria impact on mind wandering	Participants completed a vigilance task with thought probes, recorded their thoughts, rated concentration and vividness of thoughts, and categorized thoughts as past memory, future event, or current situation.
Baird et al. 2014; —The decoupled mind: mind‐wandering disrupts cortical phase‐locking to perceptual events	21 participants (nine men, 12 women; age range = 19–24 years)	Investigated the possibility that task‐unrelated thought is associated with a reduction in the trial‐to‐trial phase consistency of the oscillatory neural signal in response to perceptual input.	P1 ERP reduction, theta‐band cortical phase‐locking decrease, task focus vs. mind wandering reports	Experience sampling paradigm, high‐density electroencephalography (EEG), 0—back vigilance task with visual stimuli, EEG recordings from 128 electrodes, pseudorandom thought prompts, participants asked to self‐report their attentional state.
Seli et al. 2013; —How few and far between? Examining the effects of probe rate on self‐reported mind wandering	104 participants (101 from the United States, 62 female; mean age = 41.7 years, SD = 13.7, range = 19–68)	Examined whether the temporal rate at which thought probes are presented affects the likelihood that people will report periods of mind wandering.	Self‐reported mind wandering frequency based on probe rate	Sustained‐attention task (Metronome Response Task) with intermittent thought probes, varying average time between probes, participants completed HIT on Amazon Mechanical Turk, trait mind wandering assessed via questionnaires.
Schurer et al. 2020; —Working memory capacity but not prior knowledge impact on readers’ attention and text comprehension	90 students (55 women; mean age = 23.80 years, SD = 3.10)	Explored the interaction of working memory capacity (WMC), prior knowledge, and text coherence on reading comprehension and mind wandering.	Self‐reported mind wandering frequency, text comprehension test scores, WMC measures (Ospan and Rspan tasks)	Participants read coherent or incoherent hypertext about copyright law, reported mind wandering during reading, completed reading comprehension and memory tests, WMC assessed, data collected in a single 1.5‐h session, procedures followed Helsinki Declaration guidelines.
Chaieb et al. 2022b; —Modulation of mind wandering using monaural beat stimulation in subjects with high trait‐level mind wandering	34 preselected subjects with high trait‐level mind wandering (mean age 25.7 ± 0.8, 23 female)	Investigated whether mind wandering (MW) can be reduced by monaural theta beats in subjects with high trait‐level MW, as indicated by an online MW questionnaire.	Propensity to mind wander, meta‐awareness, temporal orientation of MW during different auditory beat stimulation conditions	Subjects performed sustained attention to response task (SART) with thought‐probes assessing MW, meta‐awareness, and temporal orientation of MW, with monaural theta beats, sine tones, and silence as control conditions.
Kirk et al. 2018; —On‐the‐spot binaural beats and mindfulness reduces behavioral markers of mind wandering	81 healthy volunteers (Mindfulness group: 25 participants; Binaural beats group: 27 participants; Control group: 25 participants)	Investigated whether laboratory evidence of mind wandering can be reduced through two on‐the‐spot interventions: mindfulness meditation for 15 min and binaural auditory beats for 15 min.	Behavioral markers of mind wandering using the Sustained Attention to Response Task (SART)	Participants completed two testing sessions (baseline and postintervention), received either mindfulness meditation, binaural beats, or no intervention, tested on SART for mind wandering, and procedures were conducted in accordance with ethical guidelines.
Iglesias‐Arro et al. 2020; —Introspective and neurophysiological measures of mind wandering in schizophrenia	22 adults with schizophrenia spectrum disorders (Group: SZQ); 23 healthy controls (Group: CTRL)	Explored mind wandering in schizophrenia, hypothesizing a predominance of mind wandering as a core dysfunction in this disorder.	Frequency of mind wandering, verbal reports, EEG complexity patterns	Participants watched synchronized and nonsynchronized videoclips, provided verbal reports, EEG data recorded, cognitive evaluation using SCIP‐S and D2 Test of Attention, data analyzed for mind wandering frequency and EEG complexity patterns.
Zavagnin et al. 2014; —When the mind wanders: age‐related differences between young and older adults	59 participants (20 young adults, 20 young–old adults, 19 old–old adults; native Italian speakers)	Assessed age‐related differences in mind wandering (MW) between young, young–old, and old–old adults using two versions of the sustained attention to response task (SART).	Reported MW episodes, response time latency and variability, incorrect response, omission errors, MW questionnaire	Participants tested individually in a 90‐min session, tasks included health and demographics questionnaire, vocabulary test, two versions of SART (semantic and perceptual), processing speed task (pattern comparison), working memory task, MW questionnaire.
Welhaf et al. 2022; —A “Goldilocks zone” for mind‐wandering reports? A secondary data analysis of how few thought probes are enough for reliable and valid measurement	Study 1: 541 participants; Study 2: ∼260 participants per condition	Investigated how many thought probes are necessary to reliably and validly assess individual differences in mind wandering propensity without disrupting thought flow.	Thought‐unrelated thought (TUT) rate, correlations with working memory capacity, attention‐control ability, disorganized schizotypy, self‐reported mind wandering	Participants completed various cognitive tasks, received thought probes, probes were analyzed to determine the minimum number needed for reliable TUT rate measurement, random selection of probes for each subject in increments of two.
Zhang et al. 2022; —The relationship between schizotypal traits and satisfaction with life among Chinese young adults: the mediating effect of trait anxiety and mind wandering	206 participants (102 with high schizotypal traits, 104 with low schizotypal traits)	Investigated the association between schizotypal traits and satisfaction with life, exploring the mediating roles of trait anxiety and mind wandering.	Schizotypal Personality Questionnaire, Satisfaction with Life Scale, Trait Anxiety Inventory, Mind Wandering Questionnaire	Participants completed SPQ online, those with high and low schizotypal traits were selected, completed additional questionnaires, data analyzed for the mediating effects of trait anxiety and mind wandering on the relationship between schizotypal traits and satisfaction with life.
Carciofo 2022; —A time to wander: exploring associations between components of circadian functioning, mind wandering typology, and time‐of‐day	265 university students (aged 18–33, mean = 20.78)	Investigated the association between components of circadian functioning, mind wandering (MW) typology, and time‐of‐day.	Morning Affect, Eveningness, Distinctness, deliberate and spontaneous MW, problem‐solving daydreams, sleep quality, personality, affect, life satisfaction	Participants completed an online survey including various scales (Mind Wandering‐Deliberate and Spontaneous, Problem‐solving Daydreams, MESSi, PSQI, PANAS, BFI‐10, and Students’ Life Satisfaction Scale), reported peak time for daily MW and problem‐solving daydreams.
Polychroni et al. 2023; —Response time fluctuations in the sustained attention to response task predict performance accuracy and meta‐awareness of attentional states	74 healthy controls (48 females, age range: 18–48, Mage = 24.5, SD = 7.1)	Investigated how response time (RT) patterns in the Sustained Attention to Response Task (SART) relate to performance accuracy and meta‐awareness of attentional states.	Response time patterns, self‐reported attentional state, meta‐awareness of attentional states	Participants completed the SART with interstimulus intervals, responded to frequent nontargets and withheld responses to infrequent targets, answered thought probes on attentional state and meta‐awareness, data analyzed for RT patterns, performance accuracy, and meta‐awareness.

### 3.2 Key Themes and Findings

The findings were organized into thematic sections based on the core dimensions of mind wandering, as outlined in the *MAMW framework*. Each theme is discussed below, with subheadings to structure the presentation of results.

#### 3.2.1 Self‐Report and Real‐Time Data Collection

Questionnaires and ESMs were the most frequently used methods, accounting for 42.40% and 16.67% of occurrences, respectively. Questionnaires, such as the *Mind Wandering Questionnaire (MWQ)*, were praised for their *high feasibility and ecological validity*, making them suitable for large‐scale studies (Trigueros et al. 2019). However, their reliance on self‐reporting introduced biases, such as *recall inaccuracies and social desirability effects* (Teessar 2024; Smallwood and Schooler 2015). ESM, including thought probes and ecological momentary assessment, addressed some of these limitations by capturing mind wandering in real‐time (Marcusson‐Clavertz et al. 2023; Chaieb and Hoppe 2022a; Crawford et al. 2022). These methods provided a more dynamic and accurate picture of mind wandering but were sometimes *intrusive*, potentially disrupting the natural flow of thought (Kane et al. 2017).

#### 3.2.2 Neural Correlates of Mind Wandering

Neuroimaging techniques, such as *functional magnetic resonance imaging (fMRI) and electroencephalography (EEG)*, were used in 19.31% of the reviewed studies to investigate the neural underpinnings of mind wandering. These studies consistently identified the *DMN* as a key neural correlate of spontaneous mind wandering (Menon 2023; Christoff et al. 2016). For example, fMRI studies revealed *increased DMN activity* during mind wandering episodes, particularly in the *medial prefrontal cortex (mPFC) and posterior cingulate cortex (PCC*; Kucyi et al. 2014; Turnbull et al. 2019). EEG studies complemented these findings by demonstrating distinct neural signatures, such as *increased theta and alpha oscillations*, during mind wandering (Lu and Rodriguez‐Larios 2022; Kam et al. 2021). However, neuroimaging methods were limited by their *high cost, lack of ecological validity, and reliance on controlled laboratory settings* (van Atteveldt et al. 2018; Smallwood et al. 2021; Mittner et al. 2016).

#### 3.2.3 Behavioral and Cognitive Manifestations

Behavioral performance measures, such as the *Go/No‐Go Task and working memory tasks*, were employed in 17.78% of the studies. These measures provided objective data on task performance and attentional lapses, revealing that mind wandering is associated with *reduced accuracy and slower reaction times* (Seli et al. 2018; McVay and Kane 2013). For instance, the Go/No‐Go Task demonstrated that mind wandering increases the likelihood of errors in inhibitory control tasks (Unsworth and Robison 2020; Jana and Aron 2022. However, behavioral measures were often *context‐specific and lacked sensitivity* to detect subtle or spontaneous instances of mind wandering, limiting their applicability in real‐world settings (Seli et al. 2018; Smallwood and Schooler 2015).

#### 3.2.4 Physiological and Visual Attention Measures

Physiological measures (e.g., heart rate, pupillometry) and eye‐tracking were less frequently used, representing 4.58% and 2.73% of occurrences, respectively. These methods offered unique insights into the bodily and attentional correlates of mind wandering (Kam et al. 2022). For example, pupillometry studies demonstrated that mind wandering is associated with *reduced pupil dilation*, reflecting decreased cognitive effort (Pelagatti et al. 2025; Zhao et al. 2019). Eye‐tracking studies revealed distinct gaze patterns during mind wandering, such as *increased fixation duration and reduced saccadic activity* (Hooge et al. 2025; Lee et al. 2021; Faber et al. 2020). However, these methods often required *complex data analysis and interpretation*, limiting their accessibility and practicality (Tables 3 and 4).

**TABLE 3:** Methods for studying mind wandering.

Core dimension	Method	Mind wandering subtypes	Validity	Reliability	Sensitivity	Specificity	Feasibility	Ecological validity
Temporal dynamics	Experience sampling (ESM)	Spontaneous, task‐unrelated thought (TUT), intentional mind wandering	High (captures real‐time data)	Moderate (depends on participant compliance)	High (detects subtle, spontaneous episodes)	Moderate (may capture other cognitive states)	Moderate (requires frequent participant engagement)	High (reflects real‐world settings)
	Thought probes	Spontaneous, task‐unrelated thought (TUT)	High (directly assesses mind wandering)	High (consistent across trials)	High (targets specific moments)	High (focused on mind wandering)	High (easy to administer)	Moderate (can be intrusive)
Content and context	Questionnaires	Task‐unrelated thought (TUT), intentional mind wandering, stimulus‐independent thought	Moderate (subject to recall bias)	High (consistent across administrations)	Moderate (may miss subtle episodes)	Moderate (may conflate mind wandering with other states)	High (easy to administer to large groups)	High (applicable to real‐world contexts)
	Ecological momentary assessment (EMA)	Spontaneous, task‐unrelated thought (TUT), intentional mind wandering	High (captures context‐specific data)	Moderate (depends on participant compliance)	High (real‐time reporting)	Moderate (may include unrelated thoughts)	Moderate (requires frequent engagement)	High (reflects real‐world settings)
Neural and physiological correlates	fMRI	Default mode network (DMN) activity, spontaneous thought	High (directly measures brain activity)	High (consistent across sessions)	High (detects neural patterns)	High (specific to neural activity)	Low (costly, requires specialized equipment)	Low (laboratory‐based)
	EEG	Spontaneous thought, attentional lapses	High (measures real‐time brain activity)	High (consistent across sessions)	High (detects rapid neural changes)	High (specific to neural activity)	Moderate (requires specialized equipment)	Low (laboratory‐based)
	Physiological measures (e.g., heart rate, pupillometry)	Attentional lapses, arousal‐related mind wandering	Moderate (indirect measure of mind wandering)	Moderate (subject to external influences)	Moderate (detects autonomic changes)	Moderate (may reflect general arousal)	Moderate (requires specialized equipment)	Moderate (can be used in dynamic settings)
Behavioral and cognitive manifestations	Behavioral tasks (e.g., Go/No‐Go)	Task‐related lapses, attentional failures	High (objective measure of performance)	High (consistent across trials)	Moderate (may miss spontaneous episodes)	High (specific to task‐related lapses)	High (easy to administer)	Low (laboratory‐based)
	Eye‐tracking	Attentional shifts, visual attention during mind wandering	High (directly measures visual attention)	High (consistent across trials)	High (detects attentional shifts)	High (specific to gaze patterns)	Moderate (requires specialized equipment)	Moderate (can be used in seminaturalistic settings)
Subjective experience	Self‐reports	Task‐unrelated thought (TUT), intentional mind wandering, stimulus‐independent thought	Moderate (subject to recall bias)	High (consistent across administrations)	Moderate (may miss subtle episodes)	Moderate (may conflate mind wandering with other states)	High (easy to administer)	High (applicable to real‐world contexts)
	Mindfulness scales	Meta‐awareness of mind wandering, intentional mind wandering	Moderate (indirect measure of awareness)	High (consistent across administrations)	Moderate (may not capture all aspects of mind wandering)	Moderate (may reflect general mindfulness)	High (easy to administer)	High (applicable to real‐world contexts)

**TABLE 4:** Comparative overview of methods for assessing mind wandering.

Category	Methods	Strengths	Limitations	Occurrence (%)
Self‐report and real‐time data collection	‐ Questionnaires (e.g., MWQ)—experience sampling methods (ESM)	‐ High feasibility and ecological validity (suitable for large‐scale studies)—ESM captures real‐time data for better accuracy.	‐ Questionnaires prone to biases (recall inaccuracies, social desirability effects)—ESM can be intrusive.	42.40 (Questionnaires), 16.67 (ESM)
Neural correlates	‐ Functional MRI (fMRI)—electroencephalography (EEG)	‐ Identified key neural correlates like the Default Mode Network (DMN)—Revealed DMN activity in mPFC, PCC, and so forth—Unique neural signatures (e.g., theta, alpha oscillations).	‐ High cost—Lack of ecological validity—Reliance on controlled lab settings.	19.31
Behavioral and cognitive manifestations	‐ Behavioral performance measures (e.g., Go/No‐Go Task, working memory tasks)	‐ Provided objective data on task‐related lapses—Demonstrated impact of mind wandering on accuracy and reaction times.	‐ Context‐specific—Limited ability to detect spontaneous instances of mind wandering.	17.78
Physiological and visual attention measures	‐ Physiological measures (e.g., heart rate, pupillometry)—eye‐tracking	‐ Unique insights into bodily and attentional correlates—Pupil dilation linked to decreased cognitive effort; gaze patterns identified through eye‐tracking.	‐ Complex data analysis and interpretation—Limited accessibility and practicality.	4.58 (Physiological), 2.73 (eye‐tracking)

A summary of the key characteristics, strengths, limitations, and prevalence of different methodologies used in the reviewed studies for assessing mind wandering.

#### 3.2.5 Description, Evaluation, and Comparisons Across Methods

The methods used in the included studies were categorized into five subtypes based on their characteristics and features. The first category, *self‐report and real‐time data collection*, includes questionnaires, probe‐catching, thought sampling, and ESMs. *Questionnaires* are retrospective self‐report tools that assess the frequency and content of mind wandering over a specified period (Schubert et al. 2024). *Probe‐catching* involves interrupting participants during a task to ask whether they were mind wandering at that moment, providing real‐time data on mind wandering episodes (Chu et al. 2023; Weinstein 2018). *Thought sampling*, a variant of probe‐catching, goes a step further by asking participants to report the content of their thoughts, offering insights into the nature of mind wandering (Wiemers et al. 2019). *ESM* captures mind wandering in real‐world settings by prompting participants to report their thoughts and experiences at random intervals throughout the day (Tschacher et al. 2021). While ESM shares similarities with probe‐catching, it emphasizes ecological validity by assessing mind wandering in natural environments rather than controlled laboratory settings (Kawashima et al. 2023).

The second category, *brain activity and structure*, includes neuroimaging techniques such as fMRI and EEG, which provide objective data on the neural correlates of mind wandering, particularly within the DMN (Kam et al. 2022). The third category, *behavioral and cognitive assessment*, encompasses behavioral performance measures like the Go/No‐Go Task and working memory tasks, which assess task‐related attentional lapses and their impact on performance (Unsworth et al. 2022; Wiemers et al. 2019). The fourth category, *bodily responses*, includes physiological measures such as heart rate and pupillometry, which capture bodily correlates of mind wandering episodes, such as reduced pupil dilation reflecting decreased cognitive effort (Ahmadi et al. 2024). The fifth category, *visual attention and perception*, involves eye‐tracking techniques that provide valuable data on attention shifts and perceptual dynamics during mind wandering, revealing distinct gaze patterns like increased fixation duration and reduced saccadic activity (Hooge et al. 2025).

To evaluate the accuracy, strengths, and limitations of these methods, we conducted a *qualitative synthesis* of the findings, identifying common themes and patterns across studies. We compared the methods based on key criteria, including *validity, reliability, sensitivity, specificity, feasibility*, and *ecological validity*. For *self‐report and real‐time data collection*, we compared the ecological validity and feasibility of questionnaires and ESM with their susceptibility to self‐report biases, such as recall inaccuracies and social desirability effects. For *brain activity and structure*, we analyzed the neural correlates of mind wandering, particularly the role of the DMN, and compared the precision and ecological validity of neuroimaging techniques like fMRI and EEG. For *behavioral and cognitive assessment*, we evaluated the sensitivity of behavioral tasks, such as the Go/No‐Go Task, in detecting task‐related attentional lapses and compared their ability to capture spontaneous versus deliberate mind wandering. For *bodily responses*, we examined the specificity of physiological measures, such as heart rate and pupillometry, in capturing bodily correlates of mind wandering and compared their practicality in real‐world settings. For *visual attention and perception*, we assessed the reliability of eye‐tracking techniques in detecting attention shifts and compared their applicability across different contexts.

These analyses revealed that no single method is universally superior, as each approach has unique strengths and limitations (Malefaki et al. 2025; Riantiningtyas et al. 2024). For example, while neuroimaging provides precise neural data, it lacks ecological validity (Gadassi‐Polack et al. 2024), whereas ESM offers high ecological validity but is prone to self‐report biases (Verhagen 2016). To further understand the relative accuracy of different methods, we conducted *cross‐subtype comparisons* based on key criteria, including validity, reliability, sensitivity, specificity, feasibility, and ecological validity. These comparisons were guided by the *MAMW framework*, which emphasizes the integration of diverse methodologies to capture the full spectrum of mind wandering.

We compared the *validity* of each method by assessing the extent to which it measures what it intends to measure. For example, neuroimaging techniques excel in capturing neural correlates but may not fully reflect the subjective experience of mind wandering (Gruberger et al. 2011), whereas self‐reports capture subjective experiences but lack objectivity (Harris et al. 2023). We assessed the *reliability* of each method by evaluating the consistency and stability of its results. Behavioral tasks, for instance, showed high reliability in detecting task‐related lapses (Fetsch 2016) but were less consistent in capturing spontaneous mind wandering. We evaluated the *sensitivity* of each method by examining its ability to detect subtle or spontaneous instances of mind wandering. Physiological measures, such as pupillometry, demonstrated high sensitivity but required complex data analysis (Mathôt 2018). We examined the *specificity* of each method by assessing its ability to distinguish mind wandering from other cognitive states. Eye‐tracking, for example, showed high specificity in detecting attention shifts but was less effective in capturing the content of mind wandering (Lee et al. 2021). We compared the *feasibility* of each method by evaluating its practicality in terms of cost, time, and expertise. Questionnaires and ESM were highly feasible for large‐scale studies (Schulz et al. 2020; Verhagen 2016), whereas neuroimaging and physiological measures were more resource‐intensive (Kupers et al. 2024). Finally, we assessed the *ecological validity* of each method by determining the extent to which it reflects real‐world mind wandering. ESM and questionnaires scored high on ecological validity (Eisele et al. 2025), whereas laboratory‐based methods like neuroimaging and behavioral tasks scored lower (Sui et al. 2020).

These comparisons highlighted the need for a *multidimensional approach* to measuring mind wandering, as no single method excels across all criteria. The MAMW framework addresses this need by integrating diverse methodologies, enabling researchers to balance precision, ecological validity, and practicality. These diverse methodologies also allowed researchers to explore critical themes, such as *temporal dynamics*, *content and context, neural and physiological correlates, behavioral and cognitive manifestations*, and *subjective experience*, shedding light on the multifaceted nature of mind wandering.

## 4 Discussion

This systematic review evaluated the accuracy, strengths, and limitations of methods for measuring mind wandering subtypes, synthesizing findings from a diverse body of literature (Table 3). The results revealed that no single method is universally superior, as each approach offers unique insights while also presenting significant limitations. Self‐report measures, such as questionnaires and ESMs, are widely used due to their feasibility and ecological validity, but they are prone to biases such as recall inaccuracies and social desirability effects. Neuroimaging techniques, while providing objective data on neural correlates like the DMN, are constrained by high costs and a lack of ecological validity. Behavioral tasks offer valuable insights into task‐related attentional lapses but often fail to capture spontaneous or subtle instances of mind wandering. Physiological measures and eye‐tracking provide unique perspectives on bodily and attentional correlates but require complex data analysis, limiting their accessibility and practicality (Table 5).

**TABLE 5:** Subtypes of mind wandering.

Subtype	Definition	Key features	Context	Measurement methods	Neural basis	Impact on task performance
Spontaneous thought	Unintentional, freely flowing thoughts.	Broad scope, includes daydreaming and creative thinking.	Can occur at rest or during tasks.	Self‐reports, neuroimaging (e.g., fMRI), thought sampling.	Default mode network (DMN).	May or may not interfere with tasks.
Task‐unrelated thought (TUT)	Thoughts unrelated to the current task.	Occurs during task performance, often disrupts focus.	During task performance.	Thought probes, self‐reports, behavioral tasks.	DMN and task‐positive networks.	Typically disrupts task performance.
Intentional mind wandering	Deliberate shifting of attention away from the task.	Conscious decision to disengage from the task.	During task performance or idle moments.	Self‐reports, thought probes.	DMN and executive control networks.	May or may not disrupt tasks, depending on intent.
Stimulus‐independent thought	Thoughts not triggered by external stimuli.	Internally generated, self‐referential.	Can occur at rest or during tasks.	Self‐reports, neuroimaging, thought sampling.	Default mode network (DMN).	May interfere with tasks if task‐related.
Attentional lapses	Brief failures in maintaining focus on a task.	Short‐lived, often unintentional.	During task performance.	Behavioral tasks (e.g., Go/No‐Go), eye‐tracking.	DMN and dorsal attention network (DAN).	Disrupts task performance temporarily.
Arousal‐related mind wandering	Mind wandering influenced by changes in physiological arousal.	Linked to autonomic responses (e.g., heart rate, pupillometry).	During tasks or rest.	Physiological measures (e.g., heart rate, skin conductance).	DMN and autonomic nervous system activity.	May disrupt tasks if arousal levels are suboptimal.
Attentional failures	Prolonged or significant lapses in attention.	More severe than attentional lapses, often leading to task errors.	During task performance.	Behavioral tasks, self‐reports.	DMN and task‐positive networks.	Significantly disrupts task performance.
Attentional shifts	Movement of attention from one focus to another.	Can be intentional or unintentional.	During tasks or rest.	Eye‐tracking, behavioral tasks.	Dorsal attention network (DAN) and DMN.	May or may not disrupt tasks, depending on context.

**Spontaneous thought** is the broadest category, encompassing all internally generated thoughts, including TUT, intentional mind wandering, and stimulus‐independent thought. **Task‐unrelated thought (TUT)** is a specific subtype that occurs during task performance and is often disruptive. **Intentional mind wandering** involves a deliberate shift in attention, distinguishing it from spontaneous or unintentional mind wandering. **Stimulus‐independent thought** focuses on internally generated thoughts, regardless of external triggers. **Attentional lapses** and **Attentional failures** represent varying degrees of attention disruption, with the latter being more severe. **Arousal‐related mind wandering** links mind wandering to physiological states, offering a unique perspective on its triggers and consequences. **Attentional shifts** can be intentional or unintentional and are often studied using eye‐tracking or behavioral tasks.

### 4.1 Integration of Methods

The review highlighted that no single method is universally superior, as each approach has its own strengths and limitations. The choice of method depends on the research context and objectives. For example, laboratory studies often favor neuroimaging and behavioral performance measures for their precision and objectivity, while real‐world studies benefit from the ecological validity of ESMs and questionnaires. In clinical applications, combining multiple methods—such as self‐reports with neuroimaging or physiological measures—can provide a more comprehensive understanding of mind wandering, particularly in populations where it may have diagnostic or therapeutic implications. This multidimensional approach aligns with *the MAMW framework*, which emphasizes the importance of combining diverse methodologies to capture the complex and multifaceted nature of mind wandering. By adopting this framework, researchers can better address the dynamic and variable nature of mind wandering, ultimately enhancing the validity, reliability, and applicability of their findings.

To address these challenges, we proposed the MAMW framework, which integrates diverse methodologies to provide a holistic understanding of mind wandering. The MAMW framework bridges the gap between theoretical characterization and practical measurement by emphasizing the interplay between cognitive processes, neural mechanisms, contextual factors, and measurement methods. The framework also highlights the importance of contextual factors, such as task demands and environmental stimuli, in shaping the frequency and impact of mind wandering. By combining subjective and objective measures, the MAMW framework balances precision with ecological validity, enabling researchers to capture both the experiential and mechanistic dimensions of mind wandering.

The MAMW framework has significant implications for theory, methodology, and practice. Theoretically, it advances our understanding of the cognitive, neural, and contextual dimensions of mind wandering, highlighting the interplay between spontaneous and deliberate subtypes and their adaptive and maladaptive outcomes. Methodologically, it addresses the limitations of individual methods by enabling researchers to triangulate data from multiple sources, enhancing the validity, reliability, and ecological validity of findings. Practically, the framework supports targeted interventions in clinical, educational, and workplace settings. For example, in clinical populations such as individuals with ADHD or depression, the framework can inform diagnostic tools and therapeutic strategies by identifying specific mind wandering patterns and their neural correlates. In educational and workplace contexts, it can guide the development of interventions to improve focus, productivity, and well‐being.

Future research should prioritize several key areas to further advance the study of mind wandering. First, there is a need for standardized definitions and scales for questionnaires and probes to ensure consistency across studies. Second, greater emphasis should be placed on enhancing ecological validity, particularly for laboratory‐based methods, by designing studies that capture mind wandering in real‐world settings. Third, researchers should investigate subtype‐specific mechanisms, such as the neural and behavioral correlates of intentional versus spontaneous mind wandering, to better understand their distinct features and implications. Fourth, longitudinal studies are needed to track the developmental and clinical trajectories of mind wandering, particularly in at‐risk populations. Finally, integrating multiple methodologies will be essential to build a unified understanding of mind wandering, leveraging the strengths of each approach while mitigating their limitations.

*In conclusion*, this systematic review underscores the need for a multidimensional approach to measuring mind wandering, as exemplified by the MAMW framework. By integrating diverse methodologies, the framework provides a comprehensive understanding of mind wandering, balancing theoretical insights with practical measurement challenges. The findings highlight the importance of standardized tools, greater ecological validity, and longitudinal research to advance both scientific knowledge and practical applications. Future studies should prioritize integrating multiple methods to explore the mechanisms, correlates, and consequences of mind wandering across diverse contexts and populations. This integrative approach will not only enhance our understanding of mind wandering but also support targeted interventions in clinical, educational, and workplace settings, ultimately improving outcomes for individuals and organizations alike.

## Author Contributions

**Sholeh Nazari**: conceptualization, investigation, writing–original draft, methodology, validation, visualization, writing–review and editing, software, formal analysis, project administration, data curation. **Paul Fitzgerald**: writing–review and editing, supervision. **Reza Kazemi**: conceptualization, investigation, methodology, validation, visualization, writing–review and editing, project administration, data curation, supervision.

## Peer Review

The peer review history for this article is available at https://publons.com/publon/10.1002/brb3.70764.

## Supporting information




**Supplementary Materials**: brb370764‐sup‐0001‐SuppMatt.docx

## Data Availability

The data that support the findings of this study are available from the corresponding author upon reasonable request.
