# Supplementary material for: The Relative Accuracy of Different Methods for Measuring Mind Wandering Subtypes: A Systematic Review
Source: Brain Behav. 2025 Aug 22;15(8):e70764. doi: 10.1002/brb3.70764 (PMC12373717; doi:10.1002/brb3.70764)
Supplement: Supplementary file 1 — Supplementary Materials: brb370764‐sup‐0001‐SuppMatt.docx [file BRB3-15-e70764-s001.docx]

***Overview of Methodological Approaches and Their Relative Frequencies in Research***

| **Category** | **Method/Technique** | **Occurrences** | **Percentage** |
| --- | --- | --- | --- |
| **Neuroimaging Techniques** | **Total Occurrences: 318 Total Percentage: 19.31%** | | |
|  | fMRI | 75 | (75/1635) × 100 = 4.58% |
|  | EEG Recording | 70 | (70/1635) × 100 = 4.28% |
|  | Event-Related Potentials (ERP) | 30 | (30/1635) × 100 = 1.83% |
|  | Continuous EEG Recording | 1 | (1/1635) × 100 = 0.06% |
|  | Transcranial Direct Current Stimulation (tDCS) | 15 | (15/1635) × 100 = 0.91% |
|  | Multivariate Pattern Analyses (MVPA) | 10 | (10/1635) × 100 = 0.61% |
|  | Resting-state fMRI | 30 | (30/1635) × 100 = 1,83% |
|  | Resting-state EEG | 20 | (20/1635) × 100 = 1.22% |
|  | Functional Connectivity Analyses | 25 | (25/1635) × 100 = 1.52% |
|  | Source Localization (sLORETA) | 12 | (12/1635) × 100 = 0.73% |
|  | MRI Scanning (Anatomical/Functional) | 18 | (18/1635) × 100 = 1.10% |
|  | Magnetoencephalography (MEG) | 6 | (6/1635) × 100 = 0.36% |
|  | Functional Near-Infrared Spectroscopy (fNIRS) | 6 | (6/1635) × 100 = 0.36% |
| **Behavioral Performance Measures** | **Total Occurrences: 292 Total Percentage: 17.78%** | | |
|  | Monotonous Vigilance Tasks | 3 | (3/1635) × 100 = 0.18% |
|  | Go/No-Go Task (Modified Sustained Attention to Response Task (SART), Sustained Attention to Response Task with Faces (F-SART) ) | 120 | (120/1635) × 100 = 7.33% |
|  | Attention Network Test (ANT) | 4 | (4/1635) × 100 = 0.24% |
|  | Driving Simulator (Assetto Corsa) | 6 | (6/1635) × 100 = 0.36% |
|  | Computerized Tasks (n-back, antisaccade, number-letter switching) | 15 | (15/1635) × 100 = 0.91% |
|  | Metronome Response Task (MRT) | 22 | (22/1635) × 100 = 1.34% |
|  | Reading Passages/Task | 22 | (22/1635) × 100 = 1.34% |
|  | Encoding Tasks (Objective/Subjective) | 1 | (1/1635) × 100 = 0.061% |
|  | Memory Retrieval Tasks | 1 | (1/1635) × 100 = 0.061% |
|  | Visual Search Task | 22 | (22/1635) × 100 = 1.34% |
|  | Working Memory Tasks (Ospan, Symspan, Rspan) | 40 | (40/1635) × 100 = 2.44% |
|  | Contrast Change Detection Task | 1 | (1/1635) × 100 = 0.061% |
|  | Shape Expectations Task | 1 | (1/1635) × 100 = 0.061% |
|  | Time-Estimation Task | 1 | (1/1635) × 100 = 0.061% |
|  | Visuomotor Tracking Task | 2 | (2/1635) × 100 = 0.12% |
|  | Task Switching | 15 | (15/1635) × 100 = 0.91% |
|  | RHI Induction (Rubber Hand Illusion) | 1 | (1/1635) × 100 = 0.061% |
|  | Psychomotor Vigilance Task (PVT) | 12 | (12/1635) × 100 = 0.73% |
|  | Texture Discrimination Task (TDT) | 1 | (1/1635) × 100 = 0.061% |
|  | Compound Remote Associates Test (CRA) | 1 | (1/1635) × 100 = 0.061% |
|  | Virtual Reality (VR) Tasks | 1 | (1/1635) × 100 = 0.061% |
| **Questionnaires** | **Total Occurrences: 589 Total Percentage: 42.401%** | | |
|  | Beck Depression Inventory (BDI-II) | 45 | (45/1635) × 100 = 8.93% |
|  | Mindfulness Observing and Describing Questionnaire (MODQ) | 1 | (1/1635) × 100 = 0.06% |
|  | Amsterdam Resting State Questionnaire (ARSQ) | 25 | (25/1635) × 100 = 1.52% |
|  | Daydreaming Frequency Scale (DDFS) | 25 | (25/1635) × 100 = 1.52% |
|  | Stanford Sleepiness Scale (SSS) | 2 | (2/1635) × 100 = 0.40% |
|  | Karolinska Sleepiness Scale (KSS) | 1 | (1/1635) × 100 = 0.06% |
|  | Thought Characteristics Questionnaire (TCQ) | 1 | (1/1635) × 100 = 0.06% |
|  | Insomnia Severity Index (ISI) | 25 | (25/1635) × 100 = 1.52% |
|  | Montreal Cognitive Assessment (MoCA) | 12 | (12/1635) × 100 = 0.73% |
|  | Unified Parkinson’s Disease Rating Scale (MDS-UPDRS) | 1 | (1/1635) × 100 = 0.06% |
|  | Levodopa Equivalent Daily Dose (LEDD) | 1 | (1/1635) × 100 = 0.06% |
|  | Mindfulness Attention and Awareness Scale (MAAS) | 35 | (35/1635) × 100 = 2.14% |
|  | Dundee Stress State Questionnaire (DSSQ) | 30 | (30/1635) × 100 = 1.83% |
|  | NASA–Task Load Index | 3 | (3/1635) × 100 = 0.18% |
|  | Self-Report Questionnaires | 270 | (270/1635) × 100 = 16.51% |
|  | Mind Wandering Questionnaire (MWQ) | 35 | (35/1635) × 100 = 2.14% |
|  | Fatigue Assessment Scale (FAS) | 1 | (1/1635) × 100 = 0.06% |
|  | Ruminative Response Scale | 22 | (22/1635) × 100 = 1.34% |
|  | Hospital Anxiety and Depression Scale (HADS) | 10 | (10/1635) × 100 = 0.61% |
|  | Positive and Negative Affect Scale (PANAS) | 22 | (22/1635) × 100 = 1.34% |
|  | Schizotypal Personality Questionnaire (SPQ) | 8 | (8/1635) × 100 = 0.48% |
|  | Obsessive-Compulsive Inventory | 1 | (1/1635) × 100 = 0.06% |
|  | Pathological Narcissism Inventory (PNI) | 1 | (1/1635) × 100 = 0.06% |
|  | Big Five Personality Inventory (BFI-10) | 10 | (10/1635) × 100 = 0.61% |
|  | Athlete Mind Wandering Scale | 1 | (1/1635) × 100 = 0.06% |
|  | Cognitive Flexibility Inventory (CFI) | 1 | (1/1635) × 100 = 0.06% |
| **Experience Sampling** | **Total Occurrences: 273 Total Percentage: 16.67%** | | |
|  | Thought Probes | 200 | (200/1635) × 100 = 12.23% |
|  | Daily-Life Experience-Sampling Study | 22 | (22/1635) × 100 = 1.34% |
|  | Online Thought Probes | 25 | (25/1635) × 100 = 1.52% |
|  | Free Association-Based Thought Sampling Task (FAST) | 2 | (2/1635) × 100 = 0.12% |
|  | Ecological Momentary Assessment | 22 | (22/1635) × 100 = 1.34% |
|  | Daily Logs | 1 | (1/1635) × 100 = 0.06% |
|  | MetricWire App (Experience Sampling) | 1 | (1/1635) × 100 = 0.06% |
| **Physiological Measures** | **Total Occurrences: 45 Total Percentage: 2.73%** | | |
|  | Cardiac Activity Assessment | 22 | (22/1635) × 100 = 1.34% |
|  | Electrocardiogram (ECG) | 15 | (15/1635) × 100 = 0.91% |
|  | Pupillometry (Eye-Tracking) | 8 | (8/1635) × 100 = 0.48% |
| **Eye-Tracking** | **Total Occurrences: 75 Total Percentage: 4.58%** | | |
|  | Eye-Tracking | 75 | (75/1635) × 100 = 4.58% |
| **Task-Based Measures** | **Total Occurrences: 43 Total Percentage: 2.613%** | | |
|  | Machine-Learning Classifier | 15 | (15/1635) × 100 = 0.91% |
|  | Psychophysics Toolbox | 25 | (25/1635) × 100 = 1.52% |
|  | Neuropsychological Test Battery | 1 | (1/1635) × 100 = 0.061% |
|  | Content Validity Assessment by Expert Panels | 1 | (1/1635) × 100 = 0.061% |
|  | Cursor Movement Tracking | 1 | (1/1635) × 100 = 0.061% |
|  |  |  |  |
|  |  |  |  |
|  |  |  |  |
|  |  |  |  |
